# Assessing the evidence on the differential impact of menthol versus non-menthol cigarette use on smoking cessation in the U.S. population: a systematic review and meta-analysis

**DOI:** 10.1186/s13011-021-00397-4

**Published:** 2021-08-11

**Authors:** Mimi M. Kim, Geoffrey M. Curtin

**Affiliations:** Scientific & Regulatory Affairs, RAI Services Company, 401 North Main Street, Winston-Salem, NC 27101 USA

**Keywords:** Smoking, Menthol cigarettes, Systematic reviews, Meta-analysis, Smoking cessation

## Abstract

**Background:**

The potential impact of menthol versus non-menthol cigarette use on smoking behaviors is an intensely scrutinized topic in the public health arena. To date, several general literature reviews have been conducted, but findings and conclusions have been discordant. This systematic review followed PRISMA guidelines to examine the Key Question, “Does menthol cigarette use have a differential impact on smoking cessation compared with non-menthol cigarette use?”

**Methods:**

Six databases—Cochrane Central Register of Controlled Trials, Cochrane Database of Systematic Reviews, Database of Abstracts of Reviews of Effects, MEDLINE, Embase and PsycInfo—were queried from inception to June 12, 2020. Articles comparing menthol versus non-menthol cigarette smokers in terms of at least one predefined smoking cessation outcome were included. Risk of bias was assessed using the Agency for Healthcare Research and Quality Evidence-Based Practice Center approach. A random-effects model utilizing the DerSimonian and Laird method to pool adjusted odds ratio was applied. Variations among pooled studies were assessed using Cochran’s Q statistic, and heterogeneity was quantified using the inconsistency index (I^2^).

**Results:**

Forty-three demographically adjusted studies (22 rated “good”, 20 rated “fair”, and one study rated “poor” individual study quality) comparing menthol and non-menthol smokers were qualitatively synthesized across the following measures (study count; strength of evidence): duration of abstinence (2; low); quit attempts (15; insufficient); rate of abstinence/quitting (29; moderate); change in smoking quantity/frequency (5; insufficient); and, return to smoking/relapse (2; insufficient). Overall, the qualitative synthesis failed to show a consistent trend for an association between menthol cigarette use and smoking cessation across outcomes. Meta-analyses found no difference between menthol and non-menthol cigarette use and either quit attempts or abstinence.

**Conclusions:**

Given the lack of consistency or statistical significance in the findings—combined with a “low” overall strength of evidence grade, based on deficiencies of indirectness and inconsistency—no consistent or significant associations between menthol cigarette use and smoking cessation were identified. Recommendations for future studies include increased focus on providing longitudinal, adjusted data collected from standardized outcome measures of cessation to better inform long-term smoking cessation and menthol cigarette use. Such improvements should also be further considered in more methodologically rigorous systematic reviews characterized by objectivity, comprehensiveness, and transparency with the ultimate objective of better informing public health and policy decision making.

**Supplementary Information:**

The online version contains supplementary material available at 10.1186/s13011-021-00397-4.

## Background

Currently, the proportion of smokers who use menthol cigarettes is higher among youth than among adults, with about three out of ten adult cigarette smokers choosing to smoke menthol cigarette brands [1]. Based on data from the U.S. Centers for Disease Control [2], rates of adult cigarette smoking have steadily declined over the last half century, from 42% in 1965 to 17% in 2014. Despite this overall decline in smoking, the Substance Abuse and Mental Health Services Administration [3] has noted that menthol cigarette use seems to be characterized by a contradictory upward trend among younger adults, females, males, Hispanics, and Asians. Thus, trends in smoking are inconsistent between menthol and non-menthol cigarette smokers.

In recent years, the potential impact of menthol versus non-menthol cigarette use on smoking behaviors has been an intensely scrutinized topic in the public health arena. More recently, the issue has been brought to the forefront of tobacco policy and decision making, as evidenced by the Food and Drug Administration’s (FDA) recently-declared intent to explore a ban on mentholated tobacco products. Given the FDA’s own commitment to evidenced-based actions [4], there is a clear need for the potential associations between menthol cigarettes and smoking behaviors to be explored scientifically. To date, several narrative reviews have been conducted. However, study methods and the included individual publications have varied, and conclusions have been discordant [5–7]. Some of the discord may reflect the complicated constructs related to smoking behaviors and the varying measurements across studies [8, 9].

A recent meta-analysis by Smith and colleagues [10] concluded that, among Blacks/African Americans in the U.S. (one sample including respondents from Canada), menthol smokers had approximately 12% lower odds of smoking cessation compared to non-menthol smokers. However, the meta-analysis was not based on a full, PRISMA-guided systematic review of the available evidence. A second systematic review by Smith et al. [9] found that both men and women exhibit minimal switching between menthol and non-menthol cigarettes, suggesting that preference is established early in an individual’s smoking trajectory. However, these findings were based on a single included study in the review of smoking initiation, and therefore conclusions are limited in generalizability. Similarly, a systematic review by Villanti et al. [7] reported an association between menthol cigarette smoking and increased initiation among youth, increased dependence especially among youth, and reduced cessation among non-Hispanic Whites and racial and ethnic subgroups. However, the validity of these findings are undermined by the failure to apply an adequate appraisal tool—such AMSTAR 2 [8] which would have identified significant methodological insufficiencies.

Given the methodological deficiencies in the current evidence base, the purpose of our review was to systematically assess the potential association between menthol cigarette use and smoking cessation, with a strict methodological focus to the measures and methods used by the included studies.

Further, given that smoking behaviors can vary across different population subgroups—suggesting that both individual and environmental factors influence smoking [11, 12]—it is essential that factors that influence smoking behaviors be considered to the extent possible based on available data. To this end, this review applied the Socio-Ecological Model created by McLeroy et al. [13] to guide consideration of the interrelationships between individuals and their social (micro-), physical (meso-), and policy (macro-) environments. The socio-ecological model includes three main levels of factors that influence an individual’s smoking behaviors: characteristics of the individual (“micro”); characteristics of the individual’s social environment (“meso”); and characteristics of the systems-level environment in which the individual exists (“macro”). Our review also attempted to quantitatively synthesize the evidence with meta-analyses; to the best of the authors’ knowledge, quantitative synthesis of data from a systematic review has not been previously conducted for this evidence base.

## Methods

### Overview

The methods used for this systematic review followed PRISMA guidelines and were applied to a larger literature search strategy of the association between menthol cigarette use and three smoking behaviors—initiation, cessation, and dependence—of which cessation is the focus of this analysis. Specifically, current results assess the Key Question (KQ), “Does menthol cigarette use have a differential impact on smoking cessation compared to non-menthol cigarette use?” The protocol for this systematic review was registered with the PROSPERO international prospective register of systematic reviews on March 22, 2016 and updated on January 10, 2019. The record is available at: https://www.crd.york.ac.uk/prospero/display_record.php?RecordID=119301.

### Literature search strategy

The literature searches were conducted by an Information Specialist. Search terms were developed using text words related to the associations between menthol cigarette use and cessation of cigarette smoking. The search strategy included using synonyms of search terms, truncation, wild card symbols, Boolean logic, proximity operators, and limits to focus the search towards the most relevant clinical literature (see SUPPLEMENTAL SECTION 1: Literature Search Strategy).

The following online databases were searched for relevant articles published from inception to 14 December 2018 (for the initial literature search) and from 01 January 2018 to 12 June 2020 (for the updated literature search): Cochrane Central Register of Controlled Trials, Cochrane Database of Systematic Reviews, Database of Abstracts of Reviews of Effects, MEDLINE, Embase and PsycInfo.

**Fig. 1:**
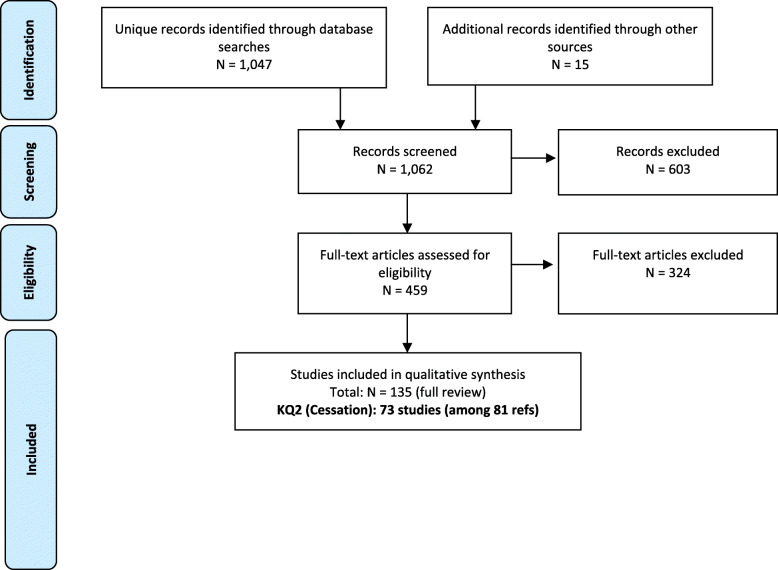
Literature Search Overview

The initial literature search (from inception to 14 December 2018) identified 853 potentially relevant articles, with 838 articles from online databases and 15 additional articles through other sources. An updated literature search (from 01 January 2018 to 12 June 2020) identified an additional 358 potentially relevant articles; however, 149 of the articles were duplicate articles across the two searches, due to a required overlap in the two search timeframes (searches are best conducted from the first of the year). Thus, 209 unique articles were identified in the update literature searching, bringing the total of potentially relevant articles to 1062. After independent review of titles and abstracts by two members of the research team, 603 references were excluded, resulting in 459 articles being screened at the full-text level. An additional 324 articles were excluded at the full-text level (provided in SUPPLEMENTAL SECTION 2: Studies excluded at full-text level screening (with reason for exclusion)), resulting in 135 relevant articles eligible for inclusion; 73 studies (eight of which were reported in paired studies) evaluated the association between menthol cigarette smoking and smoking cessation or cessation-related outcomes (Fig. 1). The weighted overall kappa for inter-rated reliability at full-text screening was 0.96 for the initial literature search, and 0.95 for the updated literature search.

### Eligibility criteria

Eligibility criteria were developed according to the PICO framework and are presented in Table 1. Studies of solely non-U.S. residents were excluded on the basis of variations in national tobacco legislation limiting the generalizability of such studies to the U.S. population.

**Table 1:** Inclusion and Exclusion Criteria for Key Questions (KQs)

	Inclusion Criteria	Exclusion Criteria
**Population**	• Youth and adults • Current or former smokers with explicit use of (or stated preference for) menthol or non-menthol cigarettes; studies of non-smokers were eligible if they reported a measure of initiation • United States residents	• Studies of never-smokers at any time point • Studies of only non-United States residents
**Comparisons**	• Menthol versus non-menthol • Delivery of nicotine via traditional cigarettes	• Studies reporting only menthol or non-menthol or not directly comparing menthol with non-menthol • Studies of non-traditional tobacco delivery^a^
**Outcomes**	• Duration of abstinence; quit attempts (any quit attempts; number of quit attempts per person); rate of abstinence/quitting (including but not limited to prolonged abstinence [PA], point prevalence abstinence [PPA], identifiable cigarette type [menthol versus non-menthol] smoked before quitting, and being a former smoker [versus current smoker]); Change in smoking quantity/frequency; return to smoking/relapse	• Any other outcomes
**Study Designs**^b^	• Randomized and non-randomized controlled trials • Cross-sectional, case-control, and cohort studies • Letters and editorials containing original data not available elsewhere were eligible	• Reviews, case reports, editorials, and letters not containing original data

^a^ E.g., vaporizers, e-cigarettes, hookahs/water pipes

^b^ Reclassification of included trials as cross-sectional or cohort depending on the eligible data as follows: if only baseline data used from a trial, it was considered a cross-sectional study; if any post-baseline measurement data was used, it was considered it a cohort

### Data extraction

Data were extracted and managed through DistillerSR (Evidence Partners, Ottawa, Canada). Articles were initially screened at the title/abstract level; full-text articles were obtained for studies not excluded based on the title/abstract alone. Two reviewers independently screened articles based on the inclusion/exclusion criteria. Any discrepancies between the two were resolved in a joint-reviewer decision. Any unresolved disagreements were adjudicated by a third clinical reviewer; reasons for exclusions were documented.

Data were independently extracted by one research associate and checked by a second research associate. Discrepancies were resolved through discussion and included a third team member when necessary. Data extraction forms were created in DistillerSR.

### Study quality assessment

#### Study quality rating

A random and sufficient sample of included studies was assessed independently by two members of the review team. The level of agreement between those researchers was evaluated based on the mean difference in scores between the two reviewers. The mean difference was 0.25 points (95% CI, − 0.53 to 1.03), indicating that, on average, reviewers had a high level of agreement that the true mean difference was no greater than one point on the scale. The difference in score across studies was distributed normally, suggesting no systematic bias. Based on the high level of agreement, the ratings were not found to be subject to individual reviewer bias, and a single reviewer reviewed the remaining included studies.

#### Downs and Black checklist

The quality of the studies included in this systematic review was assessed at the study level using the Downs and Black checklist [14]. The instrument was used as reported in the original publication, with only one adaptation of the power question as to whether the study was adequately powered (yes/no). The maximum achievable score for a study was 28, and score ranges were grouped into the following four quality levels: “excellent” (26–28); “good “(20-25); “fair” (15–19); and “poor” (≤14). When data from a single study were reported in multiple references, all references were considered to determine an overall rating for the study.

#### Assessment of confounding

A list of potential confounding factors was identified a priori based on evidence and expert opinion from members of the research team and external advisors. Variables that individual study authors considered were recorded for additional post hoc consideration.

This review assessed evidence that adequately controlled for confounding bias according to the predetermined confounders of age, race/ethnicity, and gender. Studies that also adjusted for additional potential *meso*- (e.g., living with a smoker) or *macro*-level factors (e.g., cigarette taxes) were flagged for inclusion in sensitivity analyses. Studies with potential overadjustment or adjustment for factors in the causal pathway were also flagged for further examination in sensitivity analyses.

### Conceptual framework

This review applied the Socio-Ecological Model [13] to guide consideration of the interrelationships between individuals and their social (micro-), physical (meso-), and policy (macro-) environments.

### Outcomes and related psychometrics

Included studies reported on at least one of the following cessation—or cessation-related—outcomes: duration of abstinence, quit attempts (any quit attempts; number of quit attempts per person), rate of abstinence/quitting, change in smoking quantity/frequency, and return to smoking/relapse. Recognizing that not all the outcome measures are likely to be equally valid and reliable, this review examined the following Contextual Question (CQ) to provide additional information and context for the results, “Have measures used to examine cigarette smoking cessation been psychometrically assessed as valid and reliable?” The applied scoring approach was informed by the IARC Handbook of Cancer Prevention [15].

### Data analysis

The strongest evidence to assess whether menthol cigarette use has a differential impact on smoking cessation compared to non-menthol cigarette use would be expected to be provided by longitudinal analyses that adjusted or controlled for key confounding factors – age, race/ethnicity, and gender – by inclusion criteria, modeling, or stratification. Consequently, all studies that controlled for, at minimum, age, gender, and race/ethnicity were qualitatively synthesized.

Longitudinal analytic results were considered the highest available evidence and, as such, were weighed more heavily in the strength of evidence analysis and qualitative synthesis below. In the absence of longitudinal analytic results, the highest level of available evidence was synthesized according to studies that controlled for the predefined demographic factors.

#### Statistical significance

Estimates of the difference between menthol and non-menthol smokers are presented with the best measure of precision (i.e., 95% confidence intervals) or statistical significance (i.e., *p*-value) reported in the included studies. The words “*significant*” and “*significantly*” are used herein to indicate *statistical* significance (i.e., *p* < 0.05 and/or confidence interval excludes 1.0).

#### Meta-analysis

For the meta-analyses, all included studies were controlled, at minimum, for age, gender, race/ethnicity. Menthol cigarette use was defined as either self-reported menthol use, current use, usual cigarette/brand used, or remaining with menthol cigarettes through the length of the study. Subgroup analysis was conducted to compare differences between study designs (prospective cohort and cross-sectional designs in abstinence [no duration]) and differences in measures (past year and ever quit attempt [ever quit attempts, any quit attempts between 2001 and 2005, and any quit attempts in the past 2, 3, or 5 years]). Further, sensitivity analyses were also completed according to race/ethnicity and abstinence verification (eCO verified), when possible. Pooled adjusted odds ratios (AORs) and 95% confidence intervals (CIs) with two-sided *P* values are reported from random-effects models utilizing the DerSimonian and Laird method [16] to measure the likelihood of reporting having made a quit attempt and abstaining among menthol compared to non-menthol smokers. Variations among pooled studies were assessed using Cochran’s Q statistic and heterogeneity was quantified using the inconsistency index (I^2^). A *p* value less than 0.10 was considered significant. I^2^ expresses the percent of variability in point estimates due to heterogeneity and results here follow the categories of low (I^2^ = 25%), moderate (I^2^ = 50%), and high (I^2^ = 75%) [17]. All data were analyzed through Review Manager version 5.3 [18].

## Strength of evidence evaluation

Recognizing the inherent limitations when assessing confidence in empirical conclusions based on observational data [19–22], the Agency for Healthcare Research and Quality (AHRQ) Evidence-Based Practice Center (EPC) approach – based largely on the methods developed by the Grading of Recommendations Assessment, Development and Evaluation (GRADE) Working Group [23] – was deemed acceptable for this review. Strength of evidence for this review was evaluated based on the four required domains:

1. *Study limitations (previously called risk of bias)* – The degree to which included studies for a given outcome have a high likelihood of adequate protection against bias (ie, good internal validity), assessed through two main elements, study design and study conduct.
2. *Directness* – Whether evidence links interventions directly to a health outcome of specific importance for the review and, for comparative studies, whether the results are based on head-to-head comparisons.
3. *Consistency* – The degree to which included studies find either the same direction or similar magnitude of effect, as assessed by direction of effect and/or magnitude of effect.
4. *Precision* – The degree of certainty surrounding an effect estimate with respect to a given outcome, based on the sufficiency of sample size and number of events.

Reporting bias is one of the strength of evidence (SOE) domains typically assessed for systematic reviews, but the methods used to detect such bias are designed for use with controlled trials. Although observational studies may be susceptible to reporting bias, no comparable methods exist for assessing reporting bias for these study designs. As a result, reporting bias was not assessed for the purposes of this systematic review, which comprised of only observational studies, in accordance with methodological recommendations [24].

For this review, the SOE was assessed in two ways for each outcome measure. First, SOE was assessed for the studies that adjusted for the key confounders of age, race/ethnicity, and gender (through multivariable modeling, sample stratifications, or predefined study inclusion criteria). These results minimized the potential for confounding bias, represented the “best evidence,” and thus may be more likely to represent the “true” association between menthol cigarette use and smoking behaviors.

Next, a sensitivity analysis was conducted to include the results from analyses that did not control for the key confounders. The unadjusted results reflected the effect of menthol cigarette use but allow all other variables—measured and unmeasured—to vary, potentially obscuring the actual effect of menthol smoking.

In both SOE assessments, measures with “acceptable” reliability and/or validity were weighed more heavily than the “inconclusive” measures (to minimize the impact of misclassification bias).

The final SOE judgment was necessarily qualitative but reflected a sound, reasoned weighing of domain ratings.

The overall strength of the body of evidence was graded as “high,” “moderate,” “low,” or “insufficient” using the Evidence-Based Practice Center (EPC) approach (Table 2).

**Table 2:** Strength of Evidence Grades and Definitions

Grade	Interpretation	Description
**High**	Very confident that the estimate of effect lies close to the true effect for this outcome.	^•^ The body of evidence has few or no deficiencies. ^•^ We believe that the findings are stable, that is, another study would not change the conclusions.
**Moderate**	Moderately confident that the estimate of effect lies close to the true effect for this outcome.	^•^ The body of evidence has some deficiencies. ^•^ We believe that the findings are likely to be stable, but some doubt remains.
**Low**	Limited confidence that the estimate of effect lies close to the true effect for this outcome.	^•^ The body of evidence has major or numerous deficiencies (or both). ^•^ We believe that additional evidence is needed before concluding either that the findings are stable or that the estimate of effect is close to the true effect.
**Insufficient**	No evidence; unable to estimate an effect, or no confidence in the estimate of effect for this outcome.	^•^ No evidence is available or the body of evidence has unacceptable deficiencies, precluding reaching a conclusion.

### Sensitivity analysis

Additionally, three sensitivity analyses were conducted in order to evaluate the SOE, to include: limitation of the study pool to those that also adjusted for meso- and/or macro-level variables; exclusions of “poor” quality studies (according to the Downs and Black study quality assessment); and exclusion of studies with potential overadjustment and/or inappropriate adjustment.

## Results

A total of 73 studies, reported in 81 unique references, evaluated the potential associations between menthol cigarette use and smoking cessation. Adjusted studies were considered a higher level of evidence and, therefore, all subsequent analyses were restricted to studies that adjusted for key demographic characteristics. A total of 43 studies, reported in 47 unique references, provided adjusted data for relevant smoking cessation outcomes; complete study characteristics are shown in Table 3. The definitions of the specific outcome measures for smoking cessation applied across the adjusted studies are presented in SUPPLEMENTAL SECTION 3: Outcome Measures for Smoking Cessation across Adjusted Studies.

**Table 3:** Study, Data Set, and Sample Characteristics

First Author, Year National survey name Quality rating	Location; Funding; Type of study	Sampling / recruitment strategy; Data collection period	Brief summary of inclusion criteria (including definition of “smoker”)	Special population
Alexander et al., 2010 [25] TUS-CPS Good	National; NR; Cross-sectional	Stratified multistage probability sample, civilian non-institutionalized U.S. population; 2006–2007	Adult current smokers age ≥ 18 (smoke every day or some days).	None
Azagba et al., 2019 [26] NYTS Fair	National; NR; Cross-sectional	A nationally representative sample of students enrolled in grades 6 through 12. The sampling universe consists of public and private school students in the 50 states and the District of Columbia. Primary sampling units are selected with probability proportional to the student enrollment in the PSU but giving disproportionate weight to Black, Asian, and Hispanic students. All students present in a selected classroom on the day of the interview are selected for the study; 2017–2018	Middle school and high school students who were current cigarette users, defined as smoking at least one out of the past 30 days. Smoking frequency was derived from the question “During the past 30 days, on how many days did you smoke cigarettes?” with the following possible answers: “0 days,” “1 or 2 days,” “3–5 days,” “6–9 days,” “10–19 days,” “20–29 days,” and “All 30 days.”	Middle school (grades 6 to 8) and high school (grades 9 to 12)
Blot et al., 2011 [27] None Fair	Southern states (Alabama, Arkansas, Florida, Georgia, Kentucky, Louisiana, Mississippi, North Carolina, South Carolina, Tennessee, Virginia, West Virginia); Govt; Prospective cohort	Southern Community Cohort Study (SCCS), residents from southern U.S. states recruited from mailings to age, gender, and race-stratified random samples of the general population, predominately (~ 85%) at community health centers; March 2002 – September 2009	Adults age 40–79 living in U.S. southern states. Ever-smokers (≥100 lifetime cigs), continuing smokers (current smokers at baseline who reported smoking in the follow-up questionnaire), and former smokers at baseline. Respondents to the follow-up survey tended to be women, older, and of higher income and education level than non-respondents.	Age 40–79 living in U.S. southern states
Cropsey et al., 2009 [28] None Fair	Virginia; Govt; Prospective cohort	Recruited through announcements and study flyers in prison housing units at a medium-maximum security female prison; June 2004–June 2006	Adult women age ≥ 18 who smoke ≥5 CPD, are not held in segregation from other prisoners and desired smoking cessation treatment.	Female prisoners
Cubbin et al., 2010 [29]NHIS-CCS Good	National; NR; Cross-sectional	Administered in 1992, 2000, 2005, and 2010 as a supplement to the NHIS that assesses issues related to cancer-related behaviors, screening, and risk assessment, including tobacco use and control; 2005	Adults age 25–64 who self-identified as Black, non-Hispanic/Latino, Hispanic/Latino, or White non-Hispanic, smoked ≥100 lifetime cigs and currently smoke every day (current smoker) or do not currently smoke (former smoker).	None
D’Silva et al., 2012 [30] None Fair	Minnesota; Govt; Prospective cohort	People who called the ClearWay Minnesota line; September 2009 – July 2011, 7-month post-registration follow-up survey March 2010–February 2011	Adult smokers who registered for cessation counseling services.	None
Delnevo et al., 2010; Delnevo et al., 2011 [31, 32], 2010 TUS-CPS Good	National; Govt; Cross-sectional	Stratified multistage probability sample, civilian non-institutionalized U.S. population; 2003, 2006–2007	White, Black and Hispanic ever-smokers (≥100 lifetime cigs) age ≥ 18 who were current smokers (smoking “everyday” or “some days”) or former smokers (quit in the past 5 years) at the time of survey.	None
Fagan et al., 2007 [33] TUS-CPS Fair	National; Govt; Cross-sectional	Stratified multistage probability sample, civilian non-institutionalized U.S. population; 2003	Young adult (18–30 years) current smokers who smoke every day (daily smokers) or some days (non-daily smokers).	Young adults age 18–30
Faseru et al., 2013 [34] None Good	Kansas; Govt; Prospective cohort	Kick it at Swope-III (KIS-III trial), recruited at a community-based clinic serving a predominantly Black population; NR	Black adult (≥18 years) “light smokers” (≤10 CPD) for ≥2 years who smoked on ≥25 days in the month prior to enrollment and were interested in quitting.	Black light smokers (≤10 CPD)
Foulds et al., 2006 [35] None Good	New Jersey; Govt & foundation; Prospective cohort	Convenience sample of patients attempting to quit at a specialist tobacco dependence treatment outpatient clinic; 2001–2006	Smokers age 14–81 who, at their assessment, reported current smoking, specified a target quit date, and responded to the baseline night-smoking question.	None, but included age ≥ 14
Fu et al., 2008 [36] None Good	5 VA centers in U.S.; Govt; Prospective cohort	Participants recruited from 5 VA medical centers, identified by VA pharmacy databases; February–October 2002	Adult smokers age ≥ 19 with a recent quit attempt that incorporated pharmacologic treatment.	VA patients
Gandhi et al., 2009 [37] None Fair	New Jersey; Govt & foundation; Retrospective cohort	Consecutive patients at a specialist tobacco treatment outpatient clinic; January 2001–June 2005	Current smokers age 15–80 who set a quit date and attempted to quit smoking.	None, but included age ≥ 15
Gubner et al., 2018 [38] None Good	National (USA); National Institute on Drug Abuse (National Institutes of Health), Food and Drug Administration Center for Tobacco Products; Cross-sectional	Convenience sampling from each of 24 substance use disorder treatment centers (in the National Institute on Drug Abuse Clinical Trials Network), with self-administered surveys conducted during on-site visits; April to December 2015	Individuals with substance use disorders who self-reported as current smokers.	Individuals with substance use disorders
Gundersen et al., 2009 [39] NHIS-CCS Good	National; NR; Cross-sectional	Administered in 1992, 2000, 2005, and 2010 as a supplement to the NHIS that assesses issues related to cancer-related behaviors, screening, and risk assessment, including tobacco use and control; 2005	Adult White, Black and Hispanic cig smokers (≥100 lifetime cigs) age ≥ 18 who ever attempted to quit smoking, do not currently use other tobacco products, and were current smokers (currently smoking “everyday” or “some days”) or former smokers (currently smoke “not at all”).	None
Hyland & Rivard, 2010; Hyland et al., 2002 [40, 41] None Good	2 sites in each of: New Jersey, California, New Mexico, New York, North Carolina, Canada; Govt; Prospective cohort	COMMIT cessation trial; modified random-digit-dial method and geographic boundary screening used for the baseline prevalence survey to obtain representative samples of approximately 5400 HHs. Focused on communities with the highest prevalence of non-Whites; 1988–1993	Adult current smokers age 25–64 who reported whether their current brand was mentholated or not in 1988, and had a known smoking status in 1993.	None
Kahende et al., 2011 [42] TUS-CPS Fair	National; NR; Cross-sectional	Stratified multistage probability sample, civilian non-institutionalized U.S. population; 2003, 2006–2007	Adults age ≥ 18 who smoked cigs during the past year.	None
Kasza et al., 2014 [43] ITC-4 (U.S. data only) Fair	National; Foundation & Govt; Prospective cohort	Random digit dialing to recruit adult smokers from the United States, Canada, the United Kingdom, and Australia. Eight waves have been completed between 2002 and 2011. Only U.S. data are included in this Review; 2002–2011	Adult smokers (≥100 lifetime cigs and smoked ≥once in past 30 days).	None
Keeler et al., 2017 [44] TUS-CPS Fair	National (USA); Tobacco Related Disease Research Program; Cross-sectional	Stratified multistage probability sample, civilian non-institutionalized U.S. population; 2006 to 2007 and 2010 to 2011	Adult recent active smokers age ≥ 18, defined as current smokers or former smokers who quit less than 12 months ago; current smokers defined as smoking 100 cigarettes in their lifetime and currently smoking every day (daily smokers) or some days (someday smokers); former smokers defined as individuals who smoked 100 cigarettes in their lifetime but currently do not smoke	None
Keeler et al., 2018 [45] TUS-CPS Fair	National (USA); Tobacco Related Disease Research Program; Cross-sectional	Stratified multistage probability sample, civilian non-institutionalized U.S. population; May/August 2006 to January 2007, and May/August 2010 to January 2011	Adult recent active smokers age ≥ 18, to include current and former smokers who quit less than 12 months ago; current smokers, defined as having smoked 100 cigarettes in their lifetime and were currently smoking cigarettes every day or some days; former smokers defined as having smoked 100 cigarettes in their lifetime who currently did not smoke.	Subsamples of African-American and White respondents, respectively.
Levy et al., 2011 [46] TUS-CPS Good	National; Foundation; Cross-sectional	Stratified multistage probability sample, civilian non-institutionalized U.S. population; 2003, 2006–2007	Adults age ≥ 18 who smoked ≥100 lifetime cigs and were currently smoking or quit between 3 months and 5 years prior to the interview.	None
Lewis et al., 2014 [47] None Fair	National; Govt; Prospective cohort	Nielsen Homescan Panel which provides a record of consumer-packaged goods purchased by a large panel of nationally representative U.S. HHs; January 2004–December 2009	Cig purchasers (made ≥1 cig purchase in 2004 and in 2005 or later and purchased ≥20 packs between 2004 and 2009) who resided in 1 of the top 75 Designated Market Areas in order to track anti-smoking advertising. Homeowners were age ≥ 18.	None
Muench and Juliano, 2017 [48] None Good	Washington, DC metropolitan area; United States Department of Health and Human Services, National Institutes of Health, National Institute on Drug Abuse, and the College of Arts and Sciences at American University; Laboratory-based smoking-choice study	Sample was recruited through flyers, word of mouth, and online advertisements, and participants were screened for eligibility by phone; NR	Adults age ≥ 18 who smoked a ≥ 10 CPD for at least the past year, and with no intention or current attempt of quitting.	None
Muscat et al., 2002 [49] None Fair	New York, District of Columbia, Pennsylvania; Govt; Case-control	Newly diagnosed cancer patients were identified from thoracic and other surgery schedules. Non-surgical patients recruited from oncology wards. Controls were selected randomly from general hospital admitting rosters; 1981–1999	Black or White current smokers (≥1 CPD for the past year) and former smokers (current smokers at one time but did not smoke ≥1 CPD for the preceding year).	Cancer patients (and non-cancer control)
Nonnemaker et al., 2012 [50] None Good	7 communities in 5 states; Govt; Prospective cohort	American Legacy Longitudinal Tobacco Use Reduction Study (ALLTURS): School-based survey of middle and high school youth conducted in three waves in 83 schools in 7 communities in 5 states, initially selected for a quasi-experiment that included matched communities; 2000–2002	Youth age < 18 years who initiated smoking after baseline and before wave 3 and completed all 3 annual waves of the study.	Youth
Okuyemi et al., 2003 [51] None Good	Kansas; Govt; Prospective cohort	Kick it at Swope trial (KIS), inner-city health center mostly serving a low-income Black population. Patients were invited to participate in a study on smoking among inner-city residents, not associated with a cessation program; August 2000–November 2000.	Adult current smokers (≥10 CPD) age ≥ 18 who were Black, spoke English, had a home address with a working telephone, and were interested in quitting in the next 30 days.	Blacks
Okuyemi et al., 2007 [52] NoneGood	Kansas; Govt; Prospective cohort	Kick it at Swope trial (KIS), inner-city health center mostly serving a low-income Black population. Patients were invited to participate in a study on smoking among inner-city residents, not associated with a cessation program; March 2003–June 2004	Black adult (≥18 years) light smokers (≤10 CPD for ≥6 months and smoking on ≥25 of last 30 days) who were interested in setting a quit date within 14 days.	Black light smokers (< 10 CPD)
Park, 2017 [53] NATS Good	National (USA); NR; Cross-sectional	Stratified, national, landline and cell phone survey of non-institutionalized adults age ≥ 18 across the 50 states and the District of Columbia; NATS 2012–2013 used a dual frame random digit dialing sample, drawn from landline and cell phone frames; October 2012 to July 2013	Adult current smokers age ≥ 18, defined as having smoked least 100 cigarettes in their lifetime and currently smoked cigarettes every day or some days.	None
Pletcher et al., 2006 [54] None Good	A major city in each of: Alabama, Illinois, Minnesota, California; Govt; Prospective cohort	Coronary Artery Risk Development in Young Adults Study (CARDIA), population-based observational study of men and women age 18–30 at baseline with follow-up data through year 19. Selected for equal representation in subgroups of race, gender, education, and age, recruited across 4 U.S. study sites; 1985–2000	Young adult (18–30 years) Black or European-American current smokers.	Young adults age 18–30 at baseline
Rath et al., 2015 [55] LYAC Fair	National; Govt; Prospective cohort	GfK’s KnowledgePanel - an online panel of adults age 18 or older that covers both the online and offline populations in the U.S. Recruited via address-based sampling, a probability-based sampling method that provides statistically valid representation of the U.S. population, including cell-phone only households. Black and Hispanic young adults were oversampled; 2011–2012	Adults age 18–34 who completed the first 3 surveys and either remained current smokers across all 3 time points or initiated cig smoking at Time 2 and remained cig smokers at Time 3. Current smokers used ≥1cigs in the past 30 days.	Young adults
Reitzel, 2011a [56] None Good	Texas; Govt; Prospective cohort	Project BREAK FREE, recruited from within the Houston metro area through local print and radio advertisements; 2005–2007	Adult Blacks who smoked ≥5 CPD for ≥12 months, had eCO ≥8 ppm, were willing to quit smoking in the next 2 weeks, had a working home telephone and a permanent address, and a 6th grade English literacy level.	Blacks
Reitzel, 2011b [57] None Good	Texas; Govt; Prospective cohort	Project CARE recruited from within the Houston metro area through local print and radio advertisements; 2005–2007	Adult current smokers (≥5 CPD for the past year) age ≥ 21.	None
Reitzel, 2011c; Reitzel et al., 2011 [58, 59] None Good	Texas; Govt; Prospective cohort	Project MOM, recruited from within Houston metropolitan area through a local health care system and via newspaper, radio, bus, and clinic advertisements; 2005–2007	Adult women in week 30–33 of pregnancy at time of enrollment. Smokers (≥1 CPD on average for the year) stopped smoking either during their pregnancy or within 2 months prior to becoming pregnant and wanted to remain quit postpartum. Women reporting a high-risk pregnancy were excluded.	Pregnant women
Reitzel et al., 2013 [60] None Fair	Texas; Govt; Prospective cohort	Longitudinal study among community smokers; 2006–2007	Adult smokers age 18–65 (≥5 CPD for ≥12 months) who had a working telephone number, permanent home address, and 6th grade literacy level. Willing to quit smoking in the next week and did not participate in a smoking cessation program in the last 3 months.	None
Rojewski et al., 2014 [61] None Good	Connecticut; Govt; Prospective cohort	Media and provider referrals; 2005–2009	Weight-concerned smokers (> 10 CPD for ≥1 year) with eCO ≥10 ppm, ≥1 prior quit attempt, and were enrolled in a cessation trial.	None
Sawdey et al., 2020 [62] NYTS Good	National; None; Cross-sectional	A nationally representative sample of students enrolled in grades 6 through 12. The sampling universe consists of public and private school students in the 50 states and the District of Columbia. Primary sampling units are selected with probability proportional to the student enrollment in the PSU but giving disproportionate weight to Black, Asian, and Hispanic students. All students present in a selected classroom on the day of the interview are selected for the study; 2011–2018	Youth (grades 6–12) current smokers that reported smoking a cigarette ≥1 day in the past 30 days	Middle and high school students (grades 6–12)
Schneller et al., 2020; Schneller, 2020 [63, 64] PATH Fair	National; PATH contract mechanism; Cross-sectional	Nationally-representative survey of civilian, non-institutionalized US citizens, using addressed-based, probability sampling of households with adolescents. Data gathered in waves, beginning with Wave 1 (September 2013–December 2014) and having currently completed Wave 5 (2016–2017; data collection planned through 2024); 12 September 2013 to 14 December 2014 (Wave 1), 23 October 2014 to 30 October 2015 (Wave 2)	Current adult cigarette smokers that have smoked at least 100 cigarettes in their lifetime and smoke every day or somedays	None
Stahre et al., 2010 [65] NHIS-CCS Fair	National; Govt; Cross-sectional	Administered in 1992, 2000, 2005, and 2010 as a supplement to the NHIS that assesses issues related to cancer-related behaviors, screening, and risk assessment, including tobacco use and control; 2005	Adult current and former smokers age ≥ 18 for whom menthol status was known.	None
Steinberg et al., 2011 [66] None Good	New Jersey; None; Retrospective cohort	Smokers intending to quit enrolled in a study for smoking cessation; 2006–2008	Adult smokers age 16–78 presenting for cessation treatment.	None
Sulsky et al., 2014 [67] NHIS, TUS-CPS Good	National; Industry; Cross-sectional	Stratified multistage probability sample, civilian non-institutionalized U.S. population; 2005, 2010 TUS-CPS; 2010–2011	Adult current smokers (≥100 lifetime cigs) and former smokers (≥100 lifetime cigs who quit ≥1 year before survey).	None
Thihalolipavan et al., 2014 [68] None Poor	New York; Govt; Prospective cohort	New York City Nicotine Patch and Gum Program (NPGP), a nicotine replacement therapy giveaway administered to smokers who phoned a toll-free quitline; 2012	Adult daily smokers in a nicotine replacement therapy giveaway.	None
Trinidad et al., 2010 [69] TUS-CPS Good	National; Govt & foundation; Cross-sectional	Stratified multistage probability sample, civilian non-institutionalized U.S. population; 2003, 2006–2007	Adult ever smokers age 20–65, including current smokers (≥100 lifetime cigs and currently smoke every day or some days) and former smokers (≥100 lifetime cigs and currently smoke not at all).	None
Webb Hooper et al., 2011 [70] BRFSS Good	Florida; Govt; Cross-sectional	BRFSS subsample from Florida; April 2007 – January 2008	Adult smokers age ≥ 18 (≥100 lifetime cigs and currently smoking on some days).	None
Winhusen et al., 2013 [71] None Fair	National; Govt; Prospective cohort	Randomized trial examining substance use disorder treatment with smoking cessation treatment. Participants recruited from one of 12 nationwide outpatient treatment programs; Feb 2010-July 2012	Adult current smokers (≥7 CPD and eCO ≥8 ppm) enrolled in outpatient treatment for cocaine or methamphetamine addiction and are interested in smoking.	Cocaine- or methamphetamine-dependent smokers.

*Abbreviations*: *ALLTURS* American Legacy Longitudinal Tobacco Use Reduction Study, *BRFSS* Behavioral Risk Factor Surveillance System, *CARDIA* Coronary Artery Risk Development in Young Adults Study, *eCO* exhaled carbon monoxide, *COMMIT* Community Intervention Trial for Smoking Cessation, *CPD* cigarettes per day, *cigs* cigarettes, *Govt* government, *HH* household, *ITC-4* International Tobacco Control Four Country Survey (U.S. data only), *KIS-III* Kick it at Swope III Trial, *LYAC* Legacy Young Adult Cohort, *NATS* National Adult Tobacco Survey, *NHIS* National Health Interview Survey, *NHIS-CCS* National Health Interview Survey Cancer Control Supplement, *NPGP* New York City Nicotine Patch and Gum Program, *NR* not reported, *NYTS* National Youth Tobacco Survey, *ppm* parts per million, *SCCS* Southern Community Cohort Study, *TUS-CPS* Tobacco Use Supplement to the Current Population Survey, *VA* Veterans Health Administration

Table 4 contains a summary of the identified published assessments of the psychometric foundations for the smoking cessation measures. Empirical data regarding reliability or validity qualified four of the five smoking cessation measures (duration of abstinence, quit attempts, rate of abstinence/quitting, change in smoking quantity/frequency) as “acceptable”.

**Table 4:** Overview of Psychometric Findings for Measures of Smoking Cessation

	Statistical reliability indicator ≥ 0.70?			Statistical Validity Indicator?			External (Population) Validity?			Overall Psychometric Rating
	Both Genders	By Age		[Criterion, Predictive, Convergent]			Both Genders	By Age		
Scale or Measure		Adults	Youth	Behavioral	Bio-chemical	Cessation		Adults	Youth	
Duration of abstinence	Yes [9, 72, 73]	N/A	N/A	Yes [74]	No	N/A	Yes [9]	N/A	N/A	A
Quit attempts										
Any quit attempts	Yes [75, 76]	N/A	N/A	No	No	N/A	No	N/A	N/A	A
Number of quit attempts per person	Yes [73, 76]	N/A	N/A	Yes [76]	No	N/A	No	N/A	N/A	
Rate of abstinence/quitting										
Point/period prevalence abstinence (PPA)	Yes [77–79]	N/A	N/A	No	Yes [80–87]	N/A	No	N/A	N/A	A
Prolonged abstinence (PA)	No	N/A	N/A	Yes [85–89]	No	N/A	Yes	N/A	N/A	
Former smoker vs. current smoker	No	N/A	N/A	No	No	N/A	No	N/A	N/A	
Menthol cigarette use prior to quitting	No	N/A	N/A	No	No	N/A	No	N/A	N/A	
Change in smoking quantity/frequency	Yes [72–76, 79, 86, 90–95]	N/A	N/A	Yes [74, 79, 80, 92, 93, 96]	Yes [79, 80, 93, 96]	N/A	Yes [79, 80]	N/A	N/A	A
Return to smoking/relapse	Yes [73]	N/A	N/A	No	No	N/A	No	N/A	N/A	I

### Synthesis of the best available evidence

Summaries of the best available evidence — controlling for age, race/ethnicity, and gender — are presented by outcome measure below. Outcome measures are presented with a corresponding overview table for each measure in the following order: duration of abstinence; quit attempts; rate of abstinence/quitting; change in smoking quantity/frequency; and return to smoking/relapse. Where two references reported the same data, the most recent publication was used as the data source. The complete data extraction for all included adjusted studies can be found in SUPPLEMENTAL SECTION 4: Evidence Table, Modeled / Adjusted Results.

#### Duration of abstinence

Two studies, presented in Table 5, reported duration of abstinence.

**Table 5:** Summary of Evidence Related to Duration of Abstinence

Study	Sampling / Recruitment Strategy^**a**^, Data Collection Period	Study Findings	Study Quality
Decreased Duration of Abstinence with Menthol Cigarette Use			
Levy et al., 2011 [46]	TUS-CPS, 2003,2006/2007	Menthol cigarette use was associated with significantly lower odds of being a “recent” quitter (those who quit in the past year and had been abstinent for at least 3 months; AOR = 0.97, 95% CI: 0.96 to 0.97; *p* < 0.001) and a “long-term” quitter (those who quit in the past 5 years and had been abstinent for at least 3 months; AOR = 0.94; 95% CI: 0.94 to 0.94; *p* < 0.001), compared with use of non-menthol cigarettes. Further controlling for nicotine dependence resulted in nearly identical odds ratios for being a “recent” quitter (AOR = 0.97, 95% CI: 0.96 to 0.97; *p* < 0.001) and a “long-term” quitter (AOR = 0.95; 95% CI: 0.95 to 0.95; *p* < 0.001). A third adjusted model detected similar odds ratios for “recent” quitters (AOR = 0.92, 95% CI: 0.91 to 0.92; *p* < 0.001) and “long-term” quitters (AOR = 0.95; 95% CI: 0.95 to 0.95; *p* < 0.001).	Good
Results of Mixed Significance for Duration of Abstinence			
Cubbin et al., 2010 [29]	NHIS-CCS; 2005	Increase in Duration of Abstinence with Menthol Cigarette Use Among the six gender-race/ethnicity interactions, White female former menthol smokers reported significantly longer abstinence than White female former non-menthol smokers (14.8 years vs. 12.5 years, respectively; *p* < 0.01). No Difference For the other interactions (White males, Black females, Black males, Hispanic females, and Hispanic males), no difference was found.	Good

^a^ Details of sampling and recruitment strategies for the data sources can be found in Table 3: Study, Data Set, and Sample Characteristics

Levy at al [46]. reported significantly lower odds of being a “recent” and “long-term” quitter for menthol compared with non-menthol smoking, across all models (AORs ranged from 0.92 to 0.97 across models). Cubbin et al. [29] reported duration of abstinence for six gender-race/ethnicity interactions, yielding only one significant finding that suggested White female menthol smokers had been abstinent significantly longer than White female non-menthol smokers (14.8 years vs. 12.5 years; *p* < 0.01). Given the limited number of studies and the inconsistent findings reported for this measure, an association between menthol cigarette use and duration of abstinence is unclear and undefined in the evidence base.

#### Quit attempts (any quit attempts; number of quit attempts per person)

Fifteen studies (from 16 references), as presented in Table 6, reported measures of quit attempts.

**Table 6:** Summary of Evidence Related to Quit Attempts

Study	Sampling / Recruitment Strategy^**a**^, Data Collection Period	Study Findings	Study Quality
Decreased Quit Attempts with Menthol			
Kahende et al., 2011 [42]	TUS-CPS; 2003, 2006/2007	White menthol smokers had significantly lower odds of having made a quit attempt in the past year (AOR = 0.91, 95% CI: 0.84 to 0.99; *p* < 0.05).	Fair
No Difference in Quits Attempt with Menthol Cigarette Use			
Kasza et al., 2014 [43]	ITC-4, 2002–2011	No difference between smokers who switched from menthol to non-menthol cigarettes compared to smokers who continued smoking menthol cigarettes in quit attempts during (AOR = 1.09, 95% CI: 0.78 to 1.52) or after (AOR = 1.03, 95% CI: 0.66 to 1.60) the switch. Switchers from non-menthol to menthol cigarettes were also no different from smokers who attempted to quit but continued with non-menthol cigarettes during (AOR = 1.12, 95% CI: 0.80 to 1.57) or after (AOR = 0.91, 95% CI: 0.57 to 1.44) the switch.	Fair
Park, 2017 [53]	Dual frame random-digit dialing sample; October 2012 to July 2013	No difference between menthol and non-menthol smokers in the likelihood of a past-year quit attempt (AOR = 1.19, 95% CI: 0.97 to1.46; *p* = 0.92).	Good
Rath et al., 2015 [55]	LYAC; 2011–2012	No difference between menthol and non-menthol smokers in ever having made a quit attempt (AOR = 0.84, 95% CI: 0.43 to 1.63) or having made a quit attempt in the past 6 months (AOR = 0.62, 95% CI: 0.30 to 1.27).	Fair
Webb Hooper et al., 2011 [70]	BRFSS subsample from Florida; April 2007 – January 2008	No difference between menthol and non-menthol smokers in past year quit attempts (AOR = 0.96, 95% CI: 0.81 to 1.15).	Good
Alexander et al., 2010 [25]	TUS-CPS, 2006–2007	No difference between menthol and non-menthol smokers in the odds of making a quit attempt (AOR = 0.98, 95% CI: 0.83 to 1.15).	Good
Cubbin et al., 2010 [29]	NHIS-CCS, 2005	No differences between menthol and non-menthol smokers across all six gender-race/ethnicity interactions in predicted past year quit attempts.	Good
Hyland & Rivard, 2010 [41]	COMMIT cessation trial; 1988–1993	No differences between menthol and non-menthol smokers in the odds of having made a quit attempt (AOR = 0.91, 95% CI: 0.72 to 1.15); similarly, no differences were found when analyzing subgroups of Black and White smokers.	Good
Stahre et al., 2010 [65]	NHIS-CCS, 2005	No differences between menthol and non-menthol current smokers (AOR = 1.05, 95% CI: 0.80 to 1.36) or former smokers (AOR = 1.29, 95% CI: 0.74 to 2.26) in using any type of quit aid.	Fair
Fagan et al., 2007 [33]	TUS-CPS; young adults age 18 to 30; 2003	No differences between menthol and non-menthol current smokers (AOR = 1.00, 95% CI: 0.89 to 1.16), current daily smokers (AOR = 1.00, 95% CI: 0.85 to 1.18), or non-daily smokers (AOR = 0.93, 95% CI: 0.62 to 1.41) in the odds of past-year quit attempts. Moreover, no difference was found in the odds of past-year quit attempts between menthol and non-menthol non-daily smokers who reported an intention to quit (AOR = 1.35, 95% CI: 0.60 to 3.03).	Fair
Pletcher et al., 2006 [54]	CARDIA; men and women age 18–30 at baseline; 1985–2000	Adjusting for various factors in 3 models, results across all models were similar in direction, significance, and magnitude and found no difference between menthol and non-menthol smokers in the likelihood of having made a quit attempt in the period preceding each interview (AOR = 0.77, 95% CI: 0.56 to 1.06; most restrictive model, adjusting for age, race/ethnicity, gender, social factors, and CPD at baseline).	Good
Schneller et al., 2020 [63]; Schneller, 2020 [64]	PATH; 12 September 2013 to 14 December 2014 (Wave 1), 23 October 2014 to 30 October 2015 (Wave 2)	No significant difference in the adjusted risk of menthol users reporting a past 12 month quit attempt compared to non-menthol users (RRR = 1.00, 95% CI: 0.89–1.13, p = NS).	Fair
Results of Mixed Significance in Quit Attempts			
Keeler et al., 2018 [45]	Probability sampling of stratified clusters of U.S. households; May/August 2006 to January 2007, and May/August 2010 to January 2011	Increase with Menthol Cigarette Use Black menthol, compared to non-menthol, smokers were significantly more likely to report any past-year quit attempts (AOR = 1.39, 95% CI: 1.16 to 1.67; *p* < 0.001). No difference No difference between White menthol and non-menthol smokers in the odds of past-year quit attempts (AOR = 0.95, 95% CI: 0.89 to 1.01; p = NS).	Fair
Keeler et al., 2017 [44]	Probability sampling of stratified clusters of U.S. households; 2006 to 2007 and 2010 to 2011	Increase with Menthol Cigarette Use Black menthol smokers were significantly more likely to report past-year quit attempts than non-menthol smokers (AOR = 1.37, 95% CI: 1.16 to 1.61; *p* = 0.0002). No difference No difference between menthol and non-menthol smokers in past-year quit attempts (AOR = 0.99 95% CI: 0.94 to 1.04; *p* = 0.6690). Similarly, no difference in the odds of past-year quit attempts between: White menthol and non-menthol smokers (AOR = 0.97, 95% CI: 0.91 to 1.02; *p* = 0.2450); Asian menthol and non-menthol smokers (AOR = 0.91, 95% CI: 0.62 to 1.34; *p* = 0.6470); or Hispanic menthol and non-menthol smokers (AOR = 1.09, 95% CI: 0.91 to 1.30; *p* = 0.3540).	Fair
Levy et al., 2011 [46]	TUS-CPS; current smokers and former smokers who quit between 3 months and 5 years prior to the survey interview; 2003, 2006/2007	Increase with Menthol Cigarette Use Menthol, versus non-menthol, smokers who were smoking 1 year prior to the interview had a significantly higher likelihood of past year quit attempts (AOR = 1.03, 95% CI: 1.02 to 1.03; *p* < 0.001). Further controlling for nicotine dependence resulted in a nearly identical and significantly higher likelihood of a past year quit attempt for menthol, versus non-menthol, smokers who were smoking 1 year prior to the interview (AOR = 1.02, 95% CI: 1.02 to 1.03; *p* < 0.001). Decrease with Menthol Cigarette Use A third adjusted model reported significantly lower odds of past-year quit attempts for menthol, versus non-menthol, smokers (AOR = 0.98, 95% CI: 0.98 to 0.98).	Good

^a^ Details of sampling and recruitment strategies for the data sources can be found in Table 3: Study, Data Set, and Sample Characteristics

Kahende at al [42]. reported White menthol smokers had significantly lower odds than White non-menthol smokers of having made a past-year quit attempt (AOR = 0.91, 95% CI: 0.84 to 0.99; *p* < 0.05).

Ten studies (from 11 references) found no difference between menthol and non-menthol smokers in terms of having made at least one quit attempt (within various timeframes), across all models and subgroup analyses/stratifications performed [25, 29, 33, 41, 43, 53–55, 63, 64, 70]. In addition, Stahre et al. [65] found no significant difference in the odds of using any type of quit aid between menthol and non-menthol current smokers, nor menthol and non-menthol former smokers.

Three studies reported mixed findings. Levy et al. [46] reported that menthol cigarette smokers had significantly higher odds of past-year quit attempts compared to non-menthol users (AOR = 1.03, 95% CI: 1.02 to 1.03; *p* < 0.001); this result remained unchanged when adding nicotine dependence to the model. However, a third model (adjusting for additional, unspecified covariates) reported significantly lower odds of past year quit attempts among menthol cigarette smokers (AOR = 0.98, 95% CI: 0.98 to 0.98). In Keeler et al. [44], the overall odds of past-year quit attempts between menthol and non-menthol smokers were no different. Both the 2017 and 2018 studies by Keeler at al [44, 45]. found that, among black smokers, menthol users were significantly more likely to report past-year quit attempts (2018: AOR = 1.39, 95% CI: 1.16 to 1.67; *p* < 0.001; 2017: AOR = 1.37, 95% CI: 1.16 to 1.61; *p* = 0.0002); no such differences were reported for other racial/ethnic subgroups. The majority of the results from these 14 studies reported no differences between menthol and non-menthol smoking in terms of quit attempts.

#### Rate of abstinence/quitting

Twenty-nine studies (from 33 references), presented below in Table 7**,** reported on rate of abstinence/quitting outcomes.

**Table 7:** Summary of Evidence Related to Rate of Abstinence/Quitting

Study	Sampling / Recruitment Strategy^**a**^, Data Collection Period	Study Findings	Study Quality
Decreased Rate of Abstinence/Quitting with Menthol Cigarette Use			
Thihalolipavan et al., 2014 [68]	New York City Nicotine Patch and Gum Program; 2012	Smoking menthol cigarettes was associated with a 10% lower prevalence of quitting (PR = 0.90, 95% CI: 0.83 to 0.97) after 3 to 6 weeks.	Poor
Lewis et al., 2014 [47]	Nielsen Homescan Panel; January 2004–December 2009	Menthol smokers had a significantly lower likelihood of quitting compared with non-menthol smokers (HR = 0.79, 95% CI: 0.64 to 0.99).	Fair
Rojewski et al., 2014 [61]	A trial of 166 weight-concerned smokers who smoked at least 10 CPD for at least a year and had at least one prior quit attempt; 2005–2009	Menthol smokers were significantly less likely to be abstinent; specifically, non-menthol smokers were 2.4 times more likely to report 7-day PPA Weeks 14 and 26 (Week 14: AOR = 2.40, 95% CI: 1.04 to 5.55; Week 26 AOR = 2.47, 95% CI: 1.40 to 5.90; *p* = 0.04).	Good
Faseru et al., 2013 [34]	KIS-III trial; community-based clinic sample serving a predominantly Black population; 2007–2010	Menthol cigarette use was associated with significantly lower odds of cotinine-verified 7-day PPA at the end of 7 weeks of treatment compared to non-menthol cigarette use; specifically, non-menthol, compared to menthol, smokers had 84% greater odds of 7-day PPA at week 7 (AOR = 1.84, 95% CI: 1.01 to 3.36; *p* < 0.05).	Good
No Difference in Rate of Abstinence/Quitting with Menthol Cigarette Use			
Keeler et al., 2018 [45]	Probability sample of U.S. households; personal and telephone interviews; May/August 2006 to January 2007, and May/August 2010 to January 2011	No difference between Black menthol and non-menthol smokers in the rate of successful cessation (≥3 months (AOR = 1.01, 95% CI: 0.70 to 1.45; p = NS). Similarly, no difference was found for the rate of successful cessation (≥3 months) between White menthol and non-menthol smokers (AOR = 0.94, 95% CI: 0.84 to 1.07).	Fair
Keeler et al., 2017 [44]	Probability sample of U.S. households; personal and telephone interviews; 2006 to 2007 and 2010 to 2011	No difference between menthol and non-menthol smokers in the odds of cessation (≥3 months (AOR = 0.92 95% CI: 0.83 to 1.03; *p* = 0.1470). Similarly, no difference for the odds of cessation in subgroup analyses of: Black menthol and non-menthol smokers (AOR = 1.03, 95% CI: 0.73 to 1.44; *p* = 0.8630); White menthol and non-menthol smokers (AOR = 0.94, 95% CI: 0.84 to 1.06; *p* = 0.3190); Asian menthol and non-menthol smokers (AOR = 0.98, 95% CI: 0.44 to 2.19; *p* = 0.9540); and Hispanic menthol and non-menthol smokers (AOR = 0.88, 95% CI: 0.60 to 1.28; *p* = 0.4980.	Fair
Winhusen et al., 2013 [71]	Randomized trial of U.S. substance use outpatient treatment program participants receiving smoking cessation treatment; Feb 2010-July 2012	No difference in effect for smoking cessation (as measured by 7-day PPA at week 10) between menthol and non-menthol cigarette type among either the cocaine-dependent (*p* = 0.81) or methamphetamine-dependent (*p* = 0.9) participants.	Fair
D’Silva et al., 2012 [30]	ClearWay Minnesota phone line; September 2009 – July 2011, 7-month post-registration follow-up survey March 2010–February 2011	No difference between menthol and non-menthol smokers in the odds of quitting (as assessed by 30-day PPA (AOR = 1.29, 95% CI: 0.77 to 2.15).	Fair
Nonnemaker et al., 2012 [50]	ALLTURS; U.S. school-based survey of middle and high school youth; 2000–2002	No difference between those who initiated smoking with menthol and non-menthol in quit rates (AOR = 1.18, 95% CI: 0.78 to 1.80; ref. = NM).	Good
Reitzel, 2011a [56]	Project BREAK FREE; Houston metro area; 2005–2007	No difference between menthol and non-menthol cigarette use in predicting prolonged abstinence from smoking among Black smokers in adjusted analyses (β = .33, SE = .32; χ2 = 1.06; *p* = .30; *n* = 457).	Fair
Reitzel, 2011b [57]	Project CARE; Texas; 2005–2007	No difference between menthol and non-menthol cigarette use in predicting prolonged abstinence from smoking in adjusted analyses (β = 0.05, SE = 0.25; χ2 = 0.04; *p* = 0.84).	Fair
Steinberg et al., 2011 [66]	Cessation study that enrolled 723 smokers age 16–78	No difference between menthol and non-menthol smokers in the odds of abstinence (7-day PPA) at 6 months after target quit date (AOR = 1.02, 95% CI: 0.66 to 1.58).	Good
Hyland et al., 2002 [40]; Hyland & Rivard, 2010 [41]	COMMIT cessation trial; modified random-digit-dial method of approximately 5400 HHs with focus on communities with the highest prevalence of non-Whites; 1988–2001	No differences in quit rates between menthol and non-menthol smokers who were smoking from 1988 to 2001 and had not attempted to quit (AOR = 0.84, 95% CI: 0.61 to 1.15), who had attempted to quit (AOR = 1.03, 95% CI: 0.71 to 1.48), or among the corresponding White sub-samples (no quit attempts: AOR = 0.79, 95% CI: 0.56 to 1.11; quit attempts: AOR = 0.96, 95% CI: 0.65 to 1.41). Also, no difference in quitting between menthol and non-menthol cigarette use in 1988 among: smokers in 1993 (AOR = 1.00, 95% CI: 0.90 to 1.11); White smokers (AOR = 0.94, 95% CI: 0.83 to 1.05); Black smokers (AOR = 1.04, 95% CI: 0.73 to 1.47); and Hispanic smokers (AOR = 1.22, 95% CI: 0.80 to 1.87).	Good
Cropsey et al., 2009 [28]	Female prison sample; June 2004–June 2006	No differences between menthol and non-menthol smokers in smoking cessation (as evaluated by 7-day PPA (Wald chi-square = 1.2; *p* = 0.272; and with interaction of race X menthol: Wald chi-square = 0.1; *p* = 0.27).	Fair
Fu et al., 2008 [36]	VA medical center sample; February–October 2002	No difference between menthol and non-menthol smokers in smoking abstinence (as assessed by self-reported 7-day PPA (AOR = 1.31, 95% CI: 0.95 to 1.82).	Good
Okuyemi et al., 2007 [52]	KIS trial; cessation program of an inner-city health center mostly serving a low-income Black population; March 2003–June 2004	No difference was found for 7-day PPA at week 26 (*p* = 0.93) between categorized age (< 50 versus ≥50 years) and menthol status. Further, among the < 50 years of age group, no difference between menthol and non-menthol smokers in cessation rates (AOR = 2.077, 95% CI: 0.944 to 4.569; *p* = 0.069). Likewise, among those ≥50 years, no difference between menthol and non-menthol cigarette use in abstinence (AOR = 1.676; 95% C1: 0.760 to 3.698; *p* = 0.221).	Good
Foulds et al., 2006 [35]	Convenience sample of patients attempting to quit at a specialist tobacco dependence treatment outpatient clinic; 2001–2006	At the four-week follow up, was no difference between menthol and non-menthol smokers in 7-day PPA (AOR = 1.36, 95% CI: 1.0 to 1.86).	Good
Pletcher et al., 2006 [54]	CARDIA; men and women in the U.S. age 18–30 at baseline with follow-up data through year 19; 1985–2000	No different between menthol and non-menthol smokers in quit rate (i.e., not currently smoking at any examination (AOR = 0.90, 95% CI: 0.68 to 1.19). There was also no difference in quitting between menthol and non-menthol smokers who tried to quit (AOR = 1.00, 95% CI: 0.71 to 1.42). In longitudinal analyses, no difference between menthol and non-menthol smokers in sustained smoking cessation (AOR = 0.71, 95% CI: 0.49 to 1.02; *p* = 0.06).	Good
Muscat et al., 2002 [49]	Newly diagnosed, non-surgical cancer patients; 1981–1999	In adjusted analyses, no difference was found between menthol and non-menthol cigarette use in continued smoking among Black participants (POR = 1.1, 95% CI: 0.8 to 1.4) and White participants (POR = 1.1, 95% CI: 1.0 to 1.3).	Fair
Schneller et al., 2020 [63]; Schneller, 2020 [64]	PATH; 12 September 2013 to 14 December 2014 (Wave 1), 23 October 2014 to 30 October 2015 (Wave 2)	No significant difference in the adjusted odds of menthol users reporting successful cessation at Wave 2 compared to non-menthol users, when adjusting for gender, age, race/ethnicity, education, and HSI (AOR = 1.09, 95% CI: 0.88–1.37, *p* = NS). In a similar model—replacing HSI (above) with CPD—the results were almost identically non-significant (RRR = 1.09, 95% CI: 0.87–1.35, *p* = NS)	Fair
Results of Mixed Significance in Rate of Abstinence/Quitting			
Sulsky et al., 2014 [67]	NHIS; 2005,2010; TUS-CPS; 2010/2011	Decrease with Menthol Cigarette Use According to the TUS-CPS data, menthol cigarette use among Black regular and daily smokers was significantly lower for the adjusted odds of abstinence for 1–3 years (regular smokers: AOR = 0.87, 95% CI: 0.80 to 0.95; daily smokers: AOR = 0.89, 95% CI: 0.81 to 0.98). No Difference According to the NHIS data, among White participants, there was no difference between menthol regular and daily smokers in the adjusted odds of past-year abstinence (regular smokers: AOR = 1.06, 95% CI: 0.95 to 1.18; daily smokers: AOR = 1.04, 95% CI: 0.82 to 1.33). No difference between White menthol and non-menthol regular and daily smokers in the adjusted odds of abstinence for 1–3 years (regular smokers: AOR = 0.97, 95% CI: 0.94 to 1.00; daily smokers: AOR = 0.98, 95% CI: 0.95 to 1.01). For participants whose race/ethnicity was other than White or Black, no difference for abstinence for 1–3 years between menthol and non-menthol smokers (regular smokers: AOR = 0.99, 95% CI: 0.91 to 1.08; daily smokers: AOR = 1.00, 95% CI: 0.92 to 1.09).	Good
Reitzel et al., 2013 [60]	Texas; lung cancer case-control study; February 1996–July 2001	Decrease with Menthol Cigarette Use Menthol cigarette use was significantly associated with a lower probability of short-term continuous smoking abstinence among White participants (β = − 1.56, SE = 0.79; χ2 = 3.96; *p* = 0.05). Racially stratified analyses also found a significant association of menthol cigarette use with 7-day PPA smoking abstinence through post-quit Week 3 among White participants (β = − 1.90, SE = 0.82; *p* = 0.02). No difference No significant effect of menthol cigarette use status on continuous short-term smoking abstinence (β = − 0.31, SE = 0.40; χ2 = 0.60; *p* = 0.44). Moreover, no difference between Black menthol, versus non-menthol, smokers for short-term continuous smoking abstinence (β = 0.54, SE = 0.55; χ2 = 0.95; *p* = 0.33); even after racially stratifying analyses, no difference between Black menthol and non-menthol smokers according to 7-day PPA (β = 1.00, SE = 0.67; *p* = 0.11).	Fair
Blot et al., 2011 [27]	40–79 year olds living in southern U.S. states; March 2002 – September 2009	Increase with Menthol Cigarette Use Adjusting for age and other covariates, White menthol, versus non-menthol, cigarette smokers were more likely to have quit smoking prior to study enrollment (AOR = 1.55, 95% CI: 1.41 to 1.70). No difference No difference between Black menthol and non-menthol cigarette smokers in the likelihood of quitting smoking prior to study enrollment (AOR = 1.03, 95% CI: 0.96 to 1.11).	Fair
Delnevo et al., 2010; Delnevo et al., 2011 [31, 32]	TUS-CPS, 2003,2006/2007	Adjusted odds of being a former smoker (menthol versus non-menthol) was measured across five sample restrictions: cigarette smokers and former smokers who quit in the past 5 years (restriction 1); cigarette smokers and former smokers who quit in the past 5 years who do not currently use other tobacco products (restriction 2); cigarette smokers and former smokers who quit in the past 5 years who have made a quit attempt (restriction 3); cigarette smokers and former smokers who quit in the past 5 years who have made a quit attempt and do not currently use other tobacco products (restriction 4); and past-year smokers (restriction 5, also adjusting for past-year cigarette tax increase). Decrease with Menthol Cigarette Use The odds of being a former smoker were significantly lower among menthol, versus non-menthol, smokers in the overall sample with the least restrictions (restriction 1; AOR = 0.91, 95% CI: 0.87 to 0.96). The same significant difference was consistently found across restrictions 2, 3, and 4 with AORs ranging from 0.90 to 0.92. Black menthol smokers were significantly less likely to be former smokers with restriction 1 (AOR = 0.81, 95% CI: 0.67, 0.98) and across all four additional sample restrictions with the range of AORs from 0.68 to 0.81. White menthol smokers had significantly lower odds of being a former smoker (AOR = 0.93, 95% CI: 0.88, 0.98) across three of the five sample restrictions (1, 2 and 3). Puerto Rican menthol smokers were consistently and significantly less likely to be former smokers across all five sample restrictions, with AORs ranging from 0.42 to 0.63. Increase with Menthol Cigarette Use Two of the five sample restrictions (2 and 4) reported significantly higher odds of being a former smoker among Mexican menthol, versus non-menthol, smokers with AORs of 1.34 and 1.35, respectively. No difference No difference between menthol and non-menthol smokers was found in the adjusted odds of being a former smoker for the overall sample (AOR =0.922, 95% CI: 0.847 to 1.004). Also, no difference between White menthol and non-menthol smokers in the odds of being a former smoker (restrictions 4 and 5); likewise, no difference between Hispanic menthol and non-menthol smokers (restrictions 1 to 4); and, no difference between Mexican menthol and non-menthol smokers (restrictions 1, 3, and 5).	Good
Reitzel, 2011c; Reitzel et al., 2011 [58, 59]	Project MOM; 2005–2007	Decrease with Menthol Cigarette Use Adjusting for age, partner status, income, and educational achievement, time, treatment group, CPD, and time to the first cigarette of the day, White female menthol, versus non-menthol, cigarette smokers were significantly less likely to maintain continuous abstinence (β = − 1.62, SE = 0.76; χ2 = 4.49; *p* = 0.03; AOR = 0.19, 95% CI: 0.04 to 0.89). No Difference Across the entire sample, no difference between menthol and non-menthol use in continuous abstinence from smoking through 26 weeks postpartum (β = − 0.32, SE = 0.30; *p* = 0.29; *n* = 297). No difference between Black female menthol and non-menthol smokers in continuous abstinence (β = − 1.12, SE = .64; c2 = 3.06; *p* = .08; *n* = 96); likewise, no difference between Latina female menthol and non-menthol smokers in continuous abstinence (β = .46, SE = .50; c2 = .86; *p* = .35; *n* = 93).	Good
Trinidad et al., 2010 [69]	TUS-CPS; 2003, 2006–2007	Descrease with Menthol Cigarette Use The odds of successful quiting for ≥6 months among former smokers was significantly less likely in menthol, versus non-menthol, smokers, across all race/ethnicity subgroups evaluated: White smokers (AOR = 0.28, 95% CI: 0.25 to 0.33); Black smokers (AOR = 0.23, 95% CI: 0.17 to 0.31); Asian-American/Pacific Islander smokers (AOR = 0.22, 95% CI: 0.11 to 0.45); and Hispanic/Latino smokers (AOR = 0.48, 95% CI: 0.34 to 0.69). No Difference No difference between Native American/Alaskan Native former menthol and non-menthol smokers in the odds of successful quiting for ≥6 months (AOR = 0.49, 95% CI: 0.14 to 1.71).	Good
Gandhi et al., 2009 [37]	Outpatient tobacco treatment clinic patients; January 2001–June 2005	Decrease with Menthol Cigarette Use The odds of Black menthol, versus non-menthol, smokers’ abstinence were significantly lower at 4 weeks (measured by 7-day PPA (AOR = 0.32, 95% CI: 0.16 to 0.62) and at 6 months post-quit (AOR = 0.48, 95% CI: 0.25 to 0.90). Hispanic menthol, versus non-menthol, smokers’ odds of abstinence at 4 weeks post-quit were also significantly lower (AOR = 0.43, 95% CI: 0.1 to 0.9). No Difference No difference between White menthol and non-menthol smokers in the likelihood of abstinence at 4 weeks (AOR = 0.96, 95% CI: 0.72 to 1.20) or 6 months post-quit (AOR = 1.0, 95% CI: 0.8 to 1.4). Also, no difference between Hispanic menthol and non-menthol smokers in the odds of abstinence at 6 months (AOR = 0.64, 95% CI: 0.2 to 1.80).	Fair
Gundersen et al., 2009 [39]	NHIS-CCS; 2005	Decrease with Menthol Cigarette Use Subgroup analysis found that Hispanic menthol, versus non-menthol, smokers were significantly less likely to have quit smoking (AOR = 0.61, 95% CI: 0.39 to 0.97; *p* = 0.04). When Black and Hispanic smokers were combined (defining a “non-White” subsample), non-White menthol, versus non-menthol, smokers were significantly less likely to have quit smoking (AOR = 0.55, 95% CI: 0.43 to 0.71; *p* < 0.01). Increase with Menthol Subgroup analysis found that White menthol, versus non-menthol, smokers were significantly more likely to have quit smoking (AOR = 1.17, 95% CI: 1.00 to 1.36; *p* < 0.05). No difference Without stratifying for race/ethnicity, no difference between menthol and non-menthol smokers in smoking cessation (AOR = 1.05, 95% CI: 0.92 to 1.21). Subgroup analysis: no difference between Black menthol and non-menthol smokers in the odds of smoking cessation (AOR = 0.78, 95% CI: 0.56 to 1.09).	Good
Okuyemi et al., 2003 [51]	KIS trial; August 2000–November 2000.	Decrease with Menthol Cigarette Use Although biochemically verified 7-day PPA abstinence was measured at both 6 weeks and 6 months, authors only modeled for 6 weeks “because univariate analysis did not reveal significant differences in abstinence rates between menthol and non-menthol smokers at 6 months.” In addition, overall modeled results were not presented. Among adults < 50 years of age, non-menthol, versus menthol, smokers had significantly higher odds of quitting (AOR = 2.02, 95% CI: 1.03 to 3.95). No Difference No difference between menthol and non-menthol smokers > 50 years of age in abstinence rates (*p* = 0.57).	Good

^a^ Details of sampling and recruitment strategies for the data sources can be found in Table 3: Study, Data Set, and Sample Characteristics

Four studies found that menthol smokers had significantly lower odds of quitting than non-menthol smokers; two studies reported 7-day PPA (between weeks 14 and 26 [61]; and at the previous 7 days and at week 7 [34]), while two studies examined cessation at different time points (1 year abstinence from purchasing a pack of cigarettes [47]; and abstinence at 3 to 6 week follow-up [68]).

Sixteen studies (from 18 references) found no difference in the rate of abstinence between menthol and non-menthol smokers, both overall and within subgroup analyses, in terms of: 7-day PPA in six studies [28, 35, 36, 52, 66, 71]; 30-day PPA in one study [30]; quit rates from baseline to follow-up in three studies from four references [40, 41, 50, 54]; cessation of greater than 3 months in two studies [44, 45]; PA in two studies [56, 57]; successful cessation between two survey waves in one study from two references [63, 64]; and past-year abstinence in one study [49].

Nine studies (from 11 references), reported mixed significance [27, 31, 32, 37, 39, 51, 58–60, 67, 69]. Using NHIS data, Sulsky et al. [67] found that White menthol and non-menthol regular and daily smokers were no different in odds of past-year abstinence; similar results were observed in Black menthol and non-menthol daily smokers. Using TUS-CPS data, the authors found no significant difference in one- to three-year abstinence between White menthol and non-menthol smokers (both regular and daily). For other race/ethnicities, no difference was detected between menthol and non-menthol use in terms of abstinence among regular and daily smokers. However, for Black daily (AOR = 0.89, 95% CI: 0.81 to 0.98) and regular (AOR = 0.87, 95% CI: 0.80 to 0.95) smokers, menthol use was significantly associated with lower odds of abstinence.

Reitzel et al. [60] found that menthol and non-menthol smokers were no different in terms of short-term abstinence for the overall sample. However, among White participants, menthol use predicted a significant decrease in short-term abstinence (β = − 1.56, SE = 0.79; χ2 = 3.96; *p* = 0.05) as well as 7-day PPA (β = − 1.60, SE = 0.79; χ2(1) = 4.06; *p* = .04; *n* = 132). No such differences were reported for either outcome among Black participants (short-term abstinence: β = 0.54, SE = 0.55; *p* = 0.33; and 7-day PPA: β = 1.00, SE = 0.67; *p* = 0.11).

Blot et al. [27] found that White menthol smokers had significantly greater odds of having quit compared with non-menthol smokers (AOR = 1.55, 95% CI: 1.41 to 1.70); however, Black menthol and non-menthol smokers were no different.

Trinidad et al. [69] reported that, among White, Black, Asian-American/Pacific Islander, and Hispanic participants, menthol smoking was associated with significantly lower odds of abstinence greater than 6 months (AORs ranged from 0.28 to 0.48). However, among Native American/Alaskan native participants, menthol and non-menthol smokers were no different in terms of the odds of abstinence greater than 6 months.

Delnevo et al. [31, 32] reported on the odds of being a former smoker across five racial/ethnic subgroups and the following five sample restrictions (according to past and current smoking status): former smokers who quit within the past 5 years and all current smokers (regardless of quit attempt history); former smokers who quit within the past 5 years and all current smokers (regardless of quit attempt history), both of whom currently do not use other tobacco products; former smokers who quit within the past 5 years and current smokers who reported ever having made a quit attempt; former smokers who quit within the past 5 years and current smokers who reported ever having made a quit attempt, both of whom currently do not use other tobacco products; and, past 12-month cigarette smokers who made a quit attempt or quit (i.e., former smokers). Among the overall sample, across four of the five restrictions, menthol cigarette smokers were significantly less likely than non-menthol smokers to be former smokers with AORs ranging from 0.90 to 0.92.

Black menthol smokers were significantly less likely to be former smokers compared to Black non-menthol smokers in all five restrictions with AORs ranging from 0.68 to 0.81. White menthol, versus non-menthol, smokers were significantly less likely to be a former smoker across three restrictions. However, Hispanic menthol and non-menthol smokers were no different across four of the five restrictions; and, were significantly less likely to be a former smoker in one restriction.

In Reitzel’s ‘Project Mom’ [58, 59], menthol cigarette use did not predict continuous abstinence from smoking. However, among White women, menthol smokers were significantly less likely to maintain continuous abstinence compared to non-menthol smokers (AOR = 0.19, 95% CI: 0.04 to 0.89).

Gandhi et al. [37] found no difference between White menthol and non-menthol smokers in odds of abstinence at both 4 weeks and 6 months. Black menthol smokers had significantly lower odds of abstinence compared to Black non-menthol smokers at both time points, 4 weeks (measured by 7-day PPA) (AOR = 0.32, 95% CI: 0.16 to 0.62) and at 6 months post-quit (AOR = 0.48, 95% CI: 0.25 to 0.90). Hispanic menthol smokers had significantly lower odds of abstinence at 4 weeks compared to Hispanic non-menthol smokers (AOR = 0.43, 95% CI: 0.1 to 0.9); at 6 months, Hispanic menthol and non-menthol smokers were no different in odds of abstinence.

Gundersen et al. [39] suggested no significant difference in being a former smoker between menthol and non-menthol smokers in the overall sample, and among Black smokers. However, odds of being a former smoker were significantly higher for White menthol compared to White non-menthol smokers (AOR = 1.17, 95% CI: 1.00 to 1.36; *p* < 0.05). Odds of being a former smoker were significantly lower for Hispanic menthol compared to Hispanic non-menthol smokers (AOR = 0.61, 95% CI: 0.39 to 0.97; *p* = 0.04), and for non-White menthol compared to non-White non-menthol smokers (AOR = 0.55, 95% CI: 0.43 to 0.71; *p* < 0.01).

Okuyemi et al. [51] reported no significant difference in odds of quitting between menthol and non-menthol smokers among adults ≥50 years of age; however, in adults < 50 years of age, the odds of quitting for menthol smokers were significantly lower for menthol smokers (AOR = 2.02, 95% CI: 1.03 to 3.95).

Across the 28 studies, the majority of studies (15 studies) found no difference between menthol and non-menthol smokers in the rate of abstinence. Four studies reported that menthol smokers were significantly less likely to quit smoking and nine studies reported results of mixed significance based on various stratifications. Overall, the evidence for this outcome was inconsistent for the association between menthol cigarette use and the rate of abstinence/quitting.

#### Change in smoking quantity/frequency

Five studies (from six references), presented in Table 8, provided adjusted analysis of change in smoking quantity/frequency.

**Table 8:** Summary of Evidence Related to Change in Smoking Quantity/Frequency

Study	Sampling / Recruitment Strategy^**a**^, Data Collection Period	Study Findings	Study Quality
Increase in Smoking Quantity/Frequency with Menthol Cigarette Use			
Azagba et al., 2020 [26]	NYTS; 2017–2018	Significantly higher odds of using at least 10 days (versus (1–9 days) in the past 30 days compared with non-menthol cigarette smokers, in the full sample (AOR = 1.48, 95% CI, 1.14 to 1.94; *p* < 0.05) and in the stratified analyses for both middle school students (AOR = 2.36, 95% CI, 1.01 to 5.49; *p* < 0.05) and high school students (AOR = 1.41, 95% CI, 1.09 to 1.82; *p* < 0.05). Significantly higher odds of using at least 20 days (versus (1–19 days) in the past 30 days compared with non-menthol cigarette smokers, in the full sample AOR = 1.62, 95% CI, 1.15 to 2.28; *p* < 0.05) and in the stratified analyses for both middle school students (AOR = 3.76, 95% CI, 1.21 to 11.71; *p* < 0.05) and high school students (AOR = 1.49, 95% CI, 1.07 to 2.07; *p* < 0.05).	Fair
No Difference in Change in Smoking Quantity/Frequency with Menthol Cigarette Use			
Hyland et al., 2002 [40]; Hyland & Rivard, 2010 [41]	COMMIT; 1988–2001	No difference between menthol and non-menthol smokers in the odds of reducing daily cigarette use over 3 years (AOR = 0.83, 95% CI: 0.64 to 1.07); subgroup analyses of Black and White smokers also found no difference. Similarly, change in CPD in 1993 according to cigarette type smoked in 1988 was no different in the overall sample (β-coefficient = 0.11, 95% CI: − 0.38 to 0.60), nor in Black, White, or Hispanic subgroups.	Good
Gubner et al., 2018 [38]	Convenience sampling from each of 24 substance use disorder treatment centers (in the National Institute on Drug Abuse Clinical Trials Network), with self-administered surveys conducted during on-site visits; April to December 2015	Applying an adjusted logistic regression model, the study found that the number of CPD was not significantly associated with menthol use (AOR = 1.01, 95% CI: 0.98 to 1.00; *p* = 0.48).	Fair
Results with Mixed Significance in Smoking Quantity/Frequency			
Reitzel, 2011c [58]	Project MOM; 2005–2007	Decrease with Menthol Cigarette Use Black female menthol, versus non-menthol, smokers reported substantially less cigarette reduction (measured by CPD) over the course of 26 weeks (β = 3.82, SE = 3.77; *p* = 0.02; *n* = 71). No Difference No difference among female menthol and non-menthol smokers, overall, in changes in smoking frequency over the 26-week period (β = − 0.38, SE = 1.15; t = −.33; *p* = .74; *n* = 222).	Good
Sawdey et al., 2020 [62]	NYTS; 2011–2018	Increase with Menthol Cigarette Use Odds of frequent smokers (on ≥20 days in the past 30 days) being menthol smokers was significantly higher than being non-menthol smokers (AOR = 1.57, 95% CI: 1.08–2.29). No Difference No significant difference in the odds of moderate smokers (on 6 to 19 days in the past 30 days) being menthol versus non-menthol smokers (AOR = 1.17, 95% CI: 0.86–1.59). The overall *p* = value across both groups—frequent and moderate smokers—was non-significant (*p* = 0.064).	Good

^a^ Details of sampling and recruitment strategies for the data sources can be found in Table 3: Study, Data Set, and Sample Characteristics

Azagba et al. [26] found that menthol cigarette smokers had significantly higher odds of using cigarettes at least 10 days (versus (1–9 days) in the past 30 days compared with non-menthol cigarette smokers, in the full sample (AOR = 1.48, 95% CI, 1.14 to 1.94; *p* < 0.05) and among both middle (AOR = 2.36, 95% CI, 1.01 to 5.49; *p* < 0.05) and high school students (AOR = 1.41, 95% CI, 1.09 to 1.82; *p* < 0.05). Similarly, menthol cigarette smokers had significantly higher odds of using at least 20 days (versus (1–19 days) in the past 30 days compared with non-menthol cigarette smokers, in the full sample AOR = 1.62, 95% CI, 1.15 to 2.28; *p* < 0.05) and among both middle (AOR = 3.76, 95% CI, 1.21 to 11.71; *p* < 0.05) and high school students (AOR = 1.49, 95% CI, 1.07 to 2.07; *p* < 0.05).

One study, from two references [40, 41], reported no difference between menthol and non-menthol cigarette smokers for changes in smoking frequency; similarly, one study reported that cigarettes per day (CPD) was not significantly associated with menthol cigarette use [38].

Two studies reported mixed significance. Reitzel [58] found that Black female menthol smokers reported substantially less cigarette reduction (measured by CPD) over the course of 26 weeks (β = 3.82, SE = 3.77; *p* = 0.02; *n* = 71), but no difference was found in changes in smoking frequency for the overall sample. Sawdey et al. [62] found no significant difference in the odds of moderate smokers (on 6 to 19 days in the past 30 days) being menthol versus non-menthol smokers (AOR = 1.17, 95% CI: 0.86–1.59); however, the odds of frequent smokers (on ≥20 days in the past 30 days) being menthol smokers was significantly higher than being non-menthol smokers (AOR = 1.57, 95% CI: 1.08–2.29). The overall p = value across both groups was non-significant (*p* = 0.064).

The overall evidence base for this outcome was limited by the small number of included studies, and the mixed significance of findings across studies precludes clear conclusions from the available evidence.

#### Return to smoking/relapse

Two studies, presented in Table 9, provided analyses of return to smoking/relapse.

**Table 9:** Summary of Evidence Related to Return to Smoking/Relapse

Study	Sampling / Recruitment Strategy^**a**^, Data Collection Period	Study Findings	Study Quality
Increased Return to Smoking/Relapse with Menthol Cigarette Use			
Muench & Juliano, 2017 [48]	Sample was recruited from the Washington DC metropolitan area through flyers, word of mouth, and online advertisements	According to logistic regression model results, menthol cigarette use was significantly associated with greater lapse risk (AOR = 3.474, *p* < 0.05). Similarly, menthol, versus non-menthol, use was significantly higher for risk of lapsing within the first 48 h of abstinence (HR = 2.798, Wald statistic = 2.79; *p* = 0.048).	Good
Pletcher et al., 2006 [54]	CARDIA; 1985–2000	The odds of returning to smoking post-cessation were significantly higher for menthol, versus non-menthol, smokers (AOR = 1.89, 95% CI: 1.17 to 3.05; *p* = 0.009).	Good

^a^ Details of sampling and recruitment strategies for the larger and/or national surveys can be found in Table 3: Study, Data Set, and Sample Characteristics

In Muench and Juliano [48], menthol smokers were at a significantly greater risk of lapsing compared with non-menthol smokers, in both the univariate regression (AOR = 3.474, *p* < 0.05) and lapse survival curve analyses (HR = 2.798, Wald statistic = 2.79; *p* = 0.048). Pletcher et al. [54] reported that young adult menthol smokers had a significantly higher likelihood of returning to smoking, compared to non-menthol smokers (AOR = 1.89, 95% CI: 1.17 to 3.05; *p* = 0.009).

These results suggest a higher likelihood of menthol smokers relapsing. However, the small number of studies—neither based on nationally representative samples—limit the generalizability of the findings.

### Sensitivity analyses

Three sensitivity analyses were conducted in order to test whether the results differed after more stringent inclusion and exclusion criteria were applied. Overall, results from the sensitivity analyses suggested little to no change. Full details on the sub-group analysis and sensitivity analyses are provided in SUPPLEMENTAL SECTION 5: Sensitivity Analyses.

### Results of meta-analyses

After screening all included adjusted studies, pooled data were included and extracted for two outcome measures: nine studies in the meta-analyses for quit attempts [25, 33, 41, 42, 44, 46, 53, 54, 70] and 12 studies for abstinence [27, 32, 34–37, 39, 51, 52, 54, 61, 66]. Full details are provided in SUPPLEMENTAL SECTION 6: Characteristics, Definitions, and Covariates of Studies Included in the Meta-Analysis.

#### Adjusted odds of reporting a quit attempt (past year or ever)

**Fig. 2:**
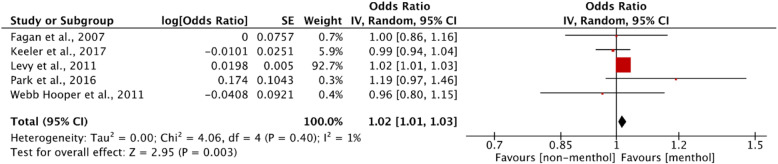
Forrest plot, Past Year Quit Attempts among Menthol Smokers Compared to Non-Menthol Smokers

**Fig. 3:**
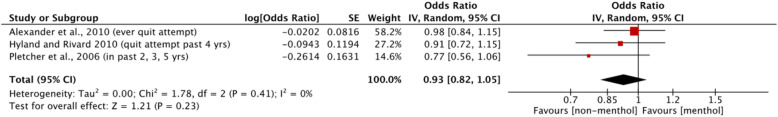
Forrest plot, Ever Quit Attempts among Menthol Smokers Compared to Non-Menthol Smokers

**Fig. 4:**
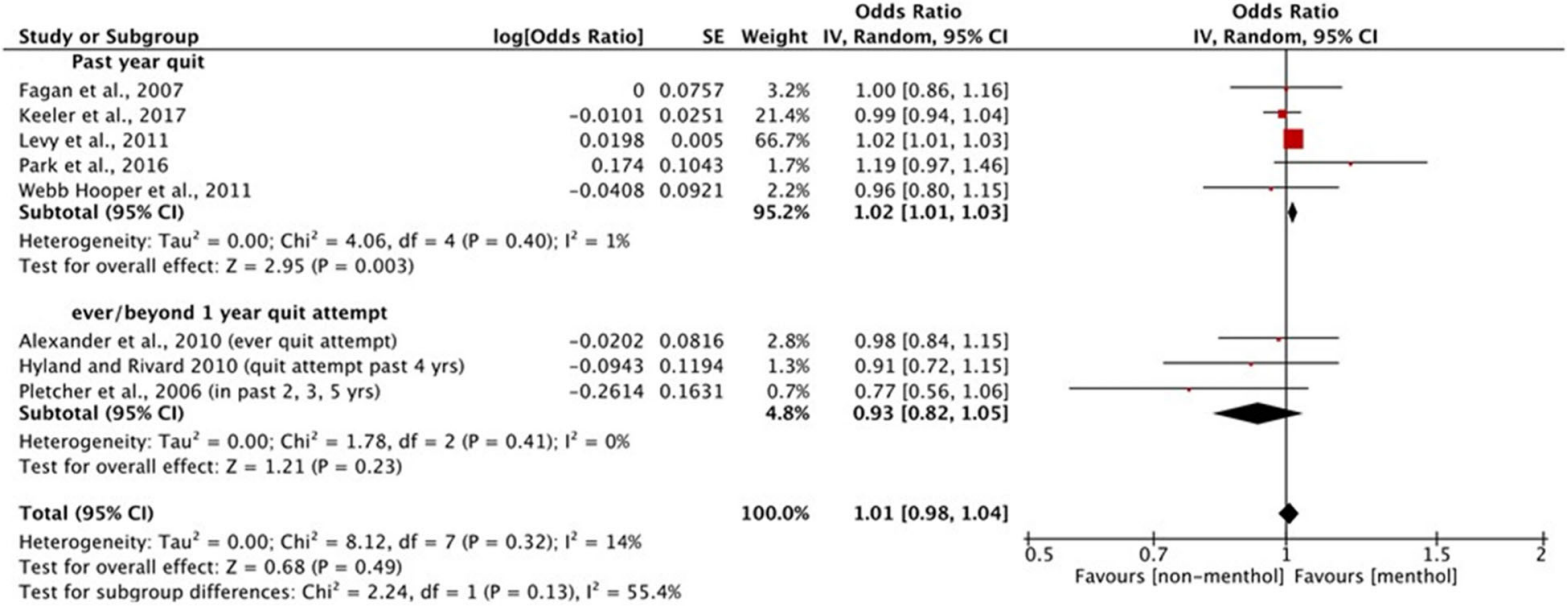
Forrest plot, Sub-Group Analysis for Past Year and Ever Quit Attempts among Menthol Smokers Compared to Non-Menthol Smokers

Results from five studies were pooled to measure the association of menthol use and past year quit attempts. Pooled results from five studies (Fig. 2) showed a significant association between menthol, versus non-menthol, cigarette use and the increasing odds for past year quit attempts (OR = 1.02, 95% CI: 1.01 to 1.03, *p*-value = 0.003, I2 = 1%). However, pooled result was different for a group of studies measuring ever quit attempts (Alexander et al. [2010]: AOR = 0.98, 95% CI: 0.83 to 1.15), any quit attempts between 2001 to 2005 (Hyland and Rivard, [2010]: AOR = 0.91, 95% CI: 0.72 to 1.15), and any quit attempt in the past 2, 3, or 5 years (Pletcher et al. [2006]: 0.77, 95% CI: 0.57 to 1.06), finding no significant difference in the odds of making a quit attempt among menthol users compared to non-menthol cigarette smokers (OR = 0.93, 95% CI, 0.82 to 1.05, *p* = 0.23, I2 = 0%; Fig. 3) [25, 41, 54]. In a subgroup analysis of the five studies with past year quit attempts as one group, and the group of three studies measuring ever quit attempts, any quit attempts between 2001 to 2005, and any quit attempt in the past 2, 3, or 5 years (Fig. 4), results remained non-significant (OR = 1.01, 95% CI: 0.98 to 1.04, *p* = 0.49, I2 = 14%). Test for subgroup difference showed moderate heterogeneity (I2 = 55.4%).

**Fig. 5:**
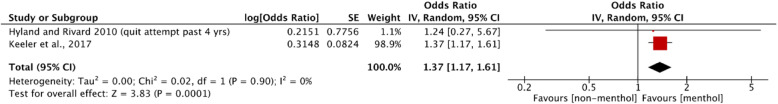
Forrest plot, Ever Quit Attempts among Black Respondents

**Fig. 6:**
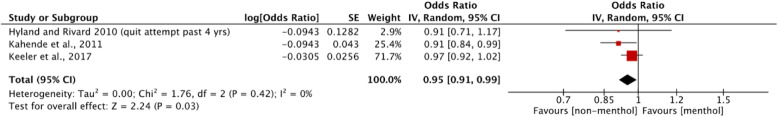
Forrest plot, Ever Quit Attempts among White Respondents

Results from two studies were pooled to measure for the association of menthol cigarette use and quit attempts (past year and quit attempts between 2001 and 2005) among Black participants (Fig. 5) [41, 44]. Pooled results showed a significant increase in the odds of Black menthol, versus non-menthol, smokers reporting quit attempts (OR = 1.37, 95% CI: 1.17 to 1.61, *p* = 0.00001, I^2^ = 14%). In contrast, among White menthol respondents in three studies (Fig. 6), the odds of making a quit attempt were significantly lower for menthol compared to non-menthol smokers (OR = 0.95, 95% CI: 0.91 to 0.99, I^2^ = 0%) [41, 42, 44].

#### Adjusted odds of abstinence (no definition and 7-day PPA)

**Fig. 7:**
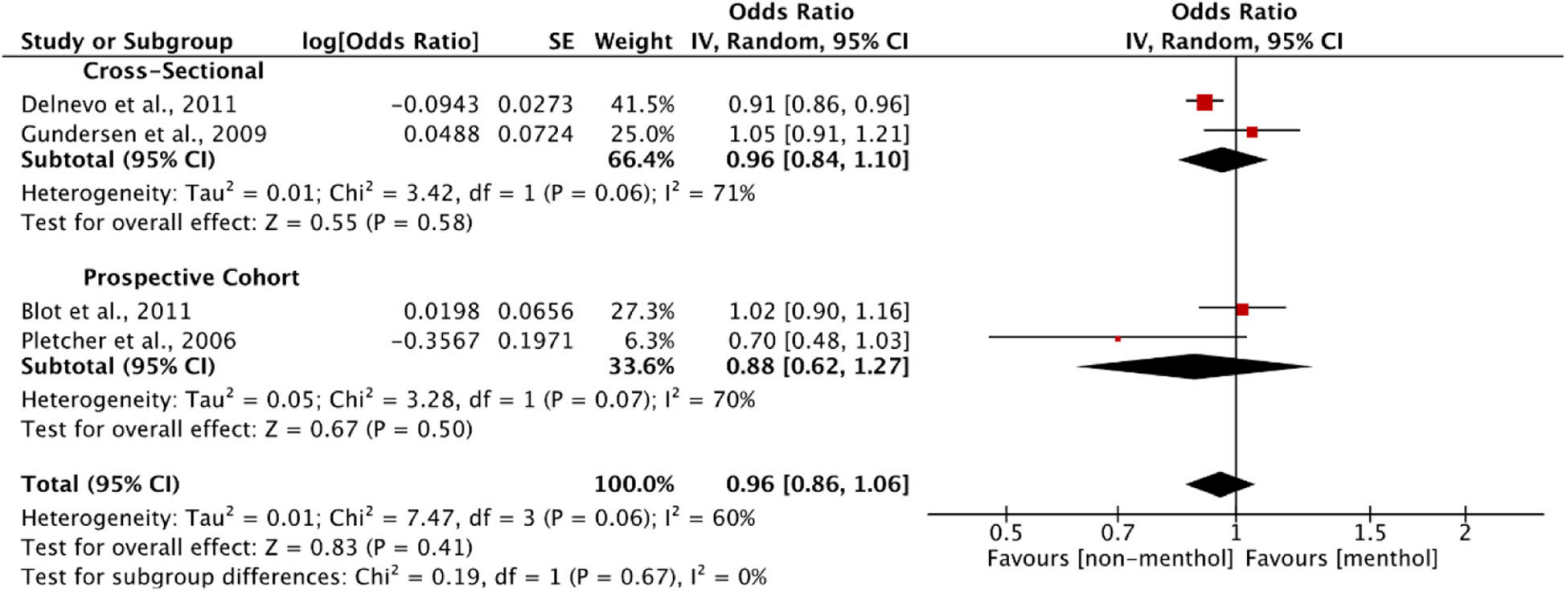
Forrest plot, Abstinence with no Specified Duration between Study Designs and All Studies

Four studies presented results for the association of menthol use and abstinence (self-reported) with no specified duration of abstinence. Two of the studies were cross-sectional in design [32, 39], and two were prospective cohort [27, 54]. Pooled results of cross-sectional studies showed that odds of abstinence with no defined duration among menthol smokers compared to non-menthol smokers was not significant (OR = 0.96, 95% CI: 0.84 to 1.10, *p* = 0.58, I^2^ = 71%). A non-significant result was likewise found in synthesis of prospective cohorts (OR = 0.88, 95% CI: 0.62 to 1.27, *p* = 0.50, I^2^ = 70%). Synthesizing the results of the four studies showed that the association of abstinence with no defined duration among menthol smokers compared to non-menthol smokers was not significant (OR = 0.96, 95% CI: 0.86 to 1.06, *p* = 0.41, I^2^ = 60%; Fig. 7). Test of subgroup differences between both groups (cross-sectional and longitudinal) manifested low heterogeneity (I^2^ = 0%).

**Fig. 8:**
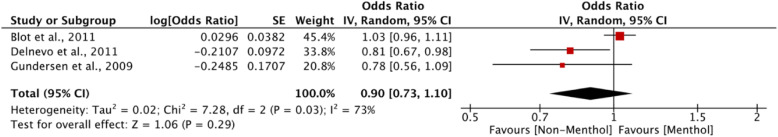
Forrest plot, Abstinence with no Specified Duration among Black Respondents

**Fig. 9:**
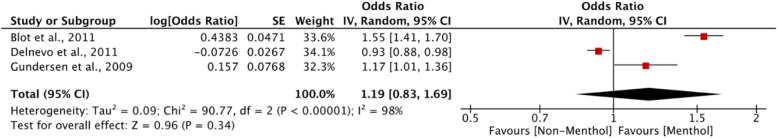
Forrest plot, Abstinence with no Specified Duration among White Respondents

Three studies presented results for the association of menthol use and abstinence with no specified duration of abstinence for Black participants [27, 32, 39]. Pooled results (Fig. 8) showed that the association between abstinence with no defined duration among menthol smokers compared to non-menthol smokers was not significant (OR = 0.90, 95% CI: 0.73 to 1.10, *p* = 0.29, I^2^ = 73%). Studies likewise allowed for analysis of association of menthol use and abstinence from smoking with no specified duration of abstinence for White participants [27, 32, 39]. Similar to Black participants, among White participants, results showed that the association of abstinence with no defined duration among menthol smokers compared to non-menthol smokers was not significant (OR = 1.19, 95% CI: 0.83 to 1.69, *p* = 0.34, =98%; Fig. 9). The heterogeneity was noted to be high for this analysis.

**Fig. 10:**
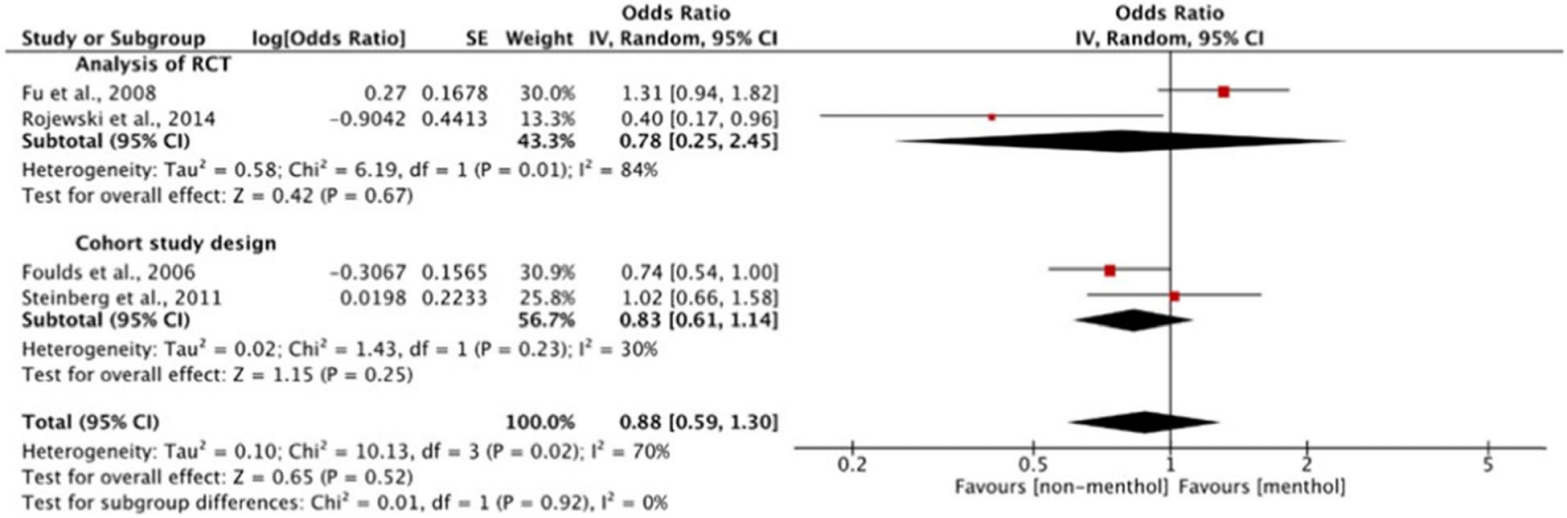
Forrest plot, 7-Day PPA between Study Designs and All Studies

Four cohort studies presented results for the association of menthol use and abstinence from smoking measured by 7-day PPA. For purposes of the following analysis, the studies were grouped by their specific research design. Two of the studies were analyses of RCT by design [36, 61], and two were cohort in nature [35, 66]. Seven-day PPA was self-reported at 4 weeks follow-up for Foulds et al. [35], self-reported at 2 years for Fu et al. [36], and self-reported and eCO verified for Steinberg et al. [66] and Rojewski et al. [61] at 26 weeks follow-up. All published AORs in the study used in the meta-analysis were standardized to have non-menthol use as the reference group [35, 61]. Pooled results of analyses from all four studies (Fig. 10) showed that odds of 7-day PPA among menthol smokers compared to non-menthol smokers was not significant (OR = 0.88, 95% CI: 0.59 to 1.30, *p* = 0.52, I^2^ = 70%). Similarly, results of longitudinal studies alone and RCT studies alone (Fig. 10) showed that odds of 7-day PPA among menthol smokers compared to non-menthol smokers was not significant (cohort: OR = 0.83, 95% CI: 0.61 to 1.14, *p* = 0.25, I^2^ = 30%; RCT: OR = 0.78, 95% CI: 0.25 to 2.45, *p* = 0.67, I^2^ = 84%). Test for subgroup difference showed low heterogeneity (I^2^ = 0%).

**Fig. 11:**
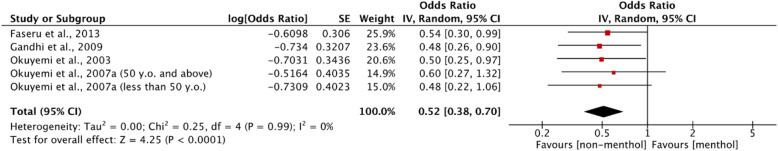
Forrest plot, 7-Day PPA among Black Respondents

Four studies presented results for the association of menthol use and 7-day PPA among Black participants [34, 37, 51, 52]. For the four studies, 7-day PPA was self-reported at 4 weeks follow-up for Gandhi et al. [37], self-reported at 6 weeks for Okuyemi et al. [51], cotinine verified (cut-off< 15 ng/ml) for Faseru et al. [34] at 7 weeks follow-up, and cotinine verified (cut-off< 20 ng/ml) and eCO verified (< 10 ppm) for Okuyemi et al. [52] at 26 weeks follow-up. All published AORs in the study used in the meta-analysis were standardized to have non-menthol use as the reference group [34, 51, 52]. Results showed that the odds for Black menthol smokers exhibiting 7-day PPA were significantly lower when compared to Black non-menthol smokers (OR = 0.52, 95% CI: 0.38 to 0.70, *p* < 0.0001, I^2^ = 0; Fig. 11).

**Fig. 12:**
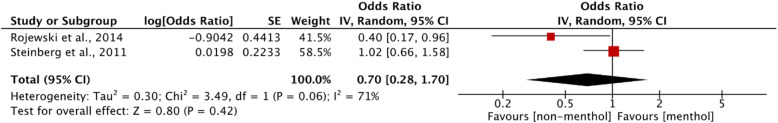
Forrest plot, eCO verified 7-Day PPA

A sensitivity analysis was conducted for eCO verified 7-day PPA (≤10 ppm) with two studies (Fig. 12) [61, 66]. Rojewski et al. [61] was standardized to have non-menthol use as the reference group. Meta-analysis results showed that the odds for eCO verified 7-Day PPA among menthol smokers compared to non-menthol smokers was not significant (OR = 0.70, 95% CI: 0.28 to 1.70, *p* = 0.42, I^2^ = 71%).

## Stength of evidence

Table 10 provides the SOE for the outcome measures used in the current review to examine the association between menthol cigarette use and cessation outcomes. Most measures were “indirect” and limited by the varying and/or undefined measures of abstinence. As presented in Table 11, the overall strength of evidence for an association between menthol cigarette use and smoking cessation was graded as “low” based on deficiencies in the available evidence base.

**Table 10:** Strength of Evidence Assessment by Measure (Adjusted Analyses)

	Study limitations	Directness	Consistency	Precision	SOE	CQ assessment
**Duration of abstinence**	Low	Direct	Inconsistent	Precise	**Low**	Acceptable
**Quit Attempts**	Low	Indirect	Inconsistent	Imprecise	**Insufficient**	Acceptable
**Rate of abstinence/quitting**	Low	Direct	Inconsistent	Imprecise	**Moderate**	Acceptable
**Change in quantity/frequency**	Low	Indirect	Consistent	Imprecise	**Insufficient**	Acceptable
**Return to smoking/relapse**	Low	Direct	Inconsistent	Imprecise	**Insufficient**	Inconclusive

*CQ* Contextual Question, *SOE* Strength of Evidence

**Table 11:** Overall Strength of Evidence Assessment (Adjusted Analyses)

Measure Name	CQ Assessment	Measure SOE	Overall SOE
Duration of abstinence	Acceptable	Low	**Low**
Quit attempts	Acceptable	Insufficient	
Rate of abstinence/quitting	Acceptable	Moderate	
Change quantity/frequency	Acceptable	Insufficient	
Return to smoking/relapse	Inconclusive	Insufficient	

*CQ* Contextual Question, *SOE* Strength of Evidence

## Discussion

The findings in this systematic review differ from several existing literature reviews on this topic. The 2013/2015 FDA Report/Addendum [6, 7] concluded that menthol in cigarettes was *“likely associated with reduced success in smoking cessation, especially among Black menthol smokers.”* That finding was not supported by this newer, more comprehensive review. Similarly, the evidence that contributed to this review does not support the conclusion in the 2011 Report by the FDA’s Tobacco Products Scientific Advisory Committee [5] that *“[e] vidence is sufficient to conclude that a relationship is more likely than not that the availability of menthol cigarettes results in lower likelihood of smoking cessation in Blacks.”*

Studies in the qualitative synthesis of this review were considered to provide the best available evidence on any differential impact of menthol versus non-menthol cigarette use on smoking cessation. Across studies, a variety of sampling and recruitment methods were used with varying definitions of current smoking and abstinence, and a range of study designs that, in many instances, did not directly address the current research question. Further, the available studies provided evidence that was inconsistent and imprecise—both across studies and within the same study.

Analyses of large cross-sectional studies yielded inconsistent findings. Among studies that used data from nationally representative samples, TUS-CPS and NHIS, population and sub-population results were mixed, based on modeling variation or definitions used; specifically, significantly positive and negative associations between menthol cigarette use and smoking cessation were reported, as well as numerous non-significant findings.

Clinical trials are designed to assess associations between interventions and outcomes, providing the temporal component that cross-sectional data lack. No clinical trials included in this review were designed with menthol cigarette use as the “intervention” to which participants were assigned. Therefore, these studies were re-classified as short-term prospective cohort studies. There was no consistent pattern of a differential impact of menthol versus non-menthol cigarette use on smoking cessation, even when data were stratified by type of cessation intervention, duration of intervention and follow up, or definition of outcome measure (including biochemical validation of self-reported abstinence). Both the shortest (6 weeks) and the longest (12 months) clinical studies found mixed or equivalent results. In addition, trials of cessation inherently include self-selected participants at least interested or motivated to quit smoking. Relying solely—or mainly—on clinical trial data to draw conclusions about the association between menthol cigarette use and smoking cessation will yield a result with limited generalizability to the overall smoking population.

The included prospective studies varied in follow-up duration — a critical factor in assessing the durability of cessation. Of the 11 prospective cohort studies that reported cessation, nine reported outcomes at 6 months or longer post-baseline. Specifically, three reported outcomes at 6 to 12 months, one followed participants for 1 to 2 years, one followed participants for 3 to 5 years, and four assessed outcomes beyond 5 years post-baseline. Two of the three 6- to 12-month cohort studies included a cessation intervention of some form — 7-day and 30-day PPA. The third 6- to 12-month cohort study reported continuous abstinence.

In the longer-term cohort studies, results were of mixed significance. COMMIT (a community-based public health intervention conducted in 11 matched pairs of communities) assessed menthol smoking at baseline in 1988; participants were interviewed again in 1993, 1998, 2001, and 2005. Investigators found no difference between menthol, versus non-menthol, smokers and smoking cessation during 17 years of follow up. The CARDIA study, a cohort of young adults at baseline, found no association between menthol cigarette use and cessation at 15-year follow up. However, a significantly positive association between menthol cigarette use and the risk of smoking relapse was identified. Finally, a study that investigated the association between menthol smoking and quit rate found that menthol smokers had a significantly lower likelihood of quitting compared with non-menthol smokers.

Return to smoking/relapse and change in smoking quantity/frequency were each reported by only two studies. Data were too limited to draw a reliable conclusion about the association between menthol cigarette use and either measure. Quit attempts — making at least one attempt and the number of quit attempts per person — were reported by several studies, but the measure does not reflect actual cessation. Given the lack of a significant difference between menthol and non-menthol smokers on either measure of quit attempts and the empirical uncertainty of the association between making a quit attempt or the number of quit attempts and actual cessation, there is no confident conclusion that can be drawn regarding an association with menthol smoking.

Pooled data for the meta-analyses were extracted for two outcome measures, quit attempts and abstinence. Pooled results from five studies suggested a significant association between menthol cigarette use and increased odds for past year quit attempts. However, pooled data from three studies measuring ever quit attempts found no difference between menthol and non-menthol smokers in the odds of making a quit attempt. Pooling data from all eight studies revealed no consistent differences.

Additional analysis of pooled data from two studies presenting results on quit attempts among Black participants showed that Black menthol, versus non-menthol, smokers were significantly more likely to make a quit attempt. Further, pooled data from three studies suggested that White menthol, versus non-menthol, smokers were significantly less likely of making a quit attempt.

Four cohort studies presented results for examining the association between menthol use and abstinence, with no specified duration. Pooled results showed no difference between menthol and non-menthol smokers in terms of abstinence, even in sub-analyses of Black and White participants, using data from three of the four studies.

Across all four cohort studies, pooled results on the association between menthol use and abstinence, again with no specified duration, showed no difference between menthol and non-menthol smokers, overall, in the odds of abstinence. However, when measuring abstinence by 7-day PPA, pooled data suggest that Black menthol smokers were significantly less likely than Black non-menthol smokers to be abstinent. Recognizing inconsistent results were reported across studies in the qualitative synthesis, meta-analytic results, generally, showed no difference between menthol cigarette use and quit attempts (pooled results from ever, past year quit attempts, any quit attempts between 2001 to 2005, and any quit attempt in the past 2, 3, or 5 years), abstinence with no defined duration, and 7-day PPA.

### Limitations

This systematic review was conducted according to established methodological standards and with inherent limitations. For example, the variation in the definitions of several outcome measures made it difficult to summarize results, which limited the reviewers’ ability to draw confident conclusions. Most of the smoking behavior data were self-reported. However, any differential impact of reliance on self-reported data was expected to be minimal. The Downs and Black checklist has some limitations when applied across a variety of study designs. Furthermore, a study’s quality score on the Downs and Black checklist may reflect the quality of reporting rather than the quality of the study as conducted. Finally, the conclusions in this review are based on studies conducted in the U.S. and may or may not be generalizable to other countries due to the potential impact of important influences, such as cultural norms, smoking policies, and taxes on smoking behaviors outside of the U.S.

## Conclusions

In summary, the findings of this systematic review suggest that the current evidence base is not strong or consistent enough to support a clear association—positive or negative—between menthol cigarette use and smoking cessation. Having comprehensively reviewed the available literature, this review—which included nearly three times the number of studies as the 2013 FDA Report and 2015 Addendum, including 16 studies that analyzed data among Black smokers only—recommends that future studies assessing the association between menthol cigarette smoking and smoking behaviors can be strengthened in several ways. Specifically, longitudinal data that measures cessation for 12 months or longer to reflect more sustained measures of cessation and adjusting for key demographic variables, at a minimum, will provide more insight into the potential association of menthol cigarette smoking and smoking cessation. Further, given the transparent, comprehensive, and objective approach taken in this review, it is the authors’ hope that these findings—as well as findings from their continued monitoring of the literature—will inform future policy decision-making, as well as influence the methodological approach of future systematic reviews towards an equivalent degree of strict methodological rigor.

## Supplementary Information

**Additional file 1.** Literature Search Strategy.

**Additional file 2.** Studies excluded at full-text level screening (with reason for exclusion).

**Additional file 3.** Outcome Measures for Smoking Cessation across Adjusted Studies.

**Additional file 4 **Evidence Table, Modeled / Adjusted Results (Duration of Abstinence, Any Quit Attempt, Number of Quit Attempts per Person, Rate of Abstinence/Quitting, Change in Smoking Quantity/Frequency, and Return to Smoking/Relapse) (*n* = 43 studies; *n* = 47 references).

**Additional file 5.** Sensitivity Analyses.

**Additional file 6.** Characteristics, Definitions, and Covariates of Studies Included in the Meta-Analysis.

## Data Availability

All data and materials considered in this review are publicly available.

## Abbreviations

AOR: Adjusted odds ratio
BRFSS: Behavioral Risk Factor Surveillance System
CARDIA: Coronary Artery Risk Development in Young Adults
CI: Confidence interval
COMMIT: Community Intervention Trial for Smoking Cessation
CPD: Cigarettes per day
CQ: Contextual Question
EPC: Evidence-Based Practice Center
HR: Hazard ratio
ITC-4: International Tobacco Control Four Country Survey
KIS: Kick it at Swope
KQ: Key Question
LYAC: Legacy Young Adult Cohort Study
NA: Not Applicable
NHIS: National Health Interview Survey
NHIS-CCS: National Health Interview Survey Cancer Control Supplement
PA: Prolonged abstinence
PATH: Population Assessment of Tobacco and Health
POR: Prevalence odds ratio
PPA: Point (or period) prevalence abstinence
PR: Prevalence ratio
SE: Standard error
SOE: Strength of Evidence
TUS-CPS: The Tobacco Use Supplement to the Current Population Survey
U.S.: United States
WSHS: Wisconsin Smokers Health Study
