# Supplementary material for: Assessing the evidence on the differential impact of menthol versus non-menthol cigarette use on smoking cessation in the U.S. population: a systematic review and meta-analysis
Source: Subst Abuse Treat Prev Policy. 2021 Aug 11;16:61. doi: 10.1186/s13011-021-00397-4 (PMC8359586; doi:10.1186/s13011-021-00397-4)
Supplement: Supplementary file 1 — Additional file 1. Literature Search Strategy. [file 13011_2021_397_MOESM1_ESM.docx]

**SUPPLEMENTAL SECTION 1: Literature Search Strategy**

**SUPPLEMENTAL SECTION 1: Literature Search Strategy**

**INITIAL DATABASE SEARCH – INCEPTION to DECEMBER 14, 2018**

Database(s): Cochrane Central Register of Controlled Trials; Cochrane Database of Systematic Reviews; Database of Abstracts of Reviews of Effects; MEDLINE; Embase; and PsycInfo

Search Strategy:

| **#** | **Searches** | **Results** |
| --- | --- | --- |
| 1 | (menthol* and (smok* or cigarette*)).mp. | 1,749 |
| 2 | limit 1 to english language [Limit not valid in CDSR,DARE; records were retained] | 1,675 |
| 3 | remove duplicates from 2 | 838 |

**INITIAL DATABASE SEARCH – JANUARY 01 2018 to JUNE 12 2020**

Database(s): Cochrane Central Register of Controlled Trials; Cochrane Database of Systematic Reviews; Database of Abstracts of Reviews of Effects; MEDLINE; Embase; and PsycInfo

Search Strategy:

| **#** | **Searches** | **Results** |
| --- | --- | --- |
| 1 | (menthol* and (smok* or cigarette*)).mp. | 2239 |
| 2 | limit 1 to english language [Limit not valid in CDSR; records were retained] | 2136 |
| 3 | limit 2 to yr="2018 -Current" | 683 |
| 4 | remove duplicates from 3 | 358 |
