# Supplementary material for: Assessing the evidence on the differential impact of menthol versus non-menthol cigarette use on smoking cessation in the U.S. population: a systematic review and meta-analysis
Source: Subst Abuse Treat Prev Policy. 2021 Aug 11;16:61. doi: 10.1186/s13011-021-00397-4 (PMC8359586; doi:10.1186/s13011-021-00397-4)
Supplement: Supplementary file 2 — Additional file 2. Studies excluded at full-text level screening (with reason for exclusion). [file 13011_2021_397_MOESM2_ESM.docx]

**SUPPLEMENTAL SECTION 2: Studies excluded at full-text level screening (with reason for exclusion)**

**SUPPLEMENTAL SECTION 2: Studies excluded at full-text level screening (with reason for exclusion)**

| **RefID** | **Reference** | **Exclusion Criteria** |
| --- | --- | --- |
| 11 | Centre for Reviews and Dissemination 2015 Aromatherapy: a systematic review (Structured abstract).. Database of Abstracts of Reviews of Effects; . | Does not address a KQ |
| 13 | Glantz S.A.,Gardiner P. 2018 Local movement to ban menthol tobacco products as a result of federal inaction. JAMA Internal Medicine; 178.711. | Does not address a KQ |
| 14 | Printz C. 2018 US Food and Drug Administration considers comments on proposed nicotine product regulations: Some criticize the agency for not moving fast enough to stop youth e-cigarette use. Cancer; 124.3959. | Ineligible exposure (e-cigarettes) |
| 17 | Stanfill S.B.,Croucher R.E.,Gupta P.C.,Lisko J.G.,Lawler T.S.,Kuklenyik P.,Dahiya M.,Duncan B.,Kimbrell J.B.,Peuchen E.H.,Watson C.H. 2018 Chemical characterization of smokeless tobacco products from South Asia: Nicotine, unprotonated nicotine, tobacco-specific N'-Nitrosamines, and flavor compounds. Food and Chemical Toxicology; 118.626. | Does not address a KQ |
| 20 | Schneller L.M.,McIntosh S.,Li D.,Rahman I.,Ossip D.,Goniewicz M.,O'Connor R.J. 2018 Tobacco use and chemosensory impairments among current adult tobacco users in the US: Data from NHANES 2013-2014. Tobacco Induced Diseases; 16.94202. | Does not address a KQ |
| 25 | Kong A.Y.,Golden S.D.,Berger M.T. 2018 An intersectional approach to the menthol cigarette problem: what's race(ism) got to do with it?. Critical Public Health; .1. | Does not address a KQ |
| 27 | Rath J.M.,Greenberg M.,Pitzer L.,Emelle B.,Green M.,Liu S.,Willett J.,Rose S.W.,Hair E.C.,Vallone D. 2018 The association between menthol perceptions and support for a policy ban among US smokers. Ethnicity and Disease; 28.177. | Does not address a KQ |
| 36 | White W.B. 2018 Menthol cigarette use and subclinical cardiovascular disease in african americans in the jackson heart study. Circulation; 137. | Does not address a KQ |
| 37 | McKelvey K.,Halpern-Felsher B.,Lazaro A. 2018 Youth-ascribed cigarette pack color meaning: Correct perceptions of green menthol flavor color, yet confusion over other colors. Journal of Adolescent Health; 62.S137. | Does not address a KQ |
| 42 | Folan P.,Abramova R.,Fardellone C.,Neptune E.,Sockrider M. 2017 Atient education, Information series: What is menthol?. American Journal of Respiratory and Critical Care Medicine; 196.P9. | Does not address a KQ |
| 43 | Farber H.J.,Folan P. 2017 The tobacco industry targets youth. American Journal of Respiratory and Critical Care Medicine; 196.11. | Does not address a KQ |
| 44 | Munafo M. 2016 Understanding the role of additives in tobacco products. Nicotine and Tobacco Research; 18.1545. | Does not address a KQ |
| 45 | Warner K.E.,Schroeder S.A. 2017 FDA's innovative plan to address the enormous toll of smoking. JAMA - Journal of the American Medical Association; 318.1755. | Does not address a KQ |
| 49 | Montgomery L.T. 2017 Mentholated cigarette use among African American women and men: What's blunt smoking got to do with it?. Drug and Alcohol Dependence; 171.e148. | Does not address a KQ |
| 59 | Anonymous. 2016 A growing gulf in the terrain of tobacco control. The Lancet; 387.2163. | Does not address a KQ |
| 60 | Meier E.,Vogel R.I.,O'Connor R.J.,Severson H.H.,Shields P.G.,Hatsukami D.K. 2016 Preference for flavored noncombustible nicotine products among smokers motivated to switch from cigarettes. Nicotine and Tobacco Research; 18.892. | Does not address a KQ |
| 62 | Munro H.M.,Tarone R.E.,Wang T.J.,Blot W.J. 2016 Menthol and nonmenthol cigarette smoking. Circulation; 133.1861. | Does not address a KQ |
| 69 | Mitchell K.A.,Zingone A.,Ryan B. 2016 Lung cancer in African Americans is characterized by histone modifying enzyme gene signatures. Cancer Epidemiology Biomarkers and Prevention; 25. | Does not address a KQ |
| 72 | Azagba S.,Minaker L.M.,Hammond D.,Manske S. 2015 Tobacco industry sponsored advocates have a different interpretation of science: a response to: Even anti-tobacco studies must be held to basic scientific standards. Cancer Causes and Control; 26.1363. | Does not address a KQ |
| 85 | Chirila C.,Trofor L.,Frunza A.,Albu A.M.,Gherghesanu R.,Trofor A.C. 2015 The problem of low rate-smokers: Smoking pattern and cessation outcomes. European Respiratory Journal; 46. | Does not address a KQ |
| 93 | Suh Y.,Lee Y.,Lee H.,Kim S.,Choe Y.,Park S.,Bowler R.P. 2015 Relationship of menthol cigarett smoking with COPD, comorbidities, and CT metrics. American Journal of Respiratory and Critical Care Medicine; 191. | Does not address a KQ |
| 94 | Pratt-Chapman M.,Hatcher E.,Brooks J.,Davies M. 2015 Tailoring tobacco cessation interventions for special populations served by federally qualified health centers. Psycho-Oncology; 24.50. | Does not address a KQ |
| 95 | Paschke M.,Hutzler C.,Tkachenko A.,Henkler F.,Luch A. 2015 Effect of the menthol content of cigarettes on the cold-menthol receptor TRPM8. Naunyn-Schmiedeberg's Archives of Pharmacology; 388.S78. | Does not address a KQ |
| 101 | Cunningham R. 2014 Prohibitions/restrictions on flavoured tobacco products: Updated overview of international developments. Asia-Pacific Journal of Clinical Oncology; 10.182. | Does not address a KQ |
| 110 | Schneider M.E. 2013 ASCO issues to-do list on initiatives for tobacco control. Oncology Report; .15. | Does not address a KQ |
| 113 | Alabi T. 2013 Menthol cigarette use among African Americans. Drug Topics; .4. | Not original research / data |
| 114 | McKee M. 2013 European Union's tobacco products directive: Many questions remain about the influence of industry. BMJ (Online); 347.f6196. | Ineligible population (not U.S., not humans) |
| 122 | King A.,Zhang L.,Roche D.,Cao D.,Tyndale R. 2012 Nicotine metabolism: Sex differences in African-American and Caucasian smokers. Neuropsychopharmacology; 38.S419. | Does not address a KQ |
| 123 | Uhl G.,Walther D.,Behm F.,Rose J. 2012 Trpa1 ''menthol preference'' haplotypes are associated with levels of trpa1 expression and smoking cessation success. Neuropsychopharmacology; 38.S212. | Does not address a KQ |
| 125 | Vozoris N.T. 2012 The association between mentholated cigarettes and cardiovascular and pulmonary diseases in the United States population. American Journal of Respiratory and Critical Care Medicine; 185. | Does not address a KQ |
| 128 | Bethea T.N.,Rosenberg L.,Boggs D.A.,Palmer J.R. 2012 Menthol cigarettes in relation to breast cancer incidence in African American women. Cancer Research; 72. | Does not address a KQ |
| 129 | Jones M.R.,Apelberg B.J.,Navas-Acien A. 2012 Smoking, menthol cigarettes and peripheral arterial disease in the 1999- 2004 national health and nutrition examination survey (nhanes). Circulation; 125. | Does not address a KQ |
| 134 | Thomas K.V.,Reid M.J. 2011 What else can the analysis of sewage for urinary biomarkers reveal about communities?. Environmental Science and Technology; 45.7611. | Does not address a KQ |
| 140 | Backinger C.L. 2011 Tobacco-related disparities and the Family Smoking Prevention and Tobacco Control Act. Cancer Epidemiology Biomarkers and Prevention; 20. | Does not address a KQ |
| 141 | Stellman S.D. 2011 Tobacco-related cancer risks and menthol cigarettes: A case-control study. American Journal of Epidemiology; 173. | Does not address a KQ |
| 143 | Brody A.L.,Mukhin A.G.,La Charite J.,Ta K.,Farahi J.,Sugar C.A.,Vellios E.,Archie M.,Kozman M.,Mandelkern M.A. 2011 Up-regulation of nicotinic acetylcholine receptors in menthol cigarette smokers. Neuropsychopharmacology; 36.S402. | Does not address a KQ |
| 144 | Buchanan T.S.,Cox L.S.,Nollen N.L.,Thomas J.L.,Berg C.J.,Mayo M.S.,Ahluwalia J.S. 2011 Perceived treatment assignment and smoking cessation in a clinical trial of bupropion. Cancer Epidemiology Biomarkers and Prevention; 20.721. | Does not address a KQ |
| 146 | Mickens L.,Ameringer K.,Brightman M.,Leventhal A.M. 2010 Epidemiology, determinants, and consequences of cigarette smoking in African American women: An integrative review. Addictive Behaviors; 35.383. | Does not address a KQ |
| 148 | Shah L.,Press V.,Arora V.,King A.,Meltzer D.,Suresh K.,Krishnan J. 2010 Assessing impact of usual care on quit rates 4 weeks postdischarge among hospitalized smokers. Journal of Hospital Medicine; 5.71. | Does not address a KQ |
| 149 | Fu S.,Stahre M.,Okuyemi K.,Joseph A. 2010 Racial/ethnic differences in menthol cigarette smoking, prevalence of smoking cessation, and utilization of evidence-based tobacco cessation treatments. Journal of General Internal Medicine; 25.S377. | Duplicate article |
| 151 | Wigand J. 2009 Smoking and mental illness: A commentary and counterpoint. Journal of Dual Diagnosis; 5.219. | Does not address a KQ |
| 152 | Gough N.R. 2009 Nicotine irritation. Science Signaling; 2.ec324. | Does not address a KQ |
| 153 | Anonymous. 2009 Correction: Article on exposure biomarkers of menthol and nonmenthol smoking (Cancer Epidemiology, Biomarkers and Prevention (2009)). Cancer Epidemiology Biomarkers and Prevention; 18.2155. | Not original research / data |
| 155 | Peace J.,Wilson N.,Thomson G.,Edwards R. 2008 Recent changes in cigarette packaging in New Zealand may continue to mislead smokers [9]. New Zealand Medical Journal; 121. | Ineligible population (not U.S., not humans) |
| 158 | Hebert R. 2008 What's new in nicotine & tobacco research?. Nicotine and Tobacco Research; 10.559. | Not original research / data |
| 159 | Marshall S. 2008 Giving advice on sore throats. Pharmaceutical Journal; 280.127. | Does not address a KQ |
| 161 | Hebert R. 2008 What's new in nicotine & tobacco research?. Nicotine and Tobacco Research; 10.1671. | Duplicate article |
| 162 | Marshall S. 2007 Over-the-counter advice for coughs. Pharmaceutical Journal; 278.85. | Does not address a KQ |
| 163 | Anonymous. 2007 Contents and design features of tobacco products: Their relationship to dependence potential and consumer appeal. The Scientific Basis of Tobacco Product Regulation,World Health Organization - Technical Report Series; .7. | Does not address a KQ |
| 164 | Hebert R. 2007 What's new in Nicotine & Tobacco Research?. Nicotine and Tobacco Research; 9.787. | Not original research / data |
| 165 | Tidey J.W.,Williams J. 2007 Clinical indices of tobacco use in people with schizophrenia. Journal of Dual Diagnosis; 3.79. | Does not address a KQ |
| 166 | Foulds J.,Williams J.M.,Gandhi K.K. 2006 To the editor [5]. New England Journal of Medicine; 354.1952. | Not original research / data |
| 167 | Morice A.H.,McGarvey L.,Pavord I. 2006 Recommendations for the management of cough in adults. Thorax; 61. | Does not address a KQ |
| 168 | Hebert R. 2005 What's new in nicotine & tobacco research?. Nicotine and Tobacco Research; 7.485. | Not original research / data |
| 170 | Hebert R. 2004 What's new in Nicotine & Tobacco Research?. Nicotine and Tobacco Research; 6.S279. | Not original research / data |
| 171 | Castro F.G. 2004 Erratum: "Physiological, psychological, social, and cultural influences on the use of menthol cigarettes among Blacks and Hispanics" (Nicotine & Tobacco Research (2004) (S30-S43)). Nicotine and Tobacco Research; 6.575. | Does not address a KQ |
| 173 | Gonzalez Castro F. 2004 Physiological, psychological, social, and cultural influences on the use of menthol cigarettes among Blacks and Hispanics. Nicotine and Tobacco Research; 6.S29. | Not original research / data |
| 174 | Sutton C.D.,Robinson R.G. 2004 The marketing of menthol cigarettes in the United States: Population, messages, and channels. Nicotine and Tobacco Research; 6.S83. | Does not address a KQ |
| 176 | Brandwein-Gensler M.,Hille J.J. 2003 Behind the cover: The guthka story. Archives of Otolaryngology - Head and Neck Surgery; 129.699. | Does not address a KQ |
| 177 | Hebert J.R. 2003 Invited commentary: Menthol cigarettes and risk of lung cancer. American Journal of Epidemiology; 158.617. | Does not address a KQ |
| 181 | Pickworth W.B.,Moolchan E.T.,Berlin I.,Murty R. 2002 Sensory and physiologic effects of menthol and nonmenthol cigarettes with differing nicotine delivery. Pharmacology Biochemistry and Behavior; 71.55. | Does not address a KQ |
| 182 | Hebert R. 2002 What's new in Nicotine & Tobacco Research?. Nicotine and Tobacco Research; 4.385. | Does not address a KQ |
| 186 | Newton G.D.,Pray W.S.,Popovich N.G. 1997 New OTC drugs and devices: A selective review. As the array of OTC drugs diversifies, pharmacists should be prepared to guide patients in selection and use of these products. Journal of the American Pharmaceutical Association; 37.165. | Does not address a KQ |
| 188 | Anonymous. 1995 Minerva. British Medical Journal; 310.1080. | Does not address a KQ |
| 189 | Royce J.M.,Hymowitz N.,Corbett K.,Hartwell T.D.,Orlandi M.A. 1993 Smoking cessation factors among African Americans and Whites. American Journal of Public Health; 83.220. | Does not address a KQ |
| 190 | Sidney S.,Tekawa I.,Friedman G.D. 1989 Metholated cigarette use among multiphasic examinees, 1979-86. American Journal of Public Health; 79.1415. | Does not address a KQ |
| 191 | McCarthy W.J.,Caskey N.H.,Jarvik M.E.,Perez-Stable E.J.,Marin B.,Marin G.,Benowitz N.,Wagenknecht L.E.,Haley N.J.,Jacobs Jr. D.R. 1992 Ethnic differences in nicotine exposure [1]. American Journal of Public Health; 82.1171. | Not original research / data |
| 192 | Kabat G.C.,Morabia A.,Wynder E.L. 1991 Comparison of smoking habits of Blacks and Whites in a case-control study. American Journal of Public Health; 81.1483. | Does not address a KQ |
| 194 | Hill R.M.,Tennyson L.M. 1985 The effect of maternal allergy medications on the fetus. Immunology and Allergy Practice; 7.80. | Does not address a KQ |
| 197 | Fisher A.A. 1978 Reports from the second international contact dermatitis symposium. Part III. Cutis; 22.272. | Does not address a KQ |
| 198 | Ziment I. 1974 Mucokinesis the methodology of moving mucus. #journal#; 4.15. | Does not address a KQ |
| 200 | Lauren R Pacek, Jason A Oliver, Maggie M Sweitzer, F Joseph McClernon 2018 Young adult dual combusted cigarette and e-cigarette users' anticipated responses to a nicotine reduction policy and menthol ban in combusted cigarettes.. Drug and alcohol dependence; 194.40. | Does not address a KQ |
| 202 | Guy Jaccard, Maxim Belushkin, Cyril Jeannet, Evi Nurlaili Aldilla, Andry Ongko Wijoyo 2018 Investigation of menthol content and transfer rates in cigarettes and Tobacco Heating System 2.2.. Regulatory toxicology and pharmacology : RTP; 101.48. | Does not address a KQ |
| 206 | John Buckell, Joachim Marti, Jody L Sindelar 2018 Should flavours be banned in cigarettes and e-cigarettes? Evidence on adult smokers and recent quitters from a discrete choice experiment.. Tobacco control; . | Does not address a KQ |
| 207 | Yvette van der Eijk, Jeong Kyu Lee, Pamela Ling 2018 How Menthol Is Key to the Tobacco Industry's Strategy of Recruiting and Retaining Young Smokers in Singapore.. The Journal of adolescent health : official publication of the Society for Adolescent Medicine; . | Ineligible population (not U.S., not humans) |
| 208 | Hongying Dai, Jianqiang Hao 2018 Flavored Tobacco Use Among U.S. Adults by Age Group: 2013-2014.. Substance use & misuse; .1. | Does not address a KQ |
| 210 | Amanda Y Kong, Shelley D Golden, Allison E Myers, Melissa A Little, Robert Klesges, Wayne Talcott, Sara M Vandegrift, Daniel G Cassidy, Kurt M Ribisl 2018 Availability, price and promotions for cigarettes and non-cigarette tobacco products: an observational comparison of US Air Force bases with nearby tobacco retailers, 2016.. Tobacco control; . | Does not address a KQ |
| 215 | Adolfo G Cuevas, Kasim Ortiz, Nancy Lopez, David R Williams 2018 Assessing racial differences in lifetime and current smoking status & menthol consumption among Latinos in a nationally representative sample.. Ethnicity & health; .1. | Does not address a KQ |
| 217 | Mary J Sandage, Rita Patel 2018 Passive Upper Airway Thermoregulation and High-Speed Assessment for Conventional versus Menthol Cigarette: Implications for Laryngeal Physiology.. Journal of voice : official journal of the Voice Foundation; . | Does not address a KQ |
| 218 | Lauren Czaplicki, Joanna E Cohen, Miranda R Jones, Katherine Clegg Smith, Lainie Rutkow, Jill Owczarzak 2018 Compliance with the City of Chicago's partial ban on menthol cigarette sales.. Tobacco control; . | Does not address a KQ |
| 219 | Karen Ahijevych, Laura Szalacha, Alai Tan 2018 Effects of menthol flavor cigarettes or total urinary menthol on biomarkers of nicotine and carcinogenic exposure and behavioral measures.. Nicotine & tobacco research : official journal of the Society for Research on Nicotine and Tobacco; . | Does not address a KQ |
| 225 | Erna J Z Krusemann, Marlou P Lasschuijt, C de Graaf, Rene A de Wijk, Pieter H Punter, Loes van Tiel, Johannes W J M Cremers, Suzanne van de Nobelen, Sanne Boesveldt, Reinskje Talhout 2018 Sensory analysis of characterising flavours: evaluating tobacco product odours using an expert panel.. Tobacco control; . | Does not address a KQ |
| 227 | Mary F Brunette, Joelle C Ferron, Pamela Geiger, Andrea C Villanti 2018 Menthol Cigarette use in Young Adult Smokers with Severe Mental Illnesses.. Nicotine & tobacco research : official journal of the Society for Research on Nicotine and Tobacco; . | Does not address a KQ |
| 228 | Amy M Cohn, Shyanika W Rose, Vinu Ilakkuvan, Tiffany Gray, Laurel Curry, Andrea C Villanti, Darren Mays, Eric Lindblom, Kenneth Tercyak, Charles Debnam, Ashley Mayo, Lexie Perreras 2018 Harm Perceptions of Menthol and Nonmenthol Cigarettes Differ by Brand, Race/Ethnicity, and Gender in US Adult Smokers: Results from PATH Wave 1.. Nicotine & tobacco research : official journal of the Society for Research on Nicotine and Tobacco; . | Does not address a KQ |
| 233 | Jiu Ai, Kenneth M Taylor, Joseph G Lisko, Hang Tran, Clifford H Watson, Matthew R Holman 2018 Menthol levels in cigarettes from eight manufacturers.. Tobacco control; 27.335. | Does not address a KQ |
| 237 | Anna Pagano, Noah R Gubner, Thao Le, Deborah Yip, Denise Williams, Kevin Delucchi, Joseph Guydish 2018 Differences in tobacco use prevalence, behaviors, and cessation services by race/ethnicity: A survey of persons in addiction treatment.. Journal of substance abuse treatment; 94.9. | Does not address a KQ |
| 240 | Hyung Soo Kim, Eun Chul Pack, Ye Ji Koo, Ye Jin Lee, Dae Kwan Sung, Seung Ha Lee, Young Soon Kim, Kyeng Hee Kwon, Kyung Min Lim, Dae Yong Jang, Dal Woong Choi 2018 Corrigendum to "Quantitative analysis of menthol and identification of other flavoring ingredients in capsule cigarettes marketed in Korea" [Regul. Toxicol. Pharmacol. 92C (2018) 420-428].. Regulatory toxicology and pharmacology : RTP; 95.448. | Does not address a KQ |
| 241 | Andrea C Villanti, Diann E Gaalema, Jennifer W Tidey, Allison N Kurti, Stacey C Sigmon, Stephen T Higgins 2018 Co-occurring vulnerabilities and menthol use in U.S. young adult cigarette smokers: Findings from Wave 1 of the PATH Study, 2013-2014.. Preventive medicine; 117.43. | Does not address a KQ |
| 243 | Eric Craig Leas, John P Pierce, Claudiu V Dimofte, Dennis R Trinidad, David R Strong 2018 Standardised cigarette packaging may reduce the implied safety of Natural American Spirit cigarettes.. Tobacco control; 27.e118. | Does not address a KQ |
| 245 | Sarah D Mills, Lisa Henriksen, Shelley D Golden, Rachel Kurtzman, Amanda Y Kong, Tara L Queen, Kurt M Ribisl 2018 Disparities in retail marketing for menthol cigarettes in the United States, 2015.. Health & place; 53.62. | Does not address a KQ |
| 249 | Mark G LeSage, John R Smethells, Andrew C Harris 2018 Status and Future Directions of Preclinical Behavioral Pharmacology in Tobacco Regulatory Science.. Behavior analysis (Washington, D.C.); 18.252. | Does not address a KQ |
| 251 | Phoenix Alicia Matthews, Amanda C Blok, Joseph G L Lee, Brian Hitsman, Lisa Sanchez-Johnsen, Karriem Watson, Elizabeth Breen, Raymond Ruiz,Scout, Melissa A Simon, Marian Fitzgibbon, Laura C Hein, Robert Winn 2018 SBM recommends policy support to reduce smoking disparities for sexual and gender minorities.. Translational behavioral medicine; 8.692. | Does not address a KQ |
| 252 | Anna Pagano, Noah Gubner, Thao Le, Joseph Guydish 2018 Cigarette smoking and quit attempts among Latinos in substance use disorder treatment. The American journal of drug and alcohol abuse; 44.660. | Does not address a KQ |
| 253 | Saul Shiffman, Sarah Scholl 2018 Increases in Cigarette Consumption and Decreases in Smoking Intensity When Nondaily Smokers Are Provided With Free Cigarettes.. Nicotine & tobacco research : official journal of the Society for Research on Nicotine and Tobacco; 20.1237. | Does not address a KQ |
| 258 | Joanne D'Silva, Amy M Cohn, Amanda L Johnson, Andrea C Villanti 2018 Differences in Subjective Experiences to First Use of Menthol and Nonmenthol Cigarettes in a National Sample of Young Adult Cigarette Smokers.. Nicotine & tobacco research : official journal of the Society for Research on Nicotine and Tobacco; 20.1062. | Does not address a KQ |
| 259 | Olivia A Wackowski, Kiameesha R Evans, Melissa B Harrell, Alexandra Loukas, M Jane Lewis, Cristine D Delnevo, Cheryl L Perry 2018 In Their Own Words: Young Adults' Menthol Cigarette Initiation, Perceptions, Experiences and Regulation Perspectives.. Nicotine & tobacco research : official journal of the Society for Research on Nicotine and Tobacco; 20.1076. | Does not address a KQ |
| 262 | Jessica M Rath, Marisa Greenberg, Lindsay Pitzer, Brittany Emelle, Molly Green, Shiyang Michael Liu, Jeffrey Willett, Shyanika W Rose, Elizabeth C Hair, Donna Vallone 2018 The Association Between Menthol Perceptions and Support for a Policy Ban Among US Smokers.. Ethnicity & disease; 28.177. | Does not address a KQ |
| 264 | Monica Webb Hooper 2018 Editorial: Preventing Tobacco-Related Cancer Disparities: A Focus on Racial/Ethnic Minority Populations.. Ethnicity & disease; 28.129. | Does not address a KQ |
| 267 | Jessica L Fetterman, Robert M Weisbrod, Bihua Feng, Reena Bastin, Shawn T Tuttle, Monica Holbrook, Gregory Baker, Rose Marie Robertson, Daniel J Conklin, Aruni Bhatnagar, Naomi M Hamburg 2018 Flavorings in Tobacco Products Induce Endothelial Cell Dysfunction.. Arteriosclerosis, thrombosis, and vascular biology; 38.1607. | Does not address a KQ |
| 270 | Allison J Lazard, Sarah D Kowitt, Li-Ling Huang, Seth M Noar, Kristen L Jarman, Adam O Goldstein 2018 Believability of Cigarette Warnings About Addiction: National Experiments of Adolescents and Adults.. Nicotine & tobacco research : official journal of the Society for Research on Nicotine and Tobacco; 20.867. | Does not address a KQ |
| 271 | Nicole M Kuiper, Doris Gammon, Brett Loomis, Kyle Falvey, Teresa W Wang, Brian A King, Todd Rogers 2018 Trends in Sales of Flavored and Menthol Tobacco Products in the United States During 2011-2015.. Nicotine & tobacco research : official journal of the Society for Research on Nicotine and Tobacco; 20.698. | Does not address a KQ |
| 273 | Michael Chaiton, Robert Schwartz, Joanna E Cohen, Eric Soule, Thomas Eissenberg 2018 Association of Ontario's Ban on Menthol Cigarettes With Smoking Behavior 1 Month After Implementation.. JAMA internal medicine; 178.710. | Does not address a KQ |
| 274 | Cendrine D Robinson, Christine Muench, Emily Brede, Romano Endrighi, Edwin H Szeto, Joanna R Sells, John P Lammers, Kolawole S Okuyemi, Grant Izmirlian, Andrew J Waters 2018 Pro-tobacco advertisement exposure among African American smokers: An ecological momentary assessment study.. Addictive behaviors; 83.142. | Does not address a KQ |
| 275 | Julia Gerharz, Michael H K Bendels, Markus Braun, Doris Klingelhofer, David A Groneberg, Ruth Mueller 2018 Particulate matter emissions of different brands of mentholated cigarettes.. Journal of the Air & Waste Management Association (1995); 68.608. | Does not address a KQ |
| 277 | Alyssa Marie M Antonio, Pebbles Fagan, Faith D Hamamura, Ian Joseph N Lagua, Jenny Liu, Devin J Park, Pallav Pokhrel, Thaddeus A Herzog, Ian Pagano, Kevin Cassel, Angela Sy, Dorothy Jorgensen, Tania Lynch, Crissy Kawamoto, Carol J Boushey, Adrian Franke, Mark S Clanton, Eric T Moolchan, Linda A Alexander 2015 Menthol cigarette smoking and obesity in young adult daily smokers in Hawaii.. Preventive medicine reports; 2.946. | Does not address a KQ |
| 282 | Peter Jatlow, Gerald Valentine, Ralitza Gueorguieva, Haleh Nadim, Ran Wu, Stephanie S O'Malley, Mehmet Sofuoglu 2018 Plasma Menthol Glucuronide as a Biomarker of Acute Menthol Inhalation.. Tobacco regulatory science; 4.586. | Does not address a KQ |
| 284 | Tameka S Lawler, Stephen B Stanfill, B Rey deCastro, Joseph G Lisko, Bryce W Duncan, Patricia Richter, Clifford H Watson 2017 Surveillance of Nicotine and pH in Cigarette and Cigar Filler.. Tobacco regulatory science; 3.101. | Does not address a KQ |
| 286 | Kenneth A Perkins, Joshua L Karelitz, Nicole Kunkle 2018 Evaluation of menthol per se on acute perceptions and behavioral choice of cigarettes differing in nicotine content.. Journal of psychopharmacology (Oxford, England); 32.324. | Does not address a KQ |
| 289 | Gillian L Schauer, Erica N Peters, Zachary R Rosenberry, Hyoshin Kim 2018 Trends in and Characteristics of Marijuana and Menthol Cigarette Use Among Current Cigarette Smokers, 2005-2014.. Nicotine & tobacco research : official journal of the Society for Research on Nicotine and Tobacco; 20.362. | Does not address a KQ |
| 290 | Philip H Smith, Ezinne Akpara, Raisa Haq, Mark El-Miniawi, Azure B Thompson 2017 Gender and Menthol Cigarette Use in the United States: A Systematic Review of the Recent Literature (2011 - May 2017).. Current addiction reports; 4.431. | Does not address a KQ |
| 293 | Patricia Richter, Pappas R Steven, Roberto Bravo, Joseph G Lisko, Maria Damian, Nathalie Gonzalez-Jimenez, Naudia Gray, Lisa M Keong, Jacob B Kimbrell, Peter Kuklenyik, Tameka S Lawler, Grace E Lee, Magaly Mendez, Jose Perez, Shakia Smith, Hang Tran, Robert Tyx, Clifford H Watson 2016 Characterization of SPECTRUM Variable Nicotine Research Cigarettes.. Tobacco regulatory science; 2.94. | Does not address a KQ |
| 295 | Amy Cohn, Amanda Johnson, Jennifer Pearson, Shyanika Rose, Sarah Ehlke, Ollie Ganz, Raymond Niaura 2017 Determining non-cigarette tobacco, alcohol, and substance use typologies across menthol and non-menthol smokers using latent class analysis.. Tobacco induced diseases; 15.5. | Does not address a KQ |
| 296 | Amy M Cohn, Amanda L Johnson, Elizabeth Hair, Jessica M Rath, Andrea C Villanti 2016 Menthol tobacco use is correlated with mental health symptoms in a national sample of young adults: implications for future health risks and policy recommendations.. Tobacco induced diseases; 14.1. | Does not address a KQ |
| 299 | Allison C Hoffman 2011 The health effects of menthol cigarettes as compared to non-menthol cigarettes.. Tobacco induced diseases; 9 Suppl 1.S7. | Does not address a KQ |
| 300 | Allison C Hoffman, Donna Miceli 2011 Menthol cigarettes and smoking cessation behavior.. Tobacco induced diseases; 9 Suppl 1.S6. | Not original research / data |
| 301 | Allison C Hoffman, Dee Simmons 2011 Menthol cigarette smoking and nicotine dependence.. Tobacco induced diseases; 9 Suppl 1.S5. | Not original research / data |
| 302 | Joshua Rising, Kristina Wasson-Blader 2011 Menthol and initiation of cigarette smoking.. Tobacco induced diseases; 9 Suppl 1.S4. | Not original research / data |
| 303 | Deirdre Lawrence, Brie Cadman, Allison C Hoffman 2011 Sensory properties of menthol and smoking topography.. Tobacco induced diseases; 9 Suppl 1.S3. | Not original research / data |
| 306 | Allison C Hoffman 2011 Introduction: Mentholated cigarettes and public health.. Tobacco induced diseases; 9 Suppl 1.I1. | Does not address a KQ |
| 311 | Eva O Hansen, Lars Arendt-Nielsen, Shellie A Boudreau 2017 A Comparison of Oral Sensory Effects of Three TRPA1 Agonists in Young Adult Smokers and Non-smokers.. Frontiers in physiology; 8.663. | Does not address a KQ |
| 313 | An-Hsuan Lin, Meng-Han Liu, Hsin-Kuo B Ko, Diahn-Warng Perng, Tzong-Shyuan Lee, Yu Ru Kou 2017 Inflammatory Effects of Menthol vs. Non-menthol Cigarette Smoke Extract on Human Lung Epithelial Cells: A Double-Hit on TRPM8 by Reactive Oxygen Species and Menthol.. Frontiers in physiology; 8.263. | Does not address a KQ |
| 314 | Cynthia Van Landingham, William Fuller, Greg Mariano, Kristin Marano, Geoffrey Curtin, Sandra I Sulsky 2017 Data on cardiovascular and pulmonary diseases among smokers of menthol and non-menthol cigarettes compiled from the National Health and Nutrition Examination Survey (NHANES), 1999-2012.. Data in brief; 12.386. | Does not address a KQ |
| 320 | Jeff Stier 2015 Even anti-tobacco studies must be held to basic scientific standards. A response to: Smoking intensity and intent to continue smoking among menthol and non-menthol adolescent smokers in Canada.. Cancer causes & control : CCC; 26.1053. | Ineligible population (not U.S., not humans) |
| 322 | Md Mizanur Rahman, Mohd Taha Arif, Mohd Fadzillah Abd Razak, Mohd Raili Bin Suhaili, Zainab Tambi, Clifton Akoi, Deburra Peak Ngadan 2014 Does menthol-brand cigarette initiate early smoking? Evidence from a cross sectional study in sarawak, malaysia.. Iranian journal of public health; 43.385. | Ineligible population (not U.S., not humans) |
| 324 | Nadine Kabbani 2013 Not so Cool? Menthol's discovered actions on the nicotinic receptor and its implications for nicotine addiction.. Frontiers in pharmacology; 4.95. | Does not address a KQ |
| 327 | Allison M Schmidt, Sarah D Kowitt, Allison E Myers, Adam O Goldstein 2018 Attitudes towards Potential New Tobacco Control Regulations among U.S. Adults.. International journal of environmental research and public health; 15. | Does not address a KQ |
| 329 | Ruth E Malone 2017 It's the 21st century: isn't it past time to ban menthol cigarette sales?.. Tobacco control; 26.359. | Does not address a KQ |
| 335 | Jennifer Brown, Teresa DeAtley, Kevin Welding, Robert Schwartz, Michael Chaiton, Deirdre Lawrence Kittner, Joanna E Cohen 2017 Tobacco industry response to menthol cigarette bans in Alberta and Nova Scotia, Canada.. Tobacco control; 26.e71. | Does not address a KQ |
| 340 | Mathilde Marchand, Patrick Brossard, Henri Merdjan, Nicola Lama, Rolf Weitkunat, Frank Ludicke 2017 Nicotine Population Pharmacokinetics in Healthy Adult Smokers: A Retrospective Analysis.. European journal of drug metabolism and pharmacokinetics; 42.943. | Does not address a KQ |
| 341 | Jessica M Rath, Andrea C Villanti, Valerie F Williams, Amanda Richardson, Jennifer L Pearson, Donna M Vallone 2016 Correlates of current menthol cigarette and flavored other tobacco product use among U.S. young adults.. Addictive behaviors; 62.35. | Does not address a KQ |
| 342 | Brandon J Henderson, Teagan R Wall, Beverley M Henley, Charlene H Kim, Sheri McKinney, Henry A Lester 2017 Menthol Enhances Nicotine Reward-Related Behavior by Potentiating Nicotine-Induced Changes in nAChR Function, nAChR Upregulation, and DA Neuron Excitability.. Neuropsychopharmacology : official publication of the American College of Neuropsychopharmacology; 42.2285. | Does not address a KQ |
| 346 | Andrea C Villanti, Lauren K Collins, Raymond S Niaura, Stacey Y Gagosian, David B Abrams 2017 Menthol cigarettes and the public health standard: a systematic review.. BMC public health; 17.983. | Does not address a KQ |
| 347 | Christina Vaughan Watson, Patricia Richter, B Rey de Castro, Connie Sosnoff, Jennifer Potts, Pamela Clark, Joan McCraw, Xizheng Yan, David Chambers, Clifford Watson 2017 Smoking Behavior and Exposure: Results of a Menthol Cigarette Cross-over Study.. American journal of health behavior; 41.309. | Does not address a KQ |
| 348 | Kathryn C Ross, Delia A Dempsey, Gideon St Helen, Kevin Delucchi, Neal L Benowitz 2016 The Influence of Puff Characteristics, Nicotine Dependence, and Rate of Nicotine Metabolism on Daily Nicotine Exposure in African American Smokers.. Cancer epidemiology, biomarkers & prevention : a publication of the American Association for Cancer Research, cosponsored by the American Society of Preventive Oncology; 25.936. | Does not address a KQ |
| 350 | Won S Choi, Niaman Nazir, Christina M Pacheco, Melissa K Filippi, Joseph Pacheco, Julia White Bull, Christi Nance, Babalola Faseru, K Allen Greiner, Christine Makosky Daley 2016 Recruitment and Baseline Characteristics of American Indian Tribal College Students Participating in a Tribal College Tobacco and Behavioral Survey.. Nicotine & tobacco research : official journal of the Society for Research on Nicotine and Tobacco; 18.1488. | Does not address a KQ |
| 351 | Erna Jz Krusemann, Wouter F Visser, Johannes Wjm Cremers, Jeroen LA Pennings, Reinskje Talhout 2018 Identification of flavour additives in tobacco products to develop a flavour library.. Tobacco control; 27.105. | Does not address a KQ |
| 353 | Erna J Z Krusemann, Johannes W J M Cremers, Wouter F Visser, Pieter H Punter, Reinskje Talhout 2017 The Sensory Difference Threshold of Menthol Odor in Flavored Tobacco Determined by Combining Sensory and Chemical Analysis.. Chemical senses; 42.233. | Does not address a KQ |
| 355 | Shyanika W Rose, Catherine L Jo, Steven Binns, Melissa Buenger, Sherry Emery, Kurt M Ribisl 2017 Perceptions of Menthol Cigarettes Among Twitter Users: Content and Sentiment Analysis.. Journal of medical Internet research; 19.e56. | Does not address a KQ |
| 356 | Natalie Nardone,​ Eric C Donny,​ Dorothy K Hatsukami,​ Joseph S Koopmeiners,​ Sharon E Murphy,​ Andrew A Strasser,​ Jennifer W Tidey,​ Ryan Vandrey,​ Neal L Benowitz 2016 Estimations and predictors of non-compliance in switchers to reduced nicotine content cigarettes.. Addiction (Abingdon,​ England); 111.2208. | Does not address a KQ |
| 357 | Elizabeth Needham Waddell, Rachel Sacks, Shannon M Farley, Michael Johns 2016 Point-of-Sale Tobacco Marketing to Youth in New York State.. The Journal of adolescent health : official publication of the Society for Adolescent Medicine; 59.365. | Does not address a KQ |
| 360 | Shannon M Farley, Michael Johns 2017 New York City flavoured tobacco product sales ban evaluation.. Tobacco control; 26.78. | Does not address a KQ |
| 367 | Andrea C Villanti, Amanda L Johnson, Bridget K Ambrose, K Michael Cummings, Cassandra A Stanton, Shyanika W Rose, Shari P Feirman, Cindy Tworek, Allison M Glasser, Jennifer L Pearson, Amy M Cohn, Kevin P Conway, Raymond S Niaura, Maansi Bansal-Travers, Andrew Hyland 2017 Flavored Tobacco Product Use in Youth and Adults: Findings From the First Wave of the PATH Study (2013-2014).. American journal of preventive medicine; 53.139. | Does not address a KQ |
| 369 | K Sterling, C Fryer, I Pagano, D Jones, P Fagan 2016 Association between menthol-flavoured cigarette smoking and flavoured little cigar and cigarillo use among African-American, Hispanic, and white young and middle-aged adult smokers.. Tobacco control; 25.ii21. | Does not address a KQ |
| 376 | P Clemmey, R Brooner, M A Chutuape, M Kidorf, M Stitzer 1997 Smoking habits and attitudes in a methadone maintenance treatment population.. Drug and alcohol dependence; 44.123. | Does not address a KQ |
| 379 | Nancy C Jao, Anna K Veluz-Wilkins, Matthew J Smith, Allison J Carroll, Sonja Blazekovic, Frank T Leone, Rachel F Tyndale, Robert A Schnoll, Brian Hitsman 2017 Does menthol cigarette use moderate the effect of nicotine metabolism on short-term smoking cessation?.. Experimental and clinical psychopharmacology; 25.216. | Does not address a KQ |
| 380 | N H Caskey, M E Jarvik, W J McCarthy, M R Rosenblatt, T M Gross, C L Carpenter 1993 Rapid smoking of menthol and nonmenthol cigarettes by black and white smokers.. Pharmacology, biochemistry, and behavior; 46.259. | Does not address a KQ |
| 385 | Pamela Valera, F Joseph McClernon, Greer Burkholder, Michael J Mugavero, James Willig, Conall O'Cleirigh, Karen L Cropsey 2017 A Pilot Trial Examining African American and White Responses to Algorithm-Guided Smoking Cessation Medication Selection in Persons Living with HIV.. AIDS and behavior; 21.1975. | Does not address a KQ |
| 387 | Charles J Courtemanche, Makayla K Palmer, Michael F Pesko 2017 Influence of the Flavored Cigarette Ban on Adolescent Tobacco Use.. American journal of preventive medicine; 52.e139. | Does not address a KQ |
| 389 | Arnab Mukherjea, Olivia A Wackowski, Youn Ok Lee, Cristine D Delnevo 2014 Asian American, Native Hawaiian and Pacific Islander tobacco use patterns.. American journal of health behavior; 38.362. | Does not address a KQ |
| 390 | Ping-Ching Hsu, Renny S Lan, Theodore M Brasky, Catalin Marian, Amrita K Cheema, Habtom W Ressom, Christopher A Loffredo, Wallace B Pickworth, Peter G Shields 2017 Menthol Smokers: Metabolomic Profiling and Smoking Behavior.. Cancer epidemiology, biomarkers & prevention : a publication of the American Association for Cancer Research, cosponsored by the American Society of Preventive Oncology; 26.51. | Does not address a KQ |
| 391 | Ping-Ching Hsu, Renny S Lan, Theodore M Brasky, Catalin Marian, Amrita K Cheema, Habtom W Ressom, Christopher A Loffredo, Wallace B Pickworth, Peter G Shields 2017 Metabolomic profiles of current cigarette smokers.. Molecular carcinogenesis; 56.594. | Does not address a KQ |
| 393 | Stacey J Anderson 2011 Menthol cigarettes and smoking cessation behaviour: a review of tobacco industry documents.. Tobacco control; 20 Suppl 2.ii49. | Not original research / data |
| 395 | Valerie B Yerger, Phyra M McCandless 2011 Menthol sensory qualities and smoking topography: a review of tobacco industry documents.. Tobacco control; 20 Suppl 2.ii37. | Not original research / data |
| 396 | Valerie B Yerger 2011 Menthol's potential effects on nicotine dependence: a tobacco industry perspective.. Tobacco control; 20 Suppl 2.ii29. | Not original research / data |
| 401 | Megan E Roberts, Nathan J Doogan, Allison N Kurti, Ryan Redner, Diann E Gaalema, Cassandra A Stanton, Thomas J White, Stephen T Higgins 2016 Rural tobacco use across the United States: How rural and urban areas differ, broken down by census regions and divisions.. Health & place; 39.153. | Does not address a KQ |
| 402 | Olivia A Wackowski, Cristine D Delnevo 2016 Young Adults' Risk Perceptions of Various Tobacco Products Relative to Cigarettes: Results From the National Young Adult Health Survey.. Health education & behavior : the official publication of the Society for Public Health Education; 43.328. | Does not address a KQ |
| 403 | Patricia Folan, Raisa Abramova, Christine Fardellone 2017 What Is Menthol?.. American journal of respiratory and critical care medicine; 196.P9. | Does not address a KQ |
| 404 | Joelle M Lester, Stacey Younger Gagosian 2017 Finished with Menthol: An Evidence-Based Policy Option That Will Save Lives.. The Journal of law, medicine & ethics : a journal of the American Society of Law, Medicine & Ethics; 45.41. | Does not address a KQ |
| 407 | Nathan Gale, Mike McEwan, Alison C Eldridge, Neil Sherwood, Edward Bowen, Simon McDermott, Emma Holmes, Andrew Hedge, Stuart Hossack, Oscar M Camacho, Graham Errington, John McAughey, James Murphy, Chuan Liu, Christopher J Proctor, Ian M Fearon 2017 A randomised, controlled, two-Centre open-label study in healthy Japanese subjects to evaluate the effect on biomarkers of exposure of switching from a conventional cigarette to a tobacco heating product.. BMC public health; 17.673. | Does not address a KQ |
| 409 | Andrea C Villanti, Paul D Mowery, Cristine D Delnevo, Raymond S Niaura, David B Abrams, Gary A Giovino 2016 Changes in the prevalence and correlates of menthol cigarette use in the USA, 2004-2014.. Tobacco control; 25.ii14. | Does not address a KQ |
| 411 | Danielle M Smith, Maansi Bansal-Travers, Jidong Huang, Dianne Barker, Andrew J Hyland, Frank Chaloupka 2016 Association between use of flavoured tobacco products and quit behaviours: findings from a cross-sectional survey of US adult tobacco users.. Tobacco control; 25.ii73. | Does not address a KQ |
| 414 | Jonathan M Samet, Mary Ann Pentz, Jennifer B Unger 2016 Flavoured tobacco products and the public's health: lessons from the TPSAC menthol report.. Tobacco control; 25.ii103. | Does not address a KQ |
| 418 | Man Ki Ho, Babalola Faseru, Won S Choi, Nicole L Nollen, Matthew S Mayo, Janet L Thomas, Kolawole S Okuyemi, Jasjit S Ahluwalia, Neal L Benowitz, Rachel F Tyndale 2009 Utility and relationships of biomarkers of smoking in African-American light smokers.. Cancer epidemiology, biomarkers & prevention : a publication of the American Association for Cancer Research, cosponsored by the American Society of Preventive Oncology; 18.3426. | Duplicate article |
| 419 | Jill C Mwenifumbo, Edward M Sellers, Rachel F Tyndale 2008 Socioeconomic and drug use determinants of smoking status in an urban adult population of Black African descent.. Nicotine & tobacco research : official journal of the Society for Research on Nicotine and Tobacco; 10.1319. | Ineligible population (not U.S., not humans) |
| 421 | Wenlin Huang, Benjamin C Blount, Clifford H Watson, Christina Watson, David M Chambers 2017 Quantitative analysis of menthol in human urine using solid phase microextraction and stable isotope dilution gas chromatography-mass spectrometry.. Journal of chromatography. B, Analytical technologies in the biomedical and life sciences; 1044-1045.200. | Does not address a KQ |
| 425 | Amanda Fallin, Amie J Goodin, Brian A King 2015 Menthol cigarette smoking among lesbian, gay, bisexual, and transgender adults.. American journal of preventive medicine; 48.93. | Does not address a KQ |
| 428 | Cheyenne E Allenby, Kelly A Boylan, Caryn Lerman, Mary Falcone 2016 Precision Medicine for Tobacco Dependence: Development and Validation of the Nicotine Metabolite Ratio.. Journal of neuroimmune pharmacology : the official journal of the Society on NeuroImmune Pharmacology; 11.471. | Does not address a KQ |
| 429 | Naoto Ogura, Yuta Kono, Masako To, Shintaro Mikami, Seiko Soeda, Hiroko Hara, Yasuo To 2017 Menthol-Flavored Cigarettes: Potentially a Strong Trigger of Acute Eosinophilic Pneumonia.. The American journal of medicine; 130.e63. | Does not address a KQ |
| 430 | Jessica Chopyk, Suhana Chattopadhyay, Prachi Kulkarni, Emma Claye, Kelsey R Babik, Molly C Reid, Eoghan M Smyth, Lauren E Hittle, Joseph N Paulson, Raul Cruz-Cano, Mihai Pop, Stephanie S Buehler, Pamela I Clark, Amy R Sapkota, Emmanuel F Mongodin 2017 Mentholation affects the cigarette microbiota by selecting for bacteria resistant to harsh environmental conditions and selecting against potential bacterial pathogens.. Microbiome; 5.22. | Does not address a KQ |
| 432 | Shelley D Golden, Amanda Y Kong, Kurt M Ribisl 2016 Racial and Ethnic Differences in What Smokers Report Paying for Their Cigarettes.. Nicotine & tobacco research : official journal of the Society for Research on Nicotine and Tobacco; 18.1649. | Does not address a KQ |
| 436 | Meike Paschke, Anna Tkachenko, Katja Ackermann, Christoph Hutzler, Frank Henkler, Andreas Luch 2017 Activation of the cold-receptor TRPM8 by low levels of menthol in tobacco products.. Toxicology letters; 271.50. | Does not address a KQ |
| 437 | Cynthia Van Landingham, William Fuller, Greg Mariano, Kristin Marano, Geoffrey Curtin, Sandra I Sulsky 2017 Stroke risk among menthol versus non-menthol cigarette smokers in the United States: Analysis of the National Health and Nutrition Examination Survey (NHANES).. Regulatory toxicology and pharmacology : RTP; 85.64. | Does not address a KQ |
| 438 | Lauren K Lempert, Valerie Yerger, Stanton A Glantz 2016 Letter by Lempert et al Regarding Article, "Menthol and Nonmenthol Cigarette Smoking: All-Cause Deaths, Cardiovascular Disease Deaths, and Other Causes of Death Among Blacks and Whites".. Circulation; 134.e119. | Does not address a KQ |
| 441 | Heather M Munro, Robert E Tarone, Thomas J Wang, William J Blot 2016 Menthol and Nonmenthol Cigarette Smoking: All-Cause Deaths, Cardiovascular Disease Deaths, and Other Causes of Death Among Blacks and Whites.. Circulation; 133.1861. | Does not address a KQ |
| 442 | Linda A Alexander, Dennis R Trinidad, Kari-Lyn K Sakuma, Pallav Pokhrel, Thaddeus A Herzog, Mark S Clanton, Eric T Moolchan, Pebbles Fagan 2016 Why We Must Continue to Investigate Menthol's Role in the African American Smoking Paradox.. Nicotine & tobacco research : official journal of the Society for Research on Nicotine and Tobacco; 18 Suppl 1.S91. | Does not address a KQ |
| 443 | Gary A Giovino, Phillip S Gardiner 2016 Understanding Tobacco Use Behaviors Among African Americans: Progress, Critical Gaps, and Opportunities.. Nicotine & tobacco research : official journal of the Society for Research on Nicotine and Tobacco; 18 Suppl 1.S1. | Does not address a KQ |
| 445 | Wen Qi Gan, Steve Estus, Jonathan H Smith 2016 Association Between Overall and Mentholated Cigarette Smoking With Headache in a Nationally Representative Sample.. Headache; 56.511. | Does not address a KQ |
| 446 | Se-Jung Park, Bitna Yi, Ho-Sun Lee, Woo-Yeon Oh, Hyun-Kyung Na, Minjeong Lee, Mihi Yang 2016 To quit or not: Vulnerability of women to smoking tobacco.. Journal of environmental science and health. Part C, Environmental carcinogenesis & ecotoxicology reviews; 34.33. | Does not address a KQ |
| 448 | Michael S Amato, Joanne D'Silva, Raymond G Boyle 2016 Slowing Menthol's Progress: Differential Impact of a Tobacco Tax Increase on Cigarette Sales.. Nicotine & tobacco research : official journal of the Society for Research on Nicotine and Tobacco; 18.1303. | Does not address a KQ |
| 453 | Daniel Stevens, Stanton Glantz 2016 Tobacco documents reveal questionable professional recertification by industry menthol expert.. Tobacco control; 25.364. | Does not address a KQ |
| 457 | Catherine G Corey, Bridget K Ambrose, Benjamin J Apelberg, Brian A King 2015 Flavored Tobacco Product Use Among Middle and High School Students--United States, 2014.. MMWR. Morbidity and mortality weekly report; 64.1066. | Does not address a KQ |
| 469 | Cheryl A Oncken, Mark D Litt, Lynn D McLaughlin, Nausherwan A Burki 2015 Nicotine concentrations with electronic cigarette use: effects of sex and flavor.. Nicotine & tobacco research : official journal of the Society for Research on Nicotine and Tobacco; 17.473. | Does not address a KQ |
| 475 | Alain Braillon 2015 Curbing the tobacco epidemic: Employing behavioral strategies or rearranging the deckchairs on the Titanic?.. Preventive medicine; 73.28. | Does not address a KQ |
| 479 | Bi-Yu Liu, Yu-Jung Lin, Hung-Fu Lee, Ching-Yin Ho, Ting Ruan, Yu Ru Kou 2015 Menthol suppresses laryngeal C-fiber hypersensitivity to cigarette smoke in a rat model of gastroesophageal reflux disease: the role of TRPM8.. Journal of applied physiology (Bethesda, Md. : 1985); 118.635. | Ineligible population (not U.S., not humans) |
| 488 | Emily Brennan, Laura Gibson, Ani Momjian, Robert C Hornik 2015 Are young people's beliefs about menthol cigarettes associated with smoking-related intentions and behaviors?.. Nicotine & tobacco research : official journal of the Society for Research on Nicotine and Tobacco; 17.81. | Does not address a KQ |
| 491 | Jack E Henningfield 2014 The tobacco endgame: it's all about behavior.. Preventive medicine; 68.11. | Not original research / data |
| 494 | Alain Braillon 2014 Reducing nicotine content of cigarettes: in search of a regulator.. Preventive medicine; 69.306. | Does not address a KQ |
| 495 | M H Zimmermann, D L Richardson, M T B Manderski, C D Delnevo, M B Steinberg 2014 Relighting behaviour among cigarette smokers seeking treatment: implications for tobacco treatment and policy.. International journal of clinical practice; 68.1358. | Does not address a KQ |
| 497 | Shannon M Farley, Hannah Seoh, Rachel Sacks, Michael Johns 2014 Teen use of flavored tobacco products in new york city.. Nicotine & tobacco research : official journal of the Society for Research on Nicotine and Tobacco; 16.1518. | Does not address a KQ |
| 505 | Andrew Cheyne, Lori Dorfman, Richard A Daynard, Pamela Mejia, Mark Gottlieb 2014 The debate on regulating menthol cigarettes: closing a dangerous loophole vs freedom of choice.. American journal of public health; 104.e54. | Does not address a KQ |
| 509 | Brian Rostron 2014 Menthol cigarette use and stroke risk among US smokers: a critical reappraisal.. JAMA internal medicine; 174.808. | Does not address a KQ |
| 510 | Maria Jose Miguez-Burbano, Mayra Vargas, Clery Quiros, John E Lewis, Luis Espinoza, Asthana Deshratan 2014 Menthol cigarettes and the cardiovascular risks of people living with HIV.. The Journal of the Association of Nurses in AIDS Care : JANAC; 25.427. | Does not address a KQ |
| 511 | Olivia A Wackowski, Michelle T Bover Manderski, Cristine D Delnevo 2014 Young adults' behavioral intentions surrounding a potential menthol cigarette ban.. Nicotine & tobacco research : official journal of the Society for Research on Nicotine and Tobacco; 16.876. | Does not address a KQ |
| 514 | Monica Webb Hooper, Elizabeth A Baker, Marcia D McNutt 2014 Racial/Ethnic differences among smokers: revisited and expanded to help seekers.. Nicotine & tobacco research : official journal of the Society for Research on Nicotine and Tobacco; 16.621. | Does not address a KQ |
| 517 | Cendrine D Robinson, Wallace B Pickworth, Stephen J Heishman, Andrew J Waters 2014 The acute tobacco withdrawal syndrome among black smokers.. Psychology of addictive behaviors : journal of the Society of Psychologists in Addictive Behaviors; 28.173. | Does not address a KQ |
| 525 | Kymberle Sterling, Carla J Berg, Akilah N Thomas, Stanton A Glantz, Jasjit S Ahluwalia 2013 Factors associated with small cigar use among college students.. American journal of health behavior; 37.325. | Does not address a KQ |
| 526 | Mike Mitka 2013 FDA might consider restrictions on menthol cigarettes.. JAMA; 310.784. | Not original research / data |
| 531 | Andrea C Villanti, Amanda Richardson, Donna M Vallone, Jessica M Rath 2013 Flavored tobacco product use among U.S. young adults.. American journal of preventive medicine; 44.388. | Does not address a KQ |
| 532 | Juliet P Lee, Sharon Lipperman-Kreda, Sang Saephan, Sean Kirkpatrick 2013 Tobacco environment for Southeast Asian American youth: results from a participatory research project.. Journal of ethnicity in substance abuse; 12.30. | Does not address a KQ |
| 533 | Amanda L Dauphinee, Juliana R Doxey, Nina C Schleicher, Stephen P Fortmann, Lisa Henriksen 2013 Racial differences in cigarette brand recognition and impact on youth smoking.. BMC public health; 13.170. | Does not address a KQ |
| 534 | Andrew A Strasser, Rebecca L Ashare, Madeline Kaufman, Kathy Z Tang, A Clementina Mesaros, Ian A Blair 2013 The effect of menthol on cigarette smoking behaviors, biomarkers and subjective responses.. Cancer epidemiology, biomarkers & prevention : a publication of the American Association for Cancer Research, cosponsored by the American Society of Preventive Oncology; 22.382. | Ineligible comparison (menthol vs non) |
| 535 | Alicia K Matthews, Megan Conrad, Lisa Kuhns, Maria Vargas, Andrea C King 2013 Project Exhale: preliminary evaluation of a tailored smoking cessation treatment for HIV-positive African American smokers.. AIDS patient care and STDs; 27.22. | Does not address a KQ |
| 543 | V Blair Journigan, Nurulain T Zaveri 2013 TRPM8 ion channel ligands for new therapeutic applications and as probes to study menthol pharmacology.. Life sciences; 92.425. | Does not address a KQ |
| 551 | Louise Marsh, Rob McGee, Andrew Gray 2012 A refreshing poison: one-quarter of young New Zealand smokers choose menthol.. Australian and New Zealand journal of public health; 36.495. | Ineligible population (not U.S., not humans) |
| 554 | Peter N Lee, Barbara A Forey, Katharine J Coombs 2012 Systematic review with meta-analysis of the epidemiological evidence in the 1900s relating smoking to lung cancer.. BMC cancer; 12.385. | Does not address a KQ |
| 557 | Alex Cabrera-Serrano, Miriam V Ramos-Colon, Abraham Rivera-Alvarado, Antonio Cases-Rosario, Jessica Irizarry Ramos 2012 Descriptive profile of people with diabetes who use the Puerto Rico Quitline.. Ethnicity & disease; 22.45. | Does not address a KQ |
| 561 | Nicholas T Vozoris 2012 Mentholated cigarettes and cardiovascular and pulmonary diseases: a population-based study.. Archives of internal medicine; 172.590. | Does not address a KQ |
| 562 | Joshua E Muscat, Hsiao-Pin Liu, Steven D Stellman, John P Jr Richie 2012 Menthol smoking in relation to time to first cigarette and cotinine: results from a community-based study.. Regulatory toxicology and pharmacology : RTP; 63.166. | Duplicate article |
| 563 | Valerie M Kramlinger, Linda B von Weymarn, Sharon E Murphy 2012 Inhibition and inactivation of cytochrome P450 2A6 and cytochrome P450 2A13 by menthofuran, beta-nicotyrine and menthol.. Chemico-biological interactions; 197.87. | Does not address a KQ |
| 564 | Marielle C Brinkman, Jane C Chuang, Sydney M Gordon, Hyoshin Kim, Robyn R Kroeger, Gregory M Polzin, Patricia A Richter 2012 Exposure to and deposition of fine and ultrafine particles in smokers of menthol and nonmenthol cigarettes.. Inhalation toxicology; 24.255. | Does not address a KQ |
| 570 | Kolawole S Okuyemi, Babalola Faseru, Gregory A Reed, Lisa Sanderson Cox, Carrie A Bronars, Isaac Opole, Guy-Lucien Whembolua, Matthew S Mayo, Jasjit S Ahluwalia, Neal L Benowitz 2012 Effects of menthol on the pharmacokinetics of bupropion among Black smokers.. Nicotine & tobacco research : official journal of the Society for Research on Nicotine and Tobacco; 14.688. | Does not address a KQ |
| 572 | Lisa Sanderson Cox, Nicole L Nollen, Matthew S Mayo, Won S Choi, Babalola Faseru, Neal L Benowitz, Rachel F Tyndale, Kolawole S Okuyemi, Jasjit S Ahluwalia 2012 Bupropion for smoking cessation in African American light smokers: a randomized controlled trial.. Journal of the National Cancer Institute; 104.290. | Ineligible comparison (menthol vs non) |
| 574 | Joseph R DiFranza 2012 Research opportunities concerning youth and the Family Smoking Prevention and Tobacco Control Act.. Nicotine & tobacco research : official journal of the Society for Research on Nicotine and Tobacco; 14.54. | Not original research / data |
| 584 | Pamela I Clark, Phillip Gardiner 2011 Menthol should not be given a free pass based on studies of biomarkers of toxicity.. Cancer epidemiology, biomarkers & prevention : a publication of the American Association for Cancer Research, cosponsored by the American Society of Preventive Oncology; 20.1269. | Not original research / data |
| 586 | George R Uhl, Donna Walther, Frederique M Behm, Jed E Rose 2011 Menthol preference among smokers: association with TRPA1 variants.. Nicotine & tobacco research : official journal of the Society for Research on Nicotine and Tobacco; 13.1311. | Does not address a KQ |
| 587 | Paul M Wise, George Preti, Jason Eades, Charles J Wysocki 2011 The effect of menthol vapor on nasal sensitivity to chemical irritation.. Nicotine & tobacco research : official journal of the Society for Research on Nicotine and Tobacco; 13.989. | Does not address a KQ |
| 590 | David T Levy, Jennifer L Pearson, Andrea C Villanti, Kenneth Blackman, Donna M Vallone, Raymond S Niaura, David B Abrams 2011 Modeling the future effects of a menthol ban on smoking prevalence and smoking-attributable deaths in the United States.. American journal of public health; 101.1236. | Does not address a KQ |
| 593 | Youn Ok Lee, Stanton A Glantz 2011 Menthol: putting the pieces together.. Tobacco control; 20 Suppl 2.ii1. | Does not address a KQ |
| 594 | Peter N Lee 2011 Systematic review of the epidemiological evidence comparing lung cancer risk in smokers of mentholated and unmentholated cigarettes.. BMC pulmonary medicine; 11.18. | Does not address a KQ |
| 595 | Merrill Goozner 2011 FDA finding may curb mentholated cigarette availability.. Journal of the National Cancer Institute; 103.620. | Not original research / data |
| 597 | Cai Chen, Wentai Luo, Lorne M Isabelle, Keith D Gareau, James F Pankow 2011 The stereoisomers of menthol in selected tobacco products. A brief report.. Nicotine & tobacco research : official journal of the Society for Research on Nicotine and Tobacco; 13.741. | Does not address a KQ |
| 599 | Ralph S Caraballo, David B Holiday, Steven D Stellman, Paul D Mowery, Gary A Giovino, Joshua E Muscat, Michael P Eriksen, John T Bernert, Patricia A Richter, Lynn T Kozlowski 2011 Comparison of serum cotinine concentration within and across smokers of menthol and nonmenthol cigarette brands among non-Hispanic black and non-Hispanic white U.S. adult smokers, 2001-2006.. Cancer epidemiology, biomarkers & prevention : a publication of the American Association for Cancer Research, cosponsored by the American Society of Preventive Oncology; 20.1329. | Does not address a KQ |
| 600 | Clare Dyer 2011 Tobacco firms move to stop FDA banning menthol cigarettes.. BMJ (Clinical research ed.); 342.d1368. | Does not address a KQ |
| 602 | Mansoo Yu 2011 Tobacco use among American Indian or Alaska Native middle- and high-school students in the United States.. Nicotine & tobacco research : official journal of the Society for Research on Nicotine and Tobacco; 13.173. | Does not address a KQ |
| 608 | Cheryl G Healton, Amber Thornton Bullock, William S Robinson, Stacy E Beck, Julia Cartwright, Sharon Y Eubanks 2010 Why we should make menthol cigarettes history.. Nicotine & tobacco research : official journal of the Society for Research on Nicotine and Tobacco; 12 Suppl 2.S94. | Does not address a KQ |
| 609 | Phillip Gardiner, Pamela I Clark 2010 Menthol cigarettes: moving toward a broader definition of harm.. Nicotine & tobacco research : official journal of the Society for Research on Nicotine and Tobacco; 12 Suppl 2.S85. | Not original research / data |
| 612 | Shane P Davis, Annette K McClave-Regan, Valerie J Rock, Judy Kruger, Bridgette E Garrett 2010 Perceptions of menthol cigarette use among U.S. adults and adult smokers: findings from the 2009 HealthStyles survey.. Nicotine & tobacco research : official journal of the Society for Research on Nicotine and Tobacco; 12 Suppl 2.S125. | Does not address a KQ |
| 613 | Valerie J Rock, Shane P Davis, Stacy L Thorne, Kat J Asman, Ralph S Caraballo 2010 Menthol cigarette use among racial and ethnic groups in the United States, 2004-2008.. Nicotine & tobacco research : official journal of the Society for Research on Nicotine and Tobacco; 12 Suppl 2.S117. | Does not address a KQ |
| 614 | Karen Ahijevych, Bridgette E Garrett 2010 The role of menthol in cigarettes as a reinforcer of smoking behavior.. Nicotine & tobacco research : official journal of the Society for Research on Nicotine and Tobacco; 12 Suppl 2.S110. | Not original research / data |
| 615 | Jonathan Foulds, Monica Webb Hooper, Mark J Pletcher, Kolawole S Okuyemi 2010 Do smokers of menthol cigarettes find it harder to quit smoking?.. Nicotine & tobacco research : official journal of the Society for Research on Nicotine and Tobacco; 12 Suppl 2.S102. | Ineligible comparison (menthol vs non) |
| 619 | Anita Fernander, Mary Kay Rayens, Ellen Hahn, Mei Zhang, Sarah M Adkins 2010 Menthol smoking, smoke-free policies and cessation services.. Addiction (Abingdon, England); 105 Suppl 1.105. | Does not address a KQ |
| 630 | Karyn Hede 2010 Drilling down to the causes of racial disparities in lung cancer.. Journal of the National Cancer Institute; 102.1385. | Does not address a KQ |
| 635 | J Daniel Heck 2010 A review and assessment of menthol employed as a cigarette flavoring ingredient.. Food and chemical toxicology : an international journal published for the British Industrial Biological Research Association; 48 Suppl 2.S1. | Does not address a KQ |
| 641 | Lisa B Signorello, Qiuyin Cai, Robert E Tarone, Joseph K McLaughlin, William J Blot 2009 Racial differences in serum cotinine levels of smokers.. Disease markers; 27.187. | Duplicate article |
| 646 | Raphaela Finkenauer, Cynthia S Pomerleau, Sandy M Snedecor, Ovide F Pomerleau 2009 Race differences in factors relating to smoking initiation.. Addictive behaviors; 34.1056. | Ineligible comparison (menthol vs non) |
| 656 | Anita Fernander, Mitzi Schumacher, Xiaochen Wei, Peter Crooks, Peter Wedlund 2008 Smoking risk and the likelihood of quitting among African-American female light and heavy smokers.. Journal of the National Medical Association; 100.1199. | Ineligible comparison (menthol vs non) |
| 657 | Wen Qi Gan, Sigal Ben-Zaken Cohen, S F Paul Man, Don D Sin 2008 Sex-related differences in serum cotinine concentrations in daily cigarette smokers.. Nicotine & tobacco research : official journal of the Society for Research on Nicotine and Tobacco; 10.1293. | Does not address a KQ |
| 658 | Jennifer M Kreslake, Geoffrey Ferris Wayne, Hillel R Alpert, Howard K Koh, Gregory N Connolly 2008 Tobacco industry control of menthol in cigarettes and targeting of adolescents and young adults.. American journal of public health; 98.1685. | Does not address a KQ |
| 659 | Gisela I Robles, Devada Singh-Franco, Hoytin Lee Ghin 2008 A review of the efficacy of smoking-cessation pharmacotherapies in nonwhite populations.. Clinical therapeutics; 30.800. | Not original research / data |
| 661 | Patricia Richter, Diane Beistle, Linda Pederson, Michelle O'Hegarty 2008 Small-group discussions on menthol cigarettes: listening to adult African American smokers in Atlanta, Georgia.. Ethnicity & health; 13.171. | Does not address a KQ |
| 662 | Jennifer M Kreslake, Geoffrey Ferris Wayne, Gregory N Connolly 2008 The menthol smoker: tobacco industry research on consumer sensory perception of menthol cigarettes and its role in smoking behavior.. Nicotine & tobacco research : official journal of the Society for Research on Nicotine and Tobacco; 10.705. | Does not address a KQ |
| 667 | Amie L Haas, James L Sorensen, Sharon M Hall, Christine Lin, Kevin Delucchi, Karl Sporer, TeChieh Chen 2008 Cigarette smoking in opioid-using patients presenting for hospital-based medical services.. The American journal on addictions; 17.65. | Does not address a KQ |
| 673 | Gregory N Connolly, Hillel R Alpert, Geoffrey Ferris Wayne, Howard Koh 2007 Trends in nicotine yield in smoke and its relationship with design characteristics among popular US cigarette brands, 1997-2005.. Tobacco control; 16.e5. | Does not address a KQ |
| 682 | Michael S Werley, Christopher R E Coggins, Peter N Lee 2007 Possible effects on smokers of cigarette mentholation: a review of the evidence relating to key research questions.. Regulatory toxicology and pharmacology : RTP; 47.189. | Not original research / data |
| 684 | Richard Hebert 2006 What's new in Nicotine & Tobacco Research?.. Nicotine & tobacco research : official journal of the Society for Research on Nicotine and Tobacco; 8.481. | Not original research / data |
| 688 | Eric T Moolchan, Frederick H Franken, Maria Jaszyna-Gasior 2006 Adolescent nicotine metabolism: ethnoracial differences among dependent smokers.. Ethnicity & disease; 16.239. | Does not address a KQ |
| 693 | Carolyn C Celebucki, Geoffrey Ferris Wayne, Gregory N Connolly, James F Pankow, Elsa I Chang 2005 Characterization of measured menthol in 48 U.S. cigarette sub-brands.. Nicotine & tobacco research : official journal of the Society for Research on Nicotine and Tobacco; 7.523. | Does not address a KQ |
| 695 | Anita F Fernander, Christi A Patten, Darrell R Schroeder, Susanna R Stevens, Kay M Eberman, Richard D Hurt 2005 Exploring the association of John Henry active coping and education on smoking behavior and nicotine dependence among Blacks in the USA.. Social science & medicine (1982); 60.491. | Does not address a KQ |
| 699 | Eric T Moolchan, Darrell L Hudson, Jennifer R Schroeder, Shelley S Sehnert 2004 Heart rate and blood pressure responses to tobacco smoking among African-American adolescents.. Journal of the National Medical Association; 96.767. | Does not address a KQ |
| 700 | Jed E Rose, Frederique M Behm 2004 Extinguishing the rewarding value of smoke cues: pharmacological and behavioral treatments.. Nicotine & tobacco research : official journal of the Society for Research on Nicotine and Tobacco; 6.523. | Does not address a KQ |
| 701 | Samuel Garten, R Victor Falkner 2004 Role of mentholated cigarettes in increased nicotine dependence and greater risk of tobacco-attributable disease.. Preventive medicine; 38.793. | Does not address a KQ |
| 704 | Eric T Moolchan 2004 Adolescent menthol smokers: will they be a harder target for cessation?.. Nicotine & tobacco research : official journal of the Society for Research on Nicotine and Tobacco; 6 Suppl 1.S93. | Does not address a KQ |
| 708 | Geoffrey Ferris Wayne, Gregory N Connolly 2004 Application, function, and effects of menthol in cigarettes: a survey of tobacco industry documents.. Nicotine & tobacco research : official journal of the Society for Research on Nicotine and Tobacco; 6 Suppl 1.S43. | Does not address a KQ |
| 710 | Karen Ahijevych, Bridgette E Garrett 2004 Menthol pharmacology and its potential impact on cigarette smoking behavior.. Nicotine & tobacco research : official journal of the Society for Research on Nicotine and Tobacco; 6 Suppl 1.S17. | Not original research / data |
| 717 | James M MacDougall, Keith Fandrick, Xiaodong Zhang, Scott V Serafin, John R Cashman 2003 Inhibition of human liver microsomal (S)-nicotine oxidation by (-)-menthol and analogues.. Chemical research in toxicology; 16.988. | Ineligible population (not U.S., not humans) |
| 722 | Kathryn I Pollak, Babafemi Taiwo, Pauline Lyna, Mary Baldwin, Isaac M Lipkus, Gerold Bepler, Colleen M McBride 2002 Reported cessation advice given to African Americans by health care providers in a community health clinic.. Journal of community health; 27.381. | Does not address a KQ |
| 727 | Wallace B Pickworth, Eric T Moolchan, Ivan Berlin, Ram Murty 2002 Sensory and physiologic effects of menthol and non-menthol cigarettes with differing nicotine delivery.. Pharmacology, biochemistry, and behavior; 71.55. | Does not address a KQ |
| 728 | J Appleyard, P Messeri, M L Haviland 2001 Smoking among Asian American and Hawaiian/Pacific Islander youth: data from the 2000 National Youth Tobacco Survey.. Asian American and Pacific Islander journal of health; 9.5. | Does not address a KQ |
| 732 | Anonymous 1999 Women who smoke menthol cigarettes have greater nicotine exposure.. Oncology (Williston Park, N.Y.); 13.915. | Not original research / data |
| 733 | W S Pritchard, M E Houlihan, T D Guy, J H Robinson 1999 Little evidence that "denicotinized" menthol cigarettes have pharmacological effects: an EEG/heart-rate/sujective-response study.. Psychopharmacology; 143.273. | Does not address a KQ |
| 738 | G D Friedman, M Sadler, I S Tekawa, S Sidney 1998 Mentholated cigarettes and non-lung smoking related cancers in California, USA.. Journal of epidemiology and community health; 52.202. | Does not address a KQ |
| 739 | J P Jr Richie, S G Carmella, J E Muscat, D G Scott, S A Akerkar, S S Hecht 1997 Differences in the urinary metabolites of the tobacco-specific lung carcinogen 4-(methylnitrosamino)-1-(3-pyridyl)-1-butanone in black and white smokers.. Cancer epidemiology, biomarkers & prevention : a publication of the American Association for Cancer Research, cosponsored by the American Society of Preventive Oncology; 6.783. | Does not address a KQ |
| 741 | T L Richardson 1997 African-American smokers and cancers of the lung and of the upper respiratory and digestive tracts. Is menthol part of the puzzle?.. The Western journal of medicine; 166.189. | Does not address a KQ |
| 742 | T Richardson 1996 Menthol cigarette use in African Americans.. Hospital practice (1995); 31.22H. | Not original research / data |
| 743 | P I Clark, S Gautam, L W Gerson 1996 Effect of menthol cigarettes on biochemical markers of smoke exposure among black and white smokers.. Chest; 110.1194. | Does not address a KQ |
| 744 | G C Kabat 1996 Aspects of the epidemiology of lung cancer in smokers and nonsmokers in the United States.. Lung cancer (Amsterdam, Netherlands); 15.1. | Does not address a KQ |
| 746 | N Hymowitz, D Corle, J Royce, T Hartwell, K Corbett, M Orlandi, N Piland 1995 Smokers' baseline characteristics in the COMMIT trial.. Preventive medicine; 24.503. | Duplicate article |
| 752 | J M Royce, N Hymowitz, K Corbett, T D Hartwell, M A Orlandi 1993 Smoking cessation factors among African Americans and whites. COMMIT Research Group.. American journal of public health; 83.220. | Does not address a KQ |
| 753 | K Ahijevych, M E Wewers 1993 Factors associated with nicotine dependence among African American women cigarette smokers.. Research in nursing & health; 16.283. | Does not address a KQ |
| 755 | G C Kabat, J R Hebert 1991 Use of mentholated cigarettes and lung cancer risk.. Cancer research; 51.6510. | Does not address a KQ |
| 761 | C T Orleans, V J Schoenbach, M A Salmon, V J Strecher, W Kalsbeek, D Quade, E F Brooks, T R Konrad, C Blackmon, C D Watts 1989 A survey of smoking and quitting patterns among black Americans.. American journal of public health; 79.176. | Does not address a KQ |
| 765 | J R Hebert, G C Kabat 1988 Menthol cigarettes and esophageal cancer.. American journal of public health; 78.986. | Does not address a KQ |
| 768 | G Camarasa, A Alomar 1978 Menthol dermatitis from cigarettes.. Contact dermatitis; 4.169. | Does not address a KQ |
| 769 | B B Chrisman 1978 Menthol and dermatitis.. Archives of dermatology; 114.286. | Does not address a KQ |
| 770 | P K Das, R S Rathor, P S Sinha, A K Sanyal 1970 Effect on ciliary movements of some agents which come in contact with the respiratory tract.. Indian journal of physiology and pharmacology; 14.297. | Does not address a KQ |
| 771 | I Schmeltz, W S Schlotzhauer 1968 Benzo(a)pyrene, phenols and other products from pyrolysis of the cigarette additive, (d,1)-menthol.. Nature; 219.370. | Does not address a KQ |
| 772 | E M McGowan 1966 Menthol urticaria.. Archives of dermatology; 94.62. | Does not address a KQ |
| 773 | W SIMON, R J LUCERO 1960 Consumption of mentholated cigarettes by alcoholics.. Diseases of the nervous system; 21.213. | Does not address a KQ |
| 774 | N RAKIETEN, M L RAKIETEN, D FELDMAN, M J Jr BOYKIN 1952 Mammalian ciliated respiratory epithelium; studies with particular reference to effects of menthol, nicotine, and smoke of mentholated and nonmentholated cigarettes.. A.M.A. archives of otolaryngology; 56.494. | Does not address a KQ |
| 775 | B HIGHSTEIN, I ZELIGMAN 1951 Nonthrombocytopenic purpura caused by mentholated cigarettes.. Journal of the American Medical Association; 146.816. | Does not address a KQ |
| 776 | Valeria Lallai, Christie D Fowler 2017 More than just chillin': Interactive effects of menthol and nicotine in drug reward.. Neuropsychopharmacology; 42.2283. | Does not address a KQ |
| 783 | Eniye Emmanuel Eguae 2018 Factors associated with menthol cigarettes smoking among youths ages 12 to 19.. Dissertation Abstracts International: Section B: The Sciences and Engineering; 79.No. | Does not address a KQ |
| 786 | Davide Risso, Eduardo Sainz, Joanne Gutierrez, Thomas Kirchner, Raymond Niaura, Dennis Drayna 2017 Association of TAS2R38 haplotypes and menthol cigarette preference in an African American cohort.. Nicotine & Tobacco Research; 19.493. | Does not address a KQ |
| 789 | Teagan Rose Wall 2016 Effects of TI-299423 on neuronal nicotinic acetylcholine receptors.. Dissertation Abstracts International: Section B: The Sciences and Engineering; 76.No. | Does not address a KQ |
| 791 | Esteban Petruzzello 2016 Studies in the economics of addiction.. Dissertation Abstracts International Section A: Humanities and Social Sciences; 76.No. | Does not address a KQ |
| 794 | R. F Gillum, B West 2015 Religion and menthol cigarette use in a US national sample.. Preventive Medicine: An International Journal Devoted to Practice and Theory; 81.444. | Does not address a KQ |
| 795 | Emily Brennan, Laura Gibson, Ani Momjian, Robert C Hornik 2015 "Are young people's beliefs about menthol cigarettes associated with smoking-related intentions and behaviors?": Erratum.. Nicotine & Tobacco Research; 17.1403. | Does not address a KQ |
| 796 | Olivia A Wackowski, Cristine D Delnevo, Jennifer L Pearson 2015 Switching to E-cigarettes in the event of a menthol cigarette ban.. Nicotine & Tobacco Research; 17.1286. | Does not address a KQ |
| 799 | Cristine D Delnevo, Andrea C Villanti, Gary A Giovino 2014 Trends in menthol and non-menthol cigarette consumption in the USA: 2000-2011.. Tobacco Control: An International Journal; 23.e154. | Does not address a KQ |
| 801 | Man Ki Ho 2014 Impact of CYP2A6 genetic variation on nicotine metabolism and smoking behaviours in light smoking populations of Black-African descent.. Dissertation Abstracts International: Section B: The Sciences and Engineering; 74.No. | Ineligible population (not U.S., not humans) |
| 806 | Matthew I Palmatier, Jaden E Lantz, Laura C O'Brien, Sarah P Metz 2013 Effects of nicotine on olfactogustatory incentives: Preference, palatability, and operant choice tests.. Nicotine & Tobacco Research; 15.1545. | Ineligible population (not U.S., not humans) |
| 808 | Brian Rostron 2013 Methodology, menthol, and mortality.. Nicotine & Tobacco Research; 15.619. | Does not address a KQ |
| 809 | Andrea C Villanti, Gary A Giovino, David M Burns, David B Abrams 2013 Menthol cigarettes and mortality: Keeping focus on the public health standard.. Nicotine & Tobacco Research; 15.617. | Not original research / data |
| 810 | Lawrence Carter 2013 Commentary on nonnemaker et al. (2013): Banning menthol cigarettes-Is it time to conduct the experiment?. Addiction; 108.179. | Not original research / data |
| 812 | Brian Rostron 2012 Response to "Menthol cigarettes and lung cancer mortality".. Nicotine & Tobacco Research; 14.1248. | Does not address a KQ |
| 813 | Joshua E Muscat 2012 Menthol cigarettes and lung cancer mortality.. Nicotine & Tobacco Research; 14.1246. | Does not address a KQ |
| 815 | Valerie J Rock, Shane P Davis, Stacy L Thorne, Ralph S Caraballo 2012 Menthol cigarette use: The challenge to improve measurement and monitoring among adolescent smokers.. Nicotine & Tobacco Research; 14.251. | Not original research / data |
| 817 | Neal L Benowitz, Jonathan M Samet 2011 The threat of menthol cigarettes to U.S. public health.. The New England Journal of Medicine; 364.2179. | Not original research / data |
| 818 | Michael Siegel 2011 A lost opportunity for public health - The FDA Advisory Committee Report on Menthol.. The New England Journal of Medicine; 364.2177. | Not original research / data |
| 819 | Richard F Gillum 2011 Effect of religion on use of menthol cigarettes in African American smokers.. Preventive Medicine: An International Journal Devoted to Practice and Theory; 52.477. | Does not address a KQ |
| 820 | Christian Andreas Arist Von Hehn 2010 TRPA1 as a sensory neuronal target of chemical irritants and counterirritants.. Dissertation Abstracts International: Section B: The Sciences and Engineering; 71.129. | Ineligible population (not U.S., not humans) |
| 822 | Brian P Flaherty 2010 Latent class and mixture models' potential contributions to understanding connections between menthol and other cigarette smoking characteristics.. Addiction; 105.11. | Not original research / data |
| 825 | Kolawole S Okuyemi, Deirdre Lawrence, George Hammons, Linda A Alexander 2010 Use of mentholated cigarettes: What can we learn from national data sets?. Addiction; 105.1. | Not original research / data |
| 832 | Richard Hebert 2004 What's New in Nicotine & Tobacco Research?. Nicotine & Tobacco Research; 6.S1. | Not original research / data |
| 836 | Wallace B Pickworth, Reginald V Fant, Richard A Nelson, Melissa S Rohrer, Jack E Henningfield 1999 Pharmacodynamic effects of new de-nicotinized cigarettes.. Nicotine & Tobacco Research; 1.357. | Does not address a KQ |
| 838 | David P Schmahl, Edward Lichtenstein, Darrel E Harris 1972 Successful treatment of habitual smokers with warm, smoky air and rapid smoking.. Journal of Consulting and Clinical Psychology; 38.105. | Does not address a KQ |
| 851 | Stacy Thorne, Shane Davis, Ralph S. Caraballo 2010 Menthol use and nicotine dependence among US adults, 2001-2016.. American Public Health Association. | Does not address a KQ |
| 869 | Villanti, Andrea C., Johnson, Amanda L., Glasser, Allison M., Rose, Shyanika W., Ambrose, Bridget K., Conway, Kevin P., Cummings, K. Michael, Stanton, Cassandra A., Edwards, Kathryn C., Delnevo, Cristine D., Wackowski, Olivia A., Feirman, Shari P., Bansal-Travers, Maansi, Bernat, Jennifer K., Holder-Hayes, Enver, Green, Victoria R., Silveira, Marushka L., Hyland, Andrew 2019 Association of Flavored Tobacco Use With Tobacco Initiation and Subsequent Use Among US Youth and Adults, 2013-2015. JAMA network open; 2. | Ineligible comparison (menthol vs non) |
| 873 | Gideon St.Helen, Neal L. Benowitz, Jasjit S. Ahluwalia, Rachel F. Tyndale, Newton Addo, Steven E. Gregorich, Eliseo J. Pérez-Stable, Lisa Sanderson Cox 2019 Black Light Smokers: How Nicotine Intake and Carcinogen Exposure Differ Across Various Biobehavioral Factors.. Journal of the National Medical Association; 111.509. | Does not address a KQ |
| 900 | Alain Braillon 2019 Could Tobacco Control Policies Be a Smokescreen?. JAMA Pediatrics; 174.102. | Not original research / data |
| 938 | Krysten W Bold, Peter Jatlow, Lisa M Fucito, Tore Eid, Suchitra Krishnan-Sarin, Stephanie O'Malley 2020 Evaluating the effect of switching to non-menthol cigarettes among current menthol smokers: an empirical study of a potential ban of characterising menthol flavour in cigarettes.. Tobacco Control; 29.624. | Ineligible comparison (menthol vs non) |
| 942 | Keith Wailoo 2019 The FDA's Proposed Ban on Menthol Cigarettes.. The New England Journal of Medicine; 380.995. | Not original research / data |
| 949 | Karen A Cullen, Sherry T Liu, Jennifer K Bernat, Wendy I Slavit, Michael A Tynan, Brian A King, Linda J Neff 2019 Flavored Tobacco Product Use Among Middle and High School Students - United States, 2014-2018.. MMWR and Morbidity and Mortality Weekly Report; 68.839. | Does not address a KQ |
| 956 | Pamela Valera Nicholas Acuna 2019 Group-based smoking cessation treatment for incarcerated men: A pilot study.. Proceedings of the American Association for Cancer Research Annual Meeting 2019; 79.LB-252. | Ineligible comparison (menthol vs non) |
| 961 | Launick Saint-Fort, Kelvin Choi 2019 Heterogeneity in Tobacco-Use Behaviors Among U.S. Blacks per Global Region of Origin.. Journal of Immigrant and Minority Health; 21.1185. | Does not address a KQ |
| 992 | Amy M Cohn, Shyanika W Rose, Joanne D'Silva, Andrea C Villanti 2019 Menthol Smoking Patterns and Smoking Perceptions Among Youth: Findings From the Population Assessment of Tobacco and Health Study.. American Journal of Preventive Medicine; 56.E107. | Ineligible comparison (menthol vs non) |
| 1004 | Cheryl Oncken, Erin L Mead, Ellen A Dornelas, Chia-Ling Kuo, Heather Z Sankey, Henry R Kranzler, Sheila Thurlow 2020 Opioid Use and Rate of Nicotine Metabolism Among Pregnant Smokers.. Nicotine & Tobacco Research; 22.1046 | Ineligible comparison (menthol vs non) |
| 1010 | John H Kingsbury, Komal Mehrotra, Joanne D'Silva, Eugene Nichols, Ruth Tripp, David Johnson 2020 Perceptions of Menthol Cigarettes and Reasons for Unsuccessful Quits in an African American Community Sample.. Journal of Immigrant and Minority Health. | Does not address a KQ |
| 1011 | Allison M Glasser , Alexis Barton, Jessica Rath, Bethany Simard, Shyanika W Rose, Elizabeth Hair, Donna Vallone 2020 Perceptions of Use Patterns and Health Consequences Associated With Mentholated Cigarettes Among U.S. Adults.. Health Education & Behavior; 47.284. | Does not address a KQ |
| 1024 | Andrea H Weinberger, Daniel P Giovenco, Jiaqi Zhu, Joun Lee, Rachel S Kashan, Renee D Goodwin 2019 Racial/ethnic differences in daily, nondaily, and menthol cigarette use and smoking quit ratios in the United States: 2002 to 2016.. Preventive Medicine; 125.32. | Does not address a KQ |
| 1027 | Jessica L Barrington-Trimis 2020 Reducing the Adverse Public Health Impact of Menthol Cigarettes in Disparate Populations.. Nicotine & Tobacco Research; 22.456. | Does not address a KQ |
| 1029 | Barbara Campbell, Deborah Yip, Thao Le, Noah Gubner, Joseph Guydish 2019 Relationship between Tobacco Use and Health-Related Quality of Life (HRQoL) among Clients in Substance Use Disorders Treatment.. Journal of Psychoactive Drugs; 51.48. | Does not address a KQ |
| 1041 | Adriaan W Bruijnzeel 2020 Shifting Frontiers in Basic Research on Nicotine and Tobacco Products.. Nicotine & Tobacco Research; 22.145. | Does not address a KQ |
| 1061 | Kevin R J Schroth, Andrea C Villanti, Marin Kurti, Cristine D Delnevo 2019 Why an FDA Ban on Menthol Is Likely to Survive a Tobacco Industry Lawsuit.. Public Health Reports; 134.300. | Does not address a KQ |
