# Supplementary material for: Assessing the evidence on the differential impact of menthol versus non-menthol cigarette use on smoking cessation in the U.S. population: a systematic review and meta-analysis
Source: Subst Abuse Treat Prev Policy. 2021 Aug 11;16:61. doi: 10.1186/s13011-021-00397-4 (PMC8359586; doi:10.1186/s13011-021-00397-4)
Supplement: Supplementary file 3 — Additional file 3. Outcome Measures for Smoking Cessation across Adjusted Studies. [file 13011_2021_397_MOESM3_ESM.docx]

**SUPPLEMENTAL SECTION 3: Outcome Measures for Smoking Cessation across Adjusted Studies**

**SUPPLEMENTAL SECTION 3: Outcome Measures for Smoking Cessation across Adjusted Studies**

| **Measure and definition** | **Studies** |
| --- | --- |
| **Duration of abstinence** | |
| Quit between 3 months and 1 year; quit between 3 months and 5 years among former smokers | Levy et al., 2011 (1) |
| Time since quit (continuous measure) among former smokers | Cubbin et al., 2010 (2) |
| **Quit attempts (any quit attempts; number of quit attempts per person)** | |
| Tried to quit smoking in the past 12 months | Keeler et al., 2017 (3), Keeler et al., 2018 (4), Park, 2017 (5), Cubbin et al., 2010 (2), Schneller et al., 2020 (6); Schneller, 2020 (7) |
| At least 24 hours without smoking in the past 12 months because [respondent was] trying to quit smoking | Kahende et al., 2011 (8), Levy et al., 2011 (1), Webb Hooper et al., 2011 (9), Fagan et al., 2007 (10) |
| At least 24 hours without smoking *ever* because [respondent was] trying to quit smoking | Alexander et al., 2010 (11), Rath et al., 2015 (12) |
| At least 24 hours without smoking in the past 6 months | Rath et al., 2015 (12) |
| Any attempts (undefined) to quit smoking [since previous interview] | Kasza et al., 2014 (13), Pletcher et al., 2006 (14) |
| Use of a cessation program or aid | Stahre et al., 2010 (15) |
| Other or definition not reported (number of quit attempts) | Hyland & Rivard, 2010 (16) |
| **Rate of abstinence/quitting (including but not limited to prolonged abstinence (PA), point prevalence abstinence (PPA), identifiable cigarette type [menthol versus non-menthol] smoked before quitting, and being a former smoker [versus current smoker])** | |
| Not smoking at all in past 6 months | Hyland & Rivard, 2010 (16), A. Hyland et al., 2002 (17), Trinidad et al., 2010 (18) |
| Not smoking at all in the past 3 months | Keeler et al., 2017 (3), Keeler et al., 2018 (4) |
| Not purchasing a pack in past year | Lewis et al., 2014 (19) |
| Former smoker vs. current smoker | Sulsky et al., 2014 (20), Muscat et al., 2002 (21), Delnevo et al., 2011 (22), Delnevo et al., 2010 (23) |
| Ever-smokers identifying as “non-smoker” or smoking “not at all” or “not currently” at interview/survey | Blot et al., 2011 (24), Gundersen et al., 2009 (25), Pletcher et al., 2006 (14), Schneller et al., 2020 (6); Schneller, 2020 (7) |
| Transition from non-established smoking to non-smoking | Nonnemaker et al., 2012 (26) |
| 7-day PPA | Rojewski et al., 2014 (27), Faseru et al., 2013 (28), Winhusen et al., 2013 (29), Steinberg et al., 2011 (30), Cropsey et al., 2009 (31), Gandhi et al., 2009 (32), Fu et al., 2008 (33), Foulds et al., 2006 (34), Okuyemi et al., 2003 (35), Okuyemi et al., 2007 (36) |
| 30-day PPA | D'Silva et al., 2012 (37) |
| PA since baseline or quit date | Reitzel et al., 2013 (38), Reitzel, 2011b (39), Reitzel, 2011a (40), Reitzel, 2011c;  Reitzel et al., 2011 (41, 42) |
| Rate of abstinence/quitting (abstinence definition not reported) | Thihalolipavan et al., 2014 (43) |
| **Change in smoking quantity/frequency** | |
| Difference/change in CPD | Gubner, et al., 2018 (44), A. Hyland et al., 2002 (17), Hyland & Rivard, 2010 (16), Reitzel, 2011c (41), Azagba et al., 2020 (45), Sawdey et al., 2020 (46) |
| **Return to smoking/relapse** | |
| (Rate of) return to smoking | Pletcher et al., 2006 (14), Muench & Juliano, 2017 (47) |

CPD=cigarettes per day; PA=prolonged abstinence; PPA=point (or period) prevalence abstinence

**References**

1. Alexander LA, Crawford T, Mendiondo MS. Occupational status, work-site cessation programs and policies and menthol smoking on quitting behaviors of US smokers. Addiction. 2010;105 Suppl 1:95-104.

2. Azagba S, King J, Shan L, Manzione L. Cigarette Smoking Behavior Among Menthol and Nonmenthol Adolescent Smokers. J Adolesc Health. 2020;66(5):545-50.

3. Blot WJ, Cohen SS, Aldrich M, McLaughlin JK, Hargreaves MK, Signorello LB. Lung cancer risk among smokers of menthol cigarettes. J Natl Cancer Inst. 2011;103(10):810-6.

4. Cropsey KL, Weaver MF, Eldridge GD, Villalobos GC, Best AM, Stitzer ML. Differential success rates in racial groups: results of a clinical trial of smoking cessation among female prisoners. Nicotine Tob Res. 2009;11(6):690-7.

5. Cubbin C, Soobader MJ, LeClere FB. The intersection of gender and race/ethnicity in smoking behaviors among menthol and non-menthol smokers in the United States. Addiction. 2010;105 Suppl 1:32-8.

6. D'Silva J, Boyle RG, Lien R, Rode P, Okuyemi KS. Cessation outcomes among treatment-seeking menthol and nonmenthol smokers. Am J Prev Med. 2012;43(5 Suppl 3):S242-8.

7. Delnevo CD, Gundersen DA, Hrywna M. Examining the relationship between menthol smoking and cessation using data from the 2003 and 2006/7 Tobacco Use Supplement. Center for Tobacco Surveillance and Evaluation Research: University of Medicine & Dentistry of New Jersey - School of Public Health; 2010 January 10-11, 2011.

8. Delnevo CD, Gundersen DA, Hrywna M, Echeverria SE, Steinberg MB. Smoking-cessation prevalence among U.S. smokers of menthol versus non-menthol cigarettes. Am J Prev Med. 2011;41(4):357-65.

9. Fagan P, Augustson E, Backinger CL, O'Connell ME, Vollinger RE, Jr., Kaufman A, et al. Quit attempts and intention to quit cigarette smoking among young adults in the United States. Am J Public Health. 2007;97(8):1412-20.

10. Faseru B, Nollen NL, Mayo MS, Krebill R, Choi WS, Benowitz NL, et al. Predictors of cessation in African American light smokers enrolled in a bupropion clinical trial. Addict Behav. 2013;38(3):1796-803.

11. Foulds J, Gandhi KK, Steinberg MB, Richardson DL, Williams JM, Burke MV, et al. Factors associated with quitting smoking at a tobacco dependence treatment clinic. Am J Health Behav. 2006;30(4):400-12.

12. Fu SS, Okuyemi KS, Partin MR, Ahluwalia JS, Nelson DB, Clothier BA, et al. Menthol cigarettes and smoking cessation during an aided quit attempt. Nicotine Tob Res. 2008;10(3):457-62.

13. Gandhi KK, Foulds J, Steinberg MB, Lu SE, Williams JM. Lower quit rates among African American and Latino menthol cigarette smokers at a tobacco treatment clinic. Int J Clin Pract. 2009;63(3):360-7.

14. Gubner NR, Williams DD, Pagano A, Campbell BK, Guydish J. Menthol cigarette smoking among individuals in treatment for substance use disorders. Addictive behaviors. 2018;80:135-41.

15. Gundersen DA, Delnevo CD, Wackowski O. Exploring the relationship between race/ethnicity, menthol smoking, and cessation, in a nationally representative sample of adults. Prev Med. 2009;49(6):553-7.

16. Hyland A, Garten S, Giovino GA, Cummings KM. Mentholated cigarettes and smoking cessation: Findings from COMMIT. Tob Control. 2002;11:135-9.

17. Hyland A, Rivard C. Analysis of mentholated cigarettes using the COMMIT data -- summary. Department of Health Behavior, Roswell Park Cancer Institute; 2010.

18. Kahende JW, Malarcher AM, Teplinskaya A, Asman KJ. Quit attempt correlates among smokers by race/ethnicity. International journal of environmental research and public health. 2011;8(10):3871-88.

19. Kasza KA, Hyland AJ, Bansal-Travers M, Vogl LM, Chen J, Evans SE, et al. Switching between menthol and nonmenthol cigarettes: findings from the U.S. Cohort of the International Tobacco Control Four Country Survey. Nicotine Tob Res. 2014;16(9):1255-65.

20. Keeler C, Max W, Yerger V, Yao T, Ong MK, Sung H-Y. The Association of Menthol Cigarette Use With Quit Attempts, Successful Cessation, and Intention to Quit Across Racial/Ethnic Groups in the United States. Nicotine & tobacco research : official journal of the Society for Research on Nicotine and Tobacco. 2017;19(12):1450-64.

21. Keeler C, Max W, Yerger VB, Yao T, Wang Y, Ong MK, et al. Effects of cigarette prices on intention to quit, quit attempts, and successful cessation among African American smokers. Nicotine & tobacco research : official journal of the Society for Research on Nicotine and Tobacco. 2018.

22. Levy DT, Blackman K, Tauras J, Chaloupka FJ, Villanti AC, Niaura RS, et al. Quit attempts and quit rates among menthol and nonmenthol smokers in the United States. Am J Public Health. 2011;101(7):1241-7.

23. Lewis M, Wang Y, Berg CJ. Tobacco control environment in the United States and individual consumer characteristics in relation to continued smoking: differential responses among menthol smokers? Prev Med. 2014;65:47-51.

24. Muench C, Juliano LM. Predictors of smoking lapse during a 48-hour laboratory analogue smoking cessation attempt. Psychology of addictive behaviors : journal of the Society of Psychologists in Addictive Behaviors. 2017;31(4):415-22.

25. Muscat JE, Richie JP, Jr., Stellman SD. Mentholated cigaettes and smoking habits in whites and blacks. Tob Control. 2002;11:368-71.

26. Nonnemaker J, Hersey J, Homsi G, Busey A, Allen J, Vallone D. Initiation with menthol cigarettes and youth smoking uptake. Addiction. 2012;108(1):171-8.

27. Okuyemi KS, Ahluwalia JS, Ebersole-Robinson M, Catley D, Mayo MS, Resnicow K. Does menthol attenuate the effect of bupropion among African American smokers? Addiction. 2003;98:1387-93.

28. Okuyemi KS, Faseru B, Sanderson Cox L, Bronars CA, Ahluwalia JS. Relationship between menthol cigarettes and smoking cessation among African American light smokers. Addiction. 2007;102(12):1979-86.

29. Park J-Y. Tobacco use behaviors among vulnerable populations. Dissertation Abstracts International: Section B: The Sciences and Engineering. 2017;77(12-B(E)):No-Specified.

30. Pletcher MJ, Hulley BJ, Houston T, Kiefe CI, Benowitz N, Sidney S. Menthol cigarettes, smoking cessation, atherosclerosis, and pulmonary function. Arch Intern Med. 2006;166:1915-22.

31. Rath JM, Villanti AC, Williams VF, Richardson A, Pearson JL, Vallone DM. Patterns of Longitudinal Transitions in Menthol Use Among US Young Adult Smokers. Nicotine Tob Res. 2015;17(7):839-46.

32. Reitzel LR. Menthol cigarettes, tobacco dependence, and smoking cessation: Project BREAK FREE final report. 2011.

33. Reitzel LR. Menthol cigarettes, tobacco dependence, and smoking cessation: Project CARE final report. 2011.

34. Reitzel LR. Menthol cigarettes, tobacco dependence, and smoking cessation: Project MOM final report. 2011.

35. Reitzel LR, Nguyen N, Cao Y, Vidrine JI, Daza P, Mullen PD, et al. Race/ethnicity moderates the effect of prepartum menthol cigarette use on postpartum smoking abstinence. Nicotine Tob Res. 2011;13(12):1305-10.

36. Reitzel LR, Li Y, Stewart DW, Cao Y, Wetter DW, Waters AJ, et al. Race moderates the effect of menthol cigarette use on short-term smoking abstinence. Nicotine Tob Res. 2013;15(5):883-9.

37. Rojewski AM, Toll BA, O'Malley SS. Menthol cigarette use predicts treatment outcomes of weight-concerned smokers. Nicotine Tob Res. 2014;16(1):115-9.

38. Sawdey MD, Chang JT, Cullen KA, Rass O, Jackson KJ, Ali FRM, et al. Trends and Associations of Menthol Cigarette Smoking Among US Middle and High School Students-National Youth Tobacco Survey, 2011-2018. Nicotine Tob Res. 2020;22(10):1726-35.

39. Schneller LM. Assessment of various delivery methods of menthol in cigarettes sold in the US. Dissertation Abstracts International: Section B: The Sciences and Engineering. 2020;81.

40. Schneller LM, Bansal-Travers M, Mahoney MC, McCann SE, O'Connor RJ. Menthol Cigarettes and Smoking Cessation among Adult Smokers in the US. Am J Health Behav. 2020;44(2):252-6.

41. Stahre M, Okuyemi KS, Joseph AM, Fu SS. Racial/ethnic differences in menthol cigarette smoking, population quit ratios and utilization of evidence-based tobacco cessation treatments. Addiction. 2010;105 Suppl 1:75-83.

42. Steinberg MB, Bover MT, Richardson DL, Schmelzer AC, Williams JM, Foulds J. Abstinence and psychological distress in co-morbid smokers using various pharmacotherapies. Drug Alcohol Depend. 2011;114(1):77-81.

43. Sulsky SI, Fuller WG, Van Landingham C, Ogden MW, Swauger JE, Curtin GM. Evaluating the association between menthol cigarette use and the likelihood of being a former versus current smoker. Regul Toxicol Pharmacol. 2014;70(1):231-41.

44. Thihalolipavan S, Jung M, Jasek J, Chamany S. Menthol smokers in large-scale nicotine replacement therapy program. Am J Public Health. 2014;104(11):e3-4.

45. Trinidad DR, Perez-Stable EJ, Messer K, White MM, Pierce JP. Menthol cigarettes and smoking cessation among racial/ethnic groups in the United States. Addiction. 2010;105 Suppl 1:84-94.

46. Webb Hooper M, Zhao W, Byrne MM, Davila E, Caban-Martinez A, Dietz NA, et al. Menthol cigarette smoking and health, Florida 2007 BRFSS. Am J Health Behav. 2011;31(1):3-14.

47. Winhusen TM, Adinoff B, Lewis DF, Brigham GS, Gardin JG, 2nd, Sonne SC, et al. A tale of two stimulants: mentholated cigarettes may play a role in cocaine, but not methamphetamine, dependence. Drug Alcohol Depend. 2013;133(3):845-51.
