# Supplementary material for: Assessing the evidence on the differential impact of menthol versus non-menthol cigarette use on smoking cessation in the U.S. population: a systematic review and meta-analysis
Source: Subst Abuse Treat Prev Policy. 2021 Aug 11;16:61. doi: 10.1186/s13011-021-00397-4 (PMC8359586; doi:10.1186/s13011-021-00397-4)
Supplement: Supplementary file 4 — Additional file 4 Evidence Table, Modeled / Adjusted Results (Duration of Abstinence, Any Quit Attempt, Number of Quit Attempts per Person, Rate of Abstinence/Quitting, Change in Smoking Quantity/Frequency, and Return to Smoking/Relapse) (n = 43 studies; n = 47 references). [file 13011_2021_397_MOESM4_ESM.docx]

**SUPPLEMENTAL SECTION 4: Evidence Table, Modeled / Adjusted Results (Duration of Abstinence, Any Quit Attempt, Number of Quit Attempts per Person, Rate of Abstinence/Quitting, Change in Smoking Quantity/Frequency, and Return to Smoking/Relapse) (n=43 studies; n=47 references)**

**SUPPLEMENTAL SECTION 4: Evidence Table, Modeled / Adjusted Results (Duration of Abstinence, Any Quit Attempt, Number of Quit Attempts per Person, Rate of Abstinence/Quitting, Change in Smoking Quantity/Frequency, and Return to Smoking/Relapse) (n=43 studies; n=47 references)**

| **First Author, Year,**  **National survey name,**  **special population;**  **Overall study quality rating;**  **Individual study quality rating domain score (48)** | **Menthol as dependent or independent variable;**  **Model;**  **Covariates** | **Duration of abstinence** | **Any quit attempt** | **Number of quit attempts per person** | **Rate of abstinence/quitting** | **Change in smoking quantity/frequency** | **Return to smoking/relapse** |
| --- | --- | --- | --- | --- | --- | --- | --- |
| Alexander et al., 2010 (11),  TUS-CPS  None ;  Good (20);  Reporting (9); external validity (1); internal validity/bias (5); internal validity confounding (4); power (1) | Independent;  Multiple logistic regression;  Age, gender, race, education, occupation, income, U.S. region, workplace smoking policies and restrictions | NR | Stopped smoking for ≥1 day  OR (95% CI) for M (NM is ref):  0.98 (0.83 to 1.15) | NR | NR | NR | NR |
| Azagba et al., 2019 (45)  NYTS  Middle school (grades 6 to 8) and  high school (grades 9 to 12);  Fair (19): reporting (8); external validity (3); internal validity/bias (4); internal validity confounding (3); power (1) | Dependent;  Logistic regression;  Grade, gender, race/ethnicity, other tobacco use, survey year (time trend) | NR | NR | NR | NR | Smoking ≥10 days in the past 30, OR for M (ref=NM and smoking 1-9 days in the past 30)  *Total*  AOR=1.48, 95% CI, 1.14 to 1.94; p<0.05  *Middle school*  AOR=2.36, 95% CI, 1.01 to 5.49; p<0.05  *High school*  AOR=1.41, 95% CI, 1.09 to 1.82; p<0.05  Smoking ≥20 days in the past 30, OR for M (ref=NM and smoking 1-19 days in the past 30)  *Total*  AOR=1.62, 95% CI, 1.15 to 2.28; p<0.05  *Middle school*  AOR=3.76, 95% CI, 1.21 to 11.71; p<0.05  *High school*  AOR=1.49, 95% CI, 1.07 to 2.07; p<0.05 | NR |
| Blot et al., 2011 (24)  None  Aged 40-79 living in southern U.S. states  Fair (16): reporting (9); external validity (1); internal validity/bias (3); internal validity confounding (2); power (1) | Independent;  Logistic regression;  Age, gender, body mass index, annual household income, education, recruitment source, pack-years of smoking | NR | NR | NR | Former smoker: quit prior to enrollment  OR (95% CI) for M (ref=NM)  White  1.55 (1.41 to 1.70)  Black  1.03 (0.96 to 1.11)  Quit rate: quitting during follow-up (mean 4.3 years)  OR (95% CI) for M (ref=NM) | NR | NR |
| Cropsey et al., 2009 (31)  None  Female prisoners  Fair (17): reporting (9); external validity (0); internal validity/bias (5); internal validity confounding (3); power (0) | Independent;  GEE;  Age, race, education, CPD, change in smoking behavior since coming to prison, prior mental health treatment | NR | NR | NR | eCO-verified 7-day PPA  Wald chi-square=1.2  (p=0.27) | NR | NR |
| Cubbin et al., 2010 (2)  NHIS-CCS  None  Good (20): reporting (9); external validity (1); internal validity/bias (6); internal validity confounding (3); power (1) | Independent;  Prediction estimates;  Age, education, income; multiple gender and race stratifications | Time since quitting (years) among former smokers (≥100 lifetime cigs but no current smoking)  Comparing duration of abstinence by cigarette type among the 6 demographic groups, there was a statistically significant difference only among White women:  M: 14.8 years  NM: 12.5 years  (P<0.01)  For the other interactions, the duration of abstinence was not significant:  White men:  M: 14.6 years  NM: 13.9 years  Black women:  M: 13.0 years  NM: 12.2 years  Black men:  M: 11.7 years  NM: 10.6 years  Hispanic Women:  M: 13.8 years  NM: 12.2 years  Hispanic men:  M: 14.2 years  NM: 14.6 years | Past-year quit attempts  There were no statistically significant differences by race/ethnicity, gender or M/NM cigarette type:  Black women  M: 49.9%  NM: 39.7%  Black men  M: 48.6%  NM: 37.6%  Hispanic women  M: 46.7%  NM: 42.5%  Hispanic men:  M: 57.9%  NM: 38.4%  White women  M: 43.2%  NM: 39.8%  White men  M: 40.7%  NM: 36.8% | NR | NR | NR | NR |
| D'Silva et al., 2012 (37)  None  None  Fair (17): reporting (9); external validity (0); internal validity/bias (3; internal validity confounding (4); power (1) | Independent;  Multiple logistic regression;  Age, gender, race/ethnicity, education, marital status, CPD, TTFC, mental health status, confidence in quitting, and cessation methods | NR | NR | NR | 30-day PPA: quit for the past 30 days at the 7-month follow-up visit  OR (95% CI) for M (ref=NM)  1.29 (0.77 to 2.15) | NR | NR |
| Delnevo et al., 2010; Delnevo et al., 2011 (22, 23), 2010  TUS-CPS  None  Good (20): reporting (9); external validity (1); internal validity/bias (6); internal validity confounding (3); power (1) | Independent;  Multiple logistic regression;  Age, gender, race/ethnicity (overall only), income, education, year, month | NR | NR | NR | OR of being a former smoker (95% CI) for M (ref=NM)  Sample Restriction 1 (Cigarette smokers and former smokers who quit in the past 5 years)  *Total sample^a^:*  0.914 (0.868 to 0.961)  *White:*  0.928 (0.877 to 0.982)  *Black:*  0.810 (0.670 to 0.979)  *Hispanic:*  0.936 (0.793 to 1.105)  *Mexican:*  1.288 (0.999 to 1.661)  *Puerto Rican:*  0.569 (0.371 to 0.874)  Sample Restriction 2 (Cigarette smokers and former smokers who quit in the past 5 years who do not currently use other tobacco products):  *Total sample^a^:*  0.923 (0.876 to 0.973)  *White:*  0.943 (0.891 to 0.999)  *Black:*  0.781 (0.640 to 0.952)  *Hispanic:*  0.958 (0.810 to 1.113)  *Mexican:*  1.338 (1.039 to 1.722)  *Puerto Rican:*  0.630 (0.403 to 0.984)  Sample Restriction 3 (Cigarette smokers and former smokers who quit in the past 5 years who have made a quit attempt):  *Total sample^a^:*  0.902 (0.855 to 0.953)  *White:*  0.937 (0.882 to 0.994)  *Black:*  0.716 (0.585 to 0.875)  *Hispanic:*  0.881 (0.733 to 1.059)  *Mexican:*  1.302 (0.978 to 1.733)  *Puerto Rican:*  0.541 (0.344 to 0.849)  Sample Restriction 4 (Cigarette smokers and former smokers who quit in the past 5 years who have made a quit attempt and do not currently use other tobacco products):  *Total sample^a^:*  0.911 (0.862 to 0.964))  *White:*  0.952 (0.897 to 1.011)  *Black:*  0.684 (0.555 to 0.844)  *Hispanic:*  0.901 (0.749 to 1.084)  *Mexican:*  1.349 (1.016 to 1.790)  *Puerto Rican:*  0.590 (0.368 to 0.947)  Sample Restriction 5 (past-year smokers)*  *Total sample^a^:*  0.922 (0.847 to 1.004)  *White:*  0.982 (0.894 to 1.079)  *Black:*  0.740 (0.558 to 0.981)  *Hispanic:*  0.725 (0.543 to 0.969)  *Mexican:*  1.200 (0.778 to 1.852)  *Puerto Rican:*  0.421 (0.209 to 0.851)  *Additional adjustment for past-year cigarette tax increase | NR | NR |
| Fagan et al., 2007 (10)  TUS-CPS  Young adults  Fair (19): reporting (8); external validity (1); internal validity/bias (6); internal validity confounding (3); power (1) | Independent;  Logistic regression;  Age, gender, and independent variables in the bivariate models with P <0.25 (specific variables NR explicitly but appear to be age, gender, ethnicity, employment, age of smoking onset, CPD, TTFC) | NR | Stopping smoking “for 1 day or longer [in the past year]:  OR (95% CI) for M (ref=NM):  All current smokers  1.00 (0.89 to 1.16)  Daily smokers  1.00 (0.85 to 1.18)  Non-daily smokers  0.93 (0.62 to 1.41)  Stopping smoking “for 1 day or longer [in the past year] because you were trying to quit”:  Non-daily smokers  1.35 (0.60 to 3.03) | NR | NR | NR | NR |
| Faseru et al., 2013 (28)  None  Black light smokers (≤10 CPD)  Good (22): reporting (9); external validity (1); internal validity/bias (7); internal validity confounding (4); power (1) | Independent;  Multiple logistic regression;  cessation intervention, visit attendance, blood cotinine, years smoked | NR | NR | NR | Cotinine-verified 7-day PPA (Faseru, 2013)  OR (95% CI) for NM (ref=M)  At end of 7-week cessation intervention (bupropion vs. placebo):  1.84 (1.01 to 3.36) | NR | NR |
| Foulds et al., 2006 (34)  None  None, but includes ages 14 and older  Good (20): reporting (9); external validity (1); internal validity/bias (5); internal validity confounding (4); power (1) | Independent;  Logistic regression;  Age at baseline, education, employment, TTFC, baseline stage of change, number of contacts with cessation clinic | NR | NR | NR | 7-day PPA  At 4 weeks  OR (95% CI) for NM (ref=M)  1.36 (1.0 to 1.86)  (p=0.053) | NR | NR |
| Fu et al., 2008 (33)  None  VA patients  Good (21): reporting (9); external validity (2); internal validity/bias (5); internal validity confounding (4); power (1) | Independent;  Logistic regression;  Intervention vs. usual care, age, gender, race, TTFC, history of smoking-related cancer, history of substance use disorder, additional quit attempts in past 12 months | NR | NR | NR | Self-reported 7-day PPA  OR (95% CI) for M (ref=NM)  1.31 (0.95 to 1.82) | NR | NR |
| Gandhi et al., 2009 (32)  None  None  Fair (18): reporting (8); external validity (1); internal validity/bias (5); internal validity confounding (4); power (0) | Independent;  Logistic regression;  Age, gender, education, employment, presence of a disease caused or aggravated by smoking, health insurance, CPD, age smoked for first time, awaken at night to smoke, TTFC, previous quit attempts | NR | NR | NR | Self-reported 7-day PPA  OR (95% CI) for M (ref=NM)  At 4 weeks  *White*  0.96 (0.72 to 1.20)  *Black*  0.32 (0.16 to 0.62)  *Hispanic*  0.43 (0.1 to 0.9)  At 6 months  *White*  1.0 (0.8 to 1.4)  *Black*  0.48 (0.25 to 0.9)  *Hispanic*  0.64 (0.2 to 1.80) | NR | NR |
| Gubner et al., 2018 (44)  None;  Individuals with substance use disorders  Fair (19): reporting (8); external validity (3); internal validity/bias (4); internal validity confounding (3); power (1) | Independent;  Logistic regression;  Age, sex, race/ethnicity, education, primary drug, general health, days with poor mental health, CPD, and interest in getting help to quit smoking. | NR | NR | NR | NR | No difference in CPD between M and NM: OR=1.01 (95% CI: 0.98, 1.00); p=0.48 | NR |
| Gundersen et al., 2009 (25)  NHIS-CCS  None  Good (20): reporting (9); external validity (1); internal validity/bias (6); internal validity confounding (3); power (1) | Independent;  Logistic regression;  Age, gender, race (total sample only), education, U.S. census region, perceived risk of cancer, CPD | NR | NR | NR | Former smoker: having smoked 100 cigarettes in a lifetime and now smoking “not at all”  OR (95% CI) for M (ref=NM)  Total sample  1.05 (0.92 to 1.21); p=0.47  White:  1.17 (1.00 to 1.36); p<0.05  Black:  0.78 (0.56 to 1.09); p=0.15  Hispanic:  0.61 (0.39 to 0.97); p=0.04  Non-White (combined Black and Hispanic):  0.55 (0.43 to 0.71); p<0.01 | NR | NR |
| Hyland & Rivard, 2010; Hyland et al., 2002 (16, 17)  None  None  Good (23): reporting (9); external validity (2); internal validity/bias (7); internal validity confounding (4); power (1) | Varied by outcome;  Logistic regression;  Age, gender, race/ethnicity (total sample only), education, frequency of alcohol use, CPD, TTFC, age started smoking, desire to quit, past attempts to quit, use of non-cig tobacco product, other smokers in the house, desire to quit | NR | Quit attempts 2001-2005 in exclusively M/NM smokers 1988-2001 (Hyland and Rivard, 2010)  OR (95% CI) for M (ref=NM):  Total sample  0.91 (0.72 to 1.15)  White  0.91 (0.71 to 1.17)  Black  1.24 (0.27 to 5.67) | NR | Quit rate: “No” response to “Do you smoke now?” and “Have you smoked any cigarettes in the last 6 months?”  RR (95% CI) for M (ref=NM)  In 1993 for exclusively M/NM smokers at baseline (1988 (Hyland 2002)  *Total sample*  1.00 (0.90 to 1.11)  *White*  0.94 (0.83 to 1.05)  *Black*  1.04 (0.73 to 1.47)  *Hispanic*  1.22 (0.80 to 1.87)  In 2005 for exclusively M/NM smokers 1988-2001; OR (95% CI) for M (ref=NM) (Hyland and Rivard, 2010)  *Total sample*  0.84 (0.61 to 1.15)  *White*  0.79 (0.56 to 1.11)  In 2005 for exclusively M/NM smokers 1988-2001, with past quit attempts: OR (95% CI) for M (ref=NM) (Hyland and Rivard, 2010)  *Total sample*  1.03 (0.71 to 1.48)  *White*  0.96 (0.65 to 1.41) | Change in CPD between 1988 and 1993 among continuing smokers, β-coefficient (95% CI) for M (ref=NM) (Hyland, 2002)  [No association found]  Overall  0.11 (-0.38 to 0.60)  White  0.00 (-0.56 to 0.56)  Black  0.47 (-1.4 to 2.3)  Hispanic  1.16 (-1.3 to 3.6)  Decreasing CPD by exclusively M/NM smokers 1988-2001 among continuing smokers,  OR (95% CI) for M (ref=NM) (Hyland and Rivard, 2010):  Total sample  0.83 (0.64 to 1.07)  White  0.83 (0.63 to 1.09)  Black  0.14 (0.02 to 1.19) | NR |
| Kahende et al., 2011 (8)  TUS-CPS  None  Fair (19): reporting (8); external validity (1); internal validity/bias (6); internal validity confounding (3); power (1) | Independent;  Multiple logistic regression;  Age, gender, education, U.S. region, CPD, smoking duration, smoking policy at work, and main effects for race/ethnicity and home smoking rule, doctor’s advice to quit, TTFC | NR | Quit attempt in the past year OR (95% CI) for M (ref=White NM)  White  0.91 (0.84 to 0.99) | NR | NR | NR | NR |
| Kasza et al., 2014 (13) ITC-4 (U.S. data only)  None  Fair (19): reporting (8); external validity (2); internal validity/bias (5); internal validity confounding (3); power (1) | Independent;  GEE;  Age group, gender, race/ethnicity, education level, income level, HSI index, intention to quit (all measured at the same time as the outcome), time in sample, instrument change | NR | Quit attempt in M/NM type switchers,  OR (95% CI) for M (ref=NM):  Among those who switched from NM to M  *During the interval in which they switched:*  1.12 (0.80 to 1.57)  *Subsequent to the interval during which they switched:*  0.91 (0.57 to 1.44)  Among those who switched from M to NM  *During the interval in which they switched:*  1.09 (0.78 to 1.52)  *Subsequent to the interval during which they switched:*  1.03 (0.66 to 1.60) | NR | NR | NR | NR |
| Keeler et al., 2017 (3)  TUS-CPS;  None  Fair (19): reporting (8); external validity (3); internal validity/bias (4); internal validity confounding (3); power (1) | Independent;  Multiple logistic regression;  Gender, age, education, family income, marital status, census region of residence, and survey year (2006/2007, and 2010/2011). | NR | No difference in past-year quit attempt between M and NM smokers:  OR=0.99 (0.94-1.04); p=0.6690  Among Black smokers, odds of past-year quit attempts were statistically significantly higher in M smokers versus NM smokers:  OR=1.37, 95% CI: 1.16–1.61; p=0.0002  Among White smokers, no difference in odds of past-year quit attempts between M and NM smokers:  OR=0.97, 95% CI: 0.91–1.02; p=0.2450)  Among Asian smokers, no difference in odds of past-year quit attempts between M and NM smokers:  OR=0.91, 95% CI: 0.62–1.34; p=0.6470)  Among Hispanic smokers, no difference in odds of past-year quit attempts between M and NM smokers:  OR=1.09, 95% CI: 0.91–1.30; p=0.3540) | NR | No difference in rate of abstinence (≥3 months) between M and NM smokers:  OR=0.92 95% CI: 0.83–1.03; p=0.1470  Among Black smokers, no difference in odds cessation ((≥3 months) between M and NM smokers:  OR=1.03, 95% CI: 0.73–1.44; p=0.8630  Among White smokers, no difference in odds cessation ((≥3 months) between M and NM smokers:  OR=0.94, 95% CI: 0.84–1.06; p=0.3190  Among Asian smokers, no difference in odds cessation ((≥3 months) between M and NM smokers:  OR=0.98, 95% CI: 0.44–2.19; p=0.9540  Among Hispanic smokers, no difference in odds cessation ((≥3 months) between M and NM smokers:  OR=0.88, 95% CI: 0.60–1.28; p=0.4980 | NR | NR |
| Keeler et al., 2018 (4)  TUS-CPS;  Subsamples of African-American and White respondents, respectively.  Fair (19): reporting (8); external validity (3); internal validity/bias (4); internal validity confounding (3); power (1) | Independent;  Multiple logistic regression;  Gender, age, education, family income, marital status, smoking intensity, menthol use status, how soon to smoke the first cigarette after awakening, and survey year (2006–2007 and 2010–2011). | NR | Among Black smokers, M smokers were statistically significantly more likely to report any past-year quit attempts, compared to NM smokers:  M=48.9% vs NM=40.8%; OR=1.39 (1.16-1.67); p<0.001  Among White smokers, past-year quit attempts did not differ between M and NM smokers:  M=40.6% vs NM=40.7%; OR=0.95 (0.89-1.01); p=NS | NR | Among Black smokers, successful cessation (≥3 months) did not differ between menthol and non-menthol smokers:  M=5.1% vs. NM=5.6%; OR=1.01 (0.70-1.45); p=NS  Among White smokers, successful cessation (≥3 months) did not differ between menthol and non-menthol smokers:  M=5.8% vs. NM=6.2%; OR=0.94 (0.84-1.07); p=NS | NR | NR |
| Levy et al., 2011 (1)  TUS-CPS  None  Good (20): reporting (9); external validity (1); internal validity/bias (6); internal validity confounding (3); power (1) | Independent;  Multiple logistic regression;  Age, gender, race/ethnicity, marital status, education, family income, indicator if income >$175000, state smoke-free policies, survey wave^b^ | Among all former smokers (≥100 lifetime cigarettes but not currently smoking)  OR (95% CI) for M (ref=NM)  Quit ≥3 months and ≤1 year at interview:  0.97 (0.96 to 0.97)  Quit ≥3 months and ≤5 years at interview:  0.94 (0.94 to 0.94) | Quit attempt in the past year among current smokers 1 year prior to interview  OR (95% CI) for M (ref=NM)  1.03 (1.02 to 1.03) | NR | NR | NR | NR |
| Lewis et al., 2014 (19)  None  None  Fair (19): reporting (9); external validity (0); internal validity/bias (6); internal validity confounding (3); power (1) | Independent;  Hazard ratio (Cox);  Age, gender, race/ethnicity, income, recency, nicotine intake, cig quality preference, price+tax, anti-smoking advertising, smoke-free policies | NR | NR | NR | Former smoker: did not purchase a pack of cigarettes for ≥1 year  HR (95% CI) for M (ref= NM)  0.79 (0.64 to 0.99) | NR | NR |
| Muench and Juliano, 2017 (47)  None;  None  Good (21): reporting (9); external validity (3); internal validity/bias (4); internal validity confounding (4); power (1) | Independent;  Binary logistic regression, proportional hazards regression  Age, gender, income, education level, and race/ethnicity. | NR | NR | NR | NR | NR | A preference for menthol cigarettes was associated with greater lapse risk:  OR=3.474, p<0.05  Menthol preference is associated with a statistically significant higher risk of lapsing within the first 48 hours of abstinence, compared with those who are non-menthol users:  HR=2.798, Wald statistic=2.79 (p=0.48) |
| Muscat et al., 2002 (21)  None  Cancer patients and non-cancer controls  Fair (19): reporting (9); external validity (0); internal validity/bias (6); internal validity confounding (3); power (1) | Independent;  Logistic regression;  Age, gender, education, case-control status, years of smoking, CPD | NR | NR | NR | Current smoking (vs. former smoker):  Prevalence OR (95% CI) for M (ref=NM) White  1.1 (1.0 to 1.3)  Black  1.1 (0.8 to 1.4)  Menthol β (SE) total sample  0.11; (0.06) | NR | NR |
| Nonnemaker et al., 2012 (26)  ALLTURS  Youth;  Good (22): reporting (9); external validity (2); internal validity/bias (6); internal validity confounding (4); power (1) | Independent;  Competing risk survival analysis;  Age, gender, race/ethnicity | NR | NR | NR | Quit rate: transition from non-established smoking to non-smoking^c^ for those who initiated with M:  OR (95% CI) for M (ref= NM)  1.18 (0.78 to 1.80) | NR | NR |
| Okuyemi et al., 2003 (35)  None  Blacks  Good (24): reporting (9); external validity (1); internal validity/bias (7); internal validity confounding (6); power (1) | Independent;  Logistic regression;  Treatment arm and "the other baseline variables" (appear to be gender, income, education, employment, CPD, FTND, TTFC, taste/satisfaction, cotinine, eCO) | NR |  |  | 7-day PPA (Okuyemi, 2003)  OR (95% CI) for NM (ref=M);  At 6 weeks:  *Age <50 years*  2.02 (1.03 to 3.95)  *Age ≥50 years*  “Not significant” |  | NR |
| Okuyemi et al., 2007 (36)  None  Black light smokers (≤10 CPD)  Good (25): reporting (9); external validity (2); internal validity/bias (7); internal validity confounding (6); power (1) | Independent;  Logistic regression;  Drug and counseling treatment assignments, confidence to quit smoking | NR | NR | NR | Cotinine-verified 7-day PPA: not having smoked even a puff for past 7 days ([Okuyemi et al., 2007a](#_ENREF_68))  At 26 weeks  *Age <50 years*  2.077 (0.944 to 4.569); p=0.069  *Age ≥50 years*  1.676 (0.760 to 3.698); p=0.221  “No significant interaction between categorized age (< 50 versus ≥50) and menthol status on 7-day PPA at week 26 (P=0.93)” | NR | NR |
| Park, 2017 (5)  National Adult  Tobacco Survey (NATS);  None;  Good (20): reporting (9); external validity (3); internal validity/bias (4); internal validity confounding (3); power (1) | Independent;  Binary logistic regression;  Race/ethnicity, age, and gender. |  | Likelihood of past-year quit attempts, M vs NM (ref):  OR=1.19 (95%CI:0.97-1.46); p=0.92 | NR |  | NR |  |
| Pletcher et al., 2006 (14)  None  Young adults  Good (20): reporting (9); external validity (2); internal validity/bias (4); internal validity confounding (4); power (1) | Independent;  For cross-sectional outcomes: related-measures logistic models;  For longitudinal outcomes: logistic regression with 1 observation per participant;  Age, gender, race/ethnicity, social factors (educational level, marital status, employment, health insurance status), CPD at baseline^d^ | NR | OR (95% CI) for a "recent quit attempt" for M (ref=NM):  0.77 (0.57-1.06) | NR | Quit rate: "not currently smoking" at any examination  OR (95% CI) for M (ref=NM)  Total sample  0.90 (0.68 to 1.19)  Participants with recent quit attempt  1.00 (0.71 to 1.42)  Rate of "sustained smoking cessation" (no current smoking at the past 2 examinations)  OR (95% CI) for M (ref=NM)  0.71 (0.49 to 1.02) | NR | Documented relapse: baseline smokers who reported no current smoking at a subsequent examination but reported current smoking at the final examination  OR (95% CI) for M (ref=NM):  1.89 (1.17 to 3.05) |
| Rath et al., 2015 (12)  LYAC  Young adults  Fair (17): reporting (9); external validity (1); internal validity/bias (4); internal validity confounding (3); power (0) | Dependent;  Multiple logistic regression;  Age, gender, race/ethnicity, self-described financial situation | NR | OR (95% CI) for M (ref=NM):  Ever quit attempt (ref=never attempted):  0.84 (0.43 to 1.63)  Quit attempt in past 6 months (ref= none):  0.62 (0.30 to 1.27) | NR | NR | NR | NR |
| Reitzel, 2011a (40)  None  Blacks  Fair (19): reporting (9); external validity (0); internal validity/bias (6); internal validity confounding (4); power (0) | Independent;  CR logit;  Age, gender, partner status, income, employment status, educational achievement, time, treatment group, CPD, TTFC | NR | NR | NR | Prolonged abstinence: no smoking (not even a puff) since the quit date, verified by eCO <10 ppm or, in a few cases where participants were unable to attend more distal study visits, a mailed saliva cotinine <20 ng/ml, assessed at 3 days, 10 days, 31 days, and 26 weeks post-quit:  Menthol β (SE);  0.33 (0.32)  (p=0.30) | NR | NR |
| Reitzel, 2011b (39)  None  None  Fair (18): reporting (8); external validity (0); internal validity/bias (6); internal validity confounding (4); power (0) | Independent;  CR logit;  Age, gender, race/ethnicity, partner status, annual household income, employment status, educational achievement, time, CPD, TTFC | NR | NR | NR | Prolonged abstinence: no smoking (not even a puff) since the quit date and biochemical confirmation at 1, 2, 4, and 26 weeks post-quit (eCO <10 ppm):  Menthol β (SE);  0.05 (0.25)  (p=0.84) | NR | NR |
| Reitzel, 2011c; Reitzel et al., 2011 (41, 42)  None  Pregnant women  Good (21): reporting (9); external validity (0); internal validity/bias (7); internal validity confounding (5); power (0) | Independent;  CR logit  Model 2: age, race/ethnicity, partner status, income, educational achievement, time, treatment group, CPD, TTFC | NR | NR | NR | Prolonged abstinence: no smoking (not even a puff) since the delivery date and biochemical confirmation at 8 and 26 weeks postpartum (eCO <10 ppm and/or cotinine <20 ng/ml) (Reitzel, 2011; Reitzel 2011c)  Menthol cigarette use did not statistically significantly predict continuous abstinence from smoking through 26 weeks postpartum: β=−0.32, SE=0.30; p=0.29; n=297 ([Reitzel et al., 2011](#_ENREF_81))  White menthol users were less likely to maintain continuous abstinence through post-quit week 26 than White non-menthol users: β=-1.62, SE=0.76; p=0.03; n=108, OR=0.19 (0.04-0.89). (41, 42)  No significant difference in continuous abstinence between menthol and non-menthol use was found among Black women (b=−1.12, SE=.64; c2=3.06; p=.08; n=96) nor Latina women (b=.46, SE=.50; c2=.86; p=.35; n=93) ([Reitzel et al., 2011](#_ENREF_81)). | Menthol cigarette use was not statistically significantly associated with the number of CPD (β=-0.38, SE=1.15; t=-.33; p=.74; n=222) ([Reitzel, 2011c](#_ENREF_78))  Black menthol users smoked statistically significant more CPD than Black non-menthol users: β=3.82, SE=3.77; p=0.02; n=71; effect size=0.67. ([Reitzel, 2011c](#_ENREF_78)) | NR |
| Reitzel et al., 2013 (38)  None  None  Fair (19): reporting (9); external validity (0); internal validity/bias (5); internal validity confounding (4); power (0) | Independent;  CR logit;  Age, gender, race, partner status, income, educational level, employment status, stage | NR | NR | NR | Prolonged abstinence: no smoking (not even a puff) since the quit date and eCO <10ppm  Menthol β (SE)  Full sample  -0.31 (0.40); (p=0.44)  White  -1.56 (0.79); (p=0.05)  Black  0.54 (0.55); (p=0.33)  7-day PPA through post quit week 3  Menthol β (SE)  White  -1.90 (0.82); (p=0.02)  Black  1.00 (0.67); (p=0.11) | NR | NR |
| Rojewski et al., 2014 (27)  None  None  Good (22): reporting (9); external validity (0); internal validity/bias (7); internal validity confounding (6); power (0) | Independent;  Logistic regression;  Age | NR | NR | NR | eCO-verified 7-day PPA  CI) for NM (ref=M)  At week 14  2.40 (1.04 to 5.55)  At week 26  2.47 (1.04 to 5.90) | NR | NR |
| Sawdey et al., 2020 (46)  NYTS  Middle and high school students (grades 6-12)  Good (20): reporting (9); external validity (3); internal validity/bias (4); internal validity confounding (3); power (1) | Dependent;  Multivariable logistic regression;  Gender, school level, race/ethnicity, CPD, current non-cigarette tobacco use (by flavor use status), household tobacco use, believe cigarettes are harmful, craving tobacco | NR | NR | NR | NR | Odds of participants who smoked 6-19 days in past 30 (moderate) (1-5 days in past 30 reference) to be menthol users versus non-menthol:  AOR=1.17, 95% CI: 0.86-1.59;  Odds of participants who smoked ≥20 days in past 30 (frequent) (1-5 days in past 30 reference) to be menthol users versus non-menthol:  AOR=1.57, 95% CI: 1.08-2.29;  *Overall p-value across 2 groups: p=0.064 | NR |
| Schneller et al., 2020; Schneller, 2020 (6, 7)  PATH  None  Fair (19): reporting (8); external validity (3); internal validity/bias (4); internal validity confounding (3); power (1) | For Any Quit Attempt  Independent;  Multinomial logistic regression;  Gender, age, race/ethnicity, education, and HSI  For Rate of Abstinence/Quitting  Schneller et al., 2020(7): Independent;  Multinomial logistic regression;  Gender, age, race/ethnicity, education, and HSI  Schneller, 2020(6): Independent;  Multinomial logistic regression;  Gender, age, race/ethnicity, education, and CPD | NR | Adjusted risk of menthol users reporting a past 12 month quit attempt compared to non-menthol users:  RRR=1.00, 95% CI: 0.89-1.13, p=NS | NR | Schneller et al., 2020(7):  Adjusted odds of menthol users reporting successful cessation at Wave 2 compared to non-menthol users:  AOR=1.09, 95% CI: 0.88-1.37, p=NS  Schneller, 2020(6):  Adjusted risk of menthol users reporting successful cessation at Wave 2 compared to non-menthol users:  RRR=1.09, 95% CI: 0.87-1.35, p=NS | NR | NR |
| Stahre et al., 2010 (15)  NHIS-CCS  None  Fair (18): reporting (9); external validity (3); internal validity/bias (2); internal validity confounding (3); power (1) | Independent;  Logistic regression;  Age group, gender, ethnicity, U.S. region, marital status and average number of CPD | NR | Using a quit aid, OR (95% CI) for M (ref=NM):  Current smokers: 1.05 (0.80 to 1.36)  Former smokers: 1.29 (0.74 to 2.26) | NR | NR | NR | NR |
| Steinberg et al., 2011 (30)  None  None  Good (21): reporting (9); external validity (1); internal validity/bias (7; internal validity confounding (4); power (0) | Independent;  Logistic regression;  Age, gender, race, marital status, education, employment, CPD, TTFC, night smoking | NR | NR | NR | eCO-verified 7-day PPA  OR (95% CI) for M (ref=NM)  At 6 months  1.02 (0.66-1.58) | NR | NR |
| Sulsky et al., 2014 (20)  NHIS, TUS-CPS  None  Good (20): reporting (9); external validity (1); internal validity/bias (6); internal validity confounding (3); power (1) | Independent;  Logistic regression;  Varied by model; see footnotes | NR | NR | NR | Long-term former smoker vs. daily smoker^e^ (NHIS)  OR (95% CI) for M (ref=NM)  NH White^f^  *Model 1:*  1.06 (0.95 to 1.18)  NH Black^g^  *Model 1:*  1.04 (0.82 to 1.33)  Short-term former smoker vs. regular smoker^e^ (TUS-CPS)  OR (95% CI) for M (ref=NM)  NH White^h^  *Model 1:*  0.97 (0.94 to 1.00)  NH Black^i^  *Model 1:*  0.87 (0.80 to 0.95)  Other race/ethnicity^i^  *Model 1:*  0.99 (0.91 to 1.08)  Short-term former smoker vs. daily smoker^e^ (TUS-CPS)  OR (95% CI) for M (ref=NM)  NH White^h^  *Model 1:*  0.98 (0.95 to 1.01)  NH Black^i^  *Model 1:*  0.89 (0.81 to 0.98)  Other race/ethnicity^i^  *Model 1:*  1.00 (0.92 to 1.09) | NR | NR |
| Thihalolipavan et al., 2014 (43)  None  None  Poor (11): reporting (5); external validity (0); internal validity/bias (4); internal validity confounding (1); power (1) | Independent;  Poisson regression;  Model 1: Age, gender, race/ethnicity, education, income, Medicaid status, CPD  Model 2: Model 1 plus previous quit attempt status | NR | NR | NR | Quit rate (definition NR)  PR (95% CI) for M (ref=NM)  At 3-6 weeks post-baseline:  *Model 3:*  0.90 (0.83 to 0.97)  *Model 4:*  0.90 (0.83 to 0.97) | NR | NR |
| Trinidad et al., 2010 (18)  TUS-CPS  None  Good (21): reporting (9); external validity (1); internal validity/bias (6); internal validity confounding (4); power (1) | Independent;  Logistic regression;  Age, gender, education, current use of other tobacco products | NR | NR | NR | Successful quitting ≥6 months among former smokers  OR (95% CI) for M (ref=NM)  NH White  0.28 (0.25 to 0.33)  Black  0.23 (0.17 to 0.31)  Asian-Americans/Pacific Islander  0.22 (0.11 to 0.45)  Native American/Alaskan Native  0.49, (0.14, 1.71)  Hispanic/Latino  0.48 (0.34 to 0.69) | NR | NR |
| Webb Hooper et al., 2011 (9)  BRFSS  None  Good (20): reporting (9); external validity (1); internal validity/bias (5); internal validity confounding (4); power (1) | Dependent;  Logistic regression;  Age, gender, race/ethnicity, ever had a medical condition, frequency poor physical health days, have health care coverage, pack-years, dependence | NR | Past-year quit attempt  OR (95% CI) for M (ref=NM):  0.96 (0.81 to 1.15) | NR | NR | NR | NR |
| Winhusen et al., 2013 (29)  None  Cocaine or methamphetamine addicted smokers  Fair (18): reporting (9); external validity (0); internal validity/bias (7); internal validity confounding (2); power (0) | Independent;  Logistic regression;  Age, gender, race, stimulant-positive urine drug screen, stimulant use-route, FTND, duration of time as a smoker, CPD | NR | NR | NR | 7-day PPA (at week 10)  Cocaine-dependent  p=0.81  Methamphetamine-dependent  p=0.9 | NR | NR |

^a^Former smokers who quit within the past 5 years and all current smokers (regardless of quit attempt history). Results were also reported for more restrictive populations (e.g., those with past quit attempts, those who do not use other tobacco products); results were similar to those presented here.

^b^Results were also reported from a model that contained CPD and TTFC; results were similar.

^c^ Established smoking was defined in each wave as smoked ≥100 lifetime cigarettes and smoked on 20–30 of the past 30 days.

^d^Results were reported also from models with fewer covariates; results were similar.

^e^Former smoker: ≥100 lifetime cigarettes but no smoking within the past year. Short-term former smoker: ≥100 lifetime cigarettes and abstained from smoking for 1-3 years. Long-term former smoker: ≥100 lifetime cigarettes but no smoking during the past year; duration of quitting ranged from 1 to >70 years, and average duration of quitting was 18 years. Regular smoker: ≥100 lifetime cigarettes and smoked on ≥10 days during the past month.

^f^Model 1 adjusted for CPD and current age; model 2 adjusted for CPD and duration of smoking

^g^Model 1 adjusted for CPD, initiation age, and current age; model 2 adjusted for CPD, initiation age, and duration of smoking

^h^Model 1 adjusted for age and HSI; model 2 adjusted for current age and night waking to smoke.

^i^Model 1 adjusted for HSI; model 2 adjusted for night waking to smoke.

Abbreviations: ALLTURS=American Legacy Longitudinal Tobacco Use Reduction Study; AOR=adjusted odds ratio; BRFSS=Behavioral Risk Factor Surveillance System; cig=cigarette; CI=confidence interval; eCO=exhaled carbon monoxide; CPD=cigarettes per day; CR=continuation ratio; FTND=Fagerström Test for Nicotine Dependence; GEE=generalized estimating equation; HR=Hazard Ratio; HSI=heaviness of smoking index; ITC-4=International Tobacco Control Four Country Survey (U.S. data only); LYAC=Legacy Young Adult Cohort; M=menthol; NH=non-Hispanic; NHIS=National Health Interview Survey; NH=non-Hispanic; NHIS-CCS=National Health Interview Survey Cancer Control Supplement; NM=non-menthol; NR=not reported; NS=not significant; NYTS=National Youth Tobacco Survey; OR=odds ratio; PATH=Population Assessment of Tobacco and Health; PPA=point prevalence abstinence; PR=prevalence ratio; ref=reference; RRR=relative risk ratio; SE=standard error; TTFC=time to first cigarette of the day; TUS-CPS=Tobacco Use Supplement to the Current Population Survey; VA=Veterans Health Administration.

**References**

1. Alexander LA, Crawford T, Mendiondo MS. Occupational status, work-site cessation programs and policies and menthol smoking on quitting behaviors of US smokers. Addiction. 2010;105 Suppl 1:95-104.

2. Azagba S, King J, Shan L, Manzione L. Cigarette Smoking Behavior Among Menthol and Nonmenthol Adolescent Smokers. J Adolesc Health. 2020;66(5):545-50.

3. Blot WJ, Cohen SS, Aldrich M, McLaughlin JK, Hargreaves MK, Signorello LB. Lung cancer risk among smokers of menthol cigarettes. J Natl Cancer Inst. 2011;103(10):810-6.

4. Cropsey KL, Weaver MF, Eldridge GD, Villalobos GC, Best AM, Stitzer ML. Differential success rates in racial groups: results of a clinical trial of smoking cessation among female prisoners. Nicotine Tob Res. 2009;11(6):690-7.

5. Cubbin C, Soobader MJ, LeClere FB. The intersection of gender and race/ethnicity in smoking behaviors among menthol and non-menthol smokers in the United States. Addiction. 2010;105 Suppl 1:32-8.

6. D'Silva J, Boyle RG, Lien R, Rode P, Okuyemi KS. Cessation outcomes among treatment-seeking menthol and nonmenthol smokers. Am J Prev Med. 2012;43(5 Suppl 3):S242-8.

7. Delnevo CD, Gundersen DA, Hrywna M. Examining the relationship between menthol smoking and cessation using data from the 2003 and 2006/7 Tobacco Use Supplement. Center for Tobacco Surveillance and Evaluation Research: University of Medicine & Dentistry of New Jersey - School of Public Health; 2010 January 10-11, 2011.

8. Delnevo CD, Gundersen DA, Hrywna M, Echeverria SE, Steinberg MB. Smoking-cessation prevalence among U.S. smokers of menthol versus non-menthol cigarettes. Am J Prev Med. 2011;41(4):357-65.

9. Fagan P, Augustson E, Backinger CL, O'Connell ME, Vollinger RE, Jr., Kaufman A, et al. Quit attempts and intention to quit cigarette smoking among young adults in the United States. Am J Public Health. 2007;97(8):1412-20.

10. Faseru B, Nollen NL, Mayo MS, Krebill R, Choi WS, Benowitz NL, et al. Predictors of cessation in African American light smokers enrolled in a bupropion clinical trial. Addict Behav. 2013;38(3):1796-803.

11. Foulds J, Gandhi KK, Steinberg MB, Richardson DL, Williams JM, Burke MV, et al. Factors associated with quitting smoking at a tobacco dependence treatment clinic. Am J Health Behav. 2006;30(4):400-12.

12. Fu SS, Okuyemi KS, Partin MR, Ahluwalia JS, Nelson DB, Clothier BA, et al. Menthol cigarettes and smoking cessation during an aided quit attempt. Nicotine Tob Res. 2008;10(3):457-62.

13. Gandhi KK, Foulds J, Steinberg MB, Lu SE, Williams JM. Lower quit rates among African American and Latino menthol cigarette smokers at a tobacco treatment clinic. Int J Clin Pract. 2009;63(3):360-7.

14. Gubner NR, Williams DD, Pagano A, Campbell BK, Guydish J. Menthol cigarette smoking among individuals in treatment for substance use disorders. Addictive behaviors. 2018;80:135-41.

15. Gundersen DA, Delnevo CD, Wackowski O. Exploring the relationship between race/ethnicity, menthol smoking, and cessation, in a nationally representative sample of adults. Prev Med. 2009;49(6):553-7.

16. Hyland A, Garten S, Giovino GA, Cummings KM. Mentholated cigarettes and smoking cessation: Findings from COMMIT. Tob Control. 2002;11:135-9.

17. Hyland A, Rivard C. Analysis of mentholated cigarettes using the COMMIT data -- summary. Department of Health Behavior, Roswell Park Cancer Institute; 2010.

18. Kahende JW, Malarcher AM, Teplinskaya A, Asman KJ. Quit attempt correlates among smokers by race/ethnicity. International journal of environmental research and public health. 2011;8(10):3871-88.

19. Kasza KA, Hyland AJ, Bansal-Travers M, Vogl LM, Chen J, Evans SE, et al. Switching between menthol and nonmenthol cigarettes: findings from the U.S. Cohort of the International Tobacco Control Four Country Survey. Nicotine Tob Res. 2014;16(9):1255-65.

20. Keeler C, Max W, Yerger V, Yao T, Ong MK, Sung H-Y. The Association of Menthol Cigarette Use With Quit Attempts, Successful Cessation, and Intention to Quit Across Racial/Ethnic Groups in the United States. Nicotine & tobacco research : official journal of the Society for Research on Nicotine and Tobacco. 2017;19(12):1450-64.

21. Keeler C, Max W, Yerger VB, Yao T, Wang Y, Ong MK, et al. Effects of cigarette prices on intention to quit, quit attempts, and successful cessation among African American smokers. Nicotine & tobacco research : official journal of the Society for Research on Nicotine and Tobacco. 2018.

22. Levy DT, Blackman K, Tauras J, Chaloupka FJ, Villanti AC, Niaura RS, et al. Quit attempts and quit rates among menthol and nonmenthol smokers in the United States. Am J Public Health. 2011;101(7):1241-7.

23. Lewis M, Wang Y, Berg CJ. Tobacco control environment in the United States and individual consumer characteristics in relation to continued smoking: differential responses among menthol smokers? Prev Med. 2014;65:47-51.

24. Muench C, Juliano LM. Predictors of smoking lapse during a 48-hour laboratory analogue smoking cessation attempt. Psychology of addictive behaviors : journal of the Society of Psychologists in Addictive Behaviors. 2017;31(4):415-22.

25. Muscat JE, Richie JP, Jr., Stellman SD. Mentholated cigaettes and smoking habits in whites and blacks. Tob Control. 2002;11:368-71.

26. Nonnemaker J, Hersey J, Homsi G, Busey A, Allen J, Vallone D. Initiation with menthol cigarettes and youth smoking uptake. Addiction. 2012;108(1):171-8.

27. Okuyemi KS, Ahluwalia JS, Ebersole-Robinson M, Catley D, Mayo MS, Resnicow K. Does menthol attenuate the effect of bupropion among African American smokers? Addiction. 2003;98:1387-93.

28. Okuyemi KS, Faseru B, Sanderson Cox L, Bronars CA, Ahluwalia JS. Relationship between menthol cigarettes and smoking cessation among African American light smokers. Addiction. 2007;102(12):1979-86.

29. Park J-Y. Tobacco use behaviors among vulnerable populations. Dissertation Abstracts International: Section B: The Sciences and Engineering. 2017;77(12-B(E)):No-Specified.

30. Pletcher MJ, Hulley BJ, Houston T, Kiefe CI, Benowitz N, Sidney S. Menthol cigarettes, smoking cessation, atherosclerosis, and pulmonary function. Arch Intern Med. 2006;166:1915-22.

31. Rath JM, Villanti AC, Williams VF, Richardson A, Pearson JL, Vallone DM. Patterns of Longitudinal Transitions in Menthol Use Among US Young Adult Smokers. Nicotine Tob Res. 2015;17(7):839-46.

32. Reitzel LR. Menthol cigarettes, tobacco dependence, and smoking cessation: Project BREAK FREE final report. 2011.

33. Reitzel LR. Menthol cigarettes, tobacco dependence, and smoking cessation: Project CARE final report. 2011.

34. Reitzel LR. Menthol cigarettes, tobacco dependence, and smoking cessation: Project MOM final report. 2011.

35. Reitzel LR, Nguyen N, Cao Y, Vidrine JI, Daza P, Mullen PD, et al. Race/ethnicity moderates the effect of prepartum menthol cigarette use on postpartum smoking abstinence. Nicotine Tob Res. 2011;13(12):1305-10.

36. Reitzel LR, Li Y, Stewart DW, Cao Y, Wetter DW, Waters AJ, et al. Race moderates the effect of menthol cigarette use on short-term smoking abstinence. Nicotine Tob Res. 2013;15(5):883-9.

37. Rojewski AM, Toll BA, O'Malley SS. Menthol cigarette use predicts treatment outcomes of weight-concerned smokers. Nicotine Tob Res. 2014;16(1):115-9.

38. Sawdey MD, Chang JT, Cullen KA, Rass O, Jackson KJ, Ali FRM, et al. Trends and Associations of Menthol Cigarette Smoking Among US Middle and High School Students-National Youth Tobacco Survey, 2011-2018. Nicotine Tob Res. 2020;22(10):1726-35.

39. Schneller LM. Assessment of various delivery methods of menthol in cigarettes sold in the US. Dissertation Abstracts International: Section B: The Sciences and Engineering. 2020;81.

40. Schneller LM, Bansal-Travers M, Mahoney MC, McCann SE, O'Connor RJ. Menthol Cigarettes and Smoking Cessation among Adult Smokers in the US. Am J Health Behav. 2020;44(2):252-6.

41. Stahre M, Okuyemi KS, Joseph AM, Fu SS. Racial/ethnic differences in menthol cigarette smoking, population quit ratios and utilization of evidence-based tobacco cessation treatments. Addiction. 2010;105 Suppl 1:75-83.

42. Steinberg MB, Bover MT, Richardson DL, Schmelzer AC, Williams JM, Foulds J. Abstinence and psychological distress in co-morbid smokers using various pharmacotherapies. Drug Alcohol Depend. 2011;114(1):77-81.

43. Sulsky SI, Fuller WG, Van Landingham C, Ogden MW, Swauger JE, Curtin GM. Evaluating the association between menthol cigarette use and the likelihood of being a former versus current smoker. Regul Toxicol Pharmacol. 2014;70(1):231-41.

44. Thihalolipavan S, Jung M, Jasek J, Chamany S. Menthol smokers in large-scale nicotine replacement therapy program. Am J Public Health. 2014;104(11):e3-4.

45. Trinidad DR, Perez-Stable EJ, Messer K, White MM, Pierce JP. Menthol cigarettes and smoking cessation among racial/ethnic groups in the United States. Addiction. 2010;105 Suppl 1:84-94.

46. Webb Hooper M, Zhao W, Byrne MM, Davila E, Caban-Martinez A, Dietz NA, et al. Menthol cigarette smoking and health, Florida 2007 BRFSS. Am J Health Behav. 2011;31(1):3-14.

47. Winhusen TM, Adinoff B, Lewis DF, Brigham GS, Gardin JG, 2nd, Sonne SC, et al. A tale of two stimulants: mentholated cigarettes may play a role in cocaine, but not methamphetamine, dependence. Drug Alcohol Depend. 2013;133(3):845-51.
