# Supplementary material for: Assessing the evidence on the differential impact of menthol versus non-menthol cigarette use on smoking cessation in the U.S. population: a systematic review and meta-analysis
Source: Subst Abuse Treat Prev Policy. 2021 Aug 11;16:61. doi: 10.1186/s13011-021-00397-4 (PMC8359586; doi:10.1186/s13011-021-00397-4)
Supplement: Supplementary file 5 — Additional file 5. Sensitivity Analyses. [file 13011_2021_397_MOESM5_ESM.docx]

**SUPPLEMENTAL SECTION 5: Sensitivity Analyses**

**SUPPLEMENTAL SECTION 5: Sensitivity Analyses**

Sensitivity analyses ensure that initial conclusions were not unduly influenced by data that may not have met certain standards or thresholds. In this review, three sensitivity analyses were conducted in order to test whether the results differed after more stringent inclusion and exclusion criteria were applied. The criteria for these analyses are detailed below:

1. *Limiting studies to those that also (i.e., in addition to adjusting for age, gender, and race/ethnicity) adjusted for meso- or macro-level variables.*

Epidemiological studies that adjusted for these additional factors have the potential ability to more rigorously isolate the independent effect of menthol on cessation.

1. *Exclusion of studies rated “poor” according to the Downs and Black checklist.*

Studies rated “poor” quality generally suffered from low external and/or external validity. Exclusion of such studies from the adjusted analyses provides more accurate results.

1. *Exclusion of studies with potential overadjustment and/or inappropriate adjustment.*

Overadjustment occurs when a statistical model includes a covariate that either increases bias or decreases precision without affecting bias (49). Therefore, studies with overadjustment or inappropriate adjustment may impact the SOE ratings of consistency and precision directly. Therefore, exclusion of such studies can alter the overall SOE results.

**Sensitivity Analysis 1. Limiting studies to those that also (i.e., in addition to adjusting for the age, gender, and race/ethnicity) adjusted for meso- or macro-level variables.**

*Nine included studies (from ten references) presented adjusted results that included meso- and/or macro-level variables in their models. Findings for all outcomes are either insufficient to suggest an additional effect on the association between menthol and the outcome, or they indicate that such factors do not affect such an association.*

Duration of Abstinence

One study that reported duration of abstinence included meso- and macro-level variables in its adjusted analyses. Levy et al. (1) controlled for state smoke-free policies and found that menthol was associated with slightly but statistically significant lower odds of being a recent or long-term quitter. Specifically, the odds of being a “recent quitter” were three percent lower for menthol smokers than non-menthol smokers (OR=0.97, 95% CI: 0.96, 0.97). Similarly, the odds of being a “longer-term quitter” were six percent lower for menthol smokers (OR=0.94, 95% CI: 0.94, 0.94).

Quit Attempts

*Five studies that reported quit attempts included meso- and/or macro-level variables. Two of the five studies found that menthol smokers were less likely than non-menthol smokers to have made a quit attempt; one found that menthol users were statistically significantly more likely to have made a past-year attempt; and two reported mixed results based on racial stratifications. These findings were consistent with the inconsistency found in the overall results.*

Alexander et al. (11) adjusted for the presence of smokers in the work space and for workplace smoking policies and restrictions and found that menthol smokers had 2 percent lower odds of stopping smoking for one day or more.

Hyland et al. (17) adjusted for pricing tier of cigarette smoked (premium, discount, generic) and the presence of another smoker in the household in their analysis of interim data from COMMIT. Exclusively menthol smokers were no different from non-menthol smokers in the odds of having made a quit attempt (AOR=0.91, 95% CI: 0.72, 1.15).

Kahende et al. (8) adjusted for region, smoking policy at work, and home smoking rules, and their analyses were stratified by race, with White non-menthol smokers always the reference group. Among White smokers, menthol smokers had statistically significant lower odds than White non-menthol smokers of having made a quit attempt in the past year (AOR=0.91, 95% CI: 0.84, 0.99).

Levy et al. (1) controlled for state smoke-free policies and found that—among participants who were smoking one year prior to the interview—menthol users’ odds of having made a quit attempt in the past year were slightly but statistically significantly higher than non-menthol users’ odds (AOR=1.03, 95% CI: 1.02, 1.03).

Keeler et al. (3) adjusted for census region of residence and found no difference in the odds of having made a past-year quit attempt between menthol and non-menthol smokers (AOR=0.99 (0.94-1.04); p=0.6690).

Rate of Abstinence/Quitting

*Five studies (from six references) that reported rate of abstinence/quitting included meso- and/or macro-level variables. One found that menthol was associated with statistically significant lower rates of cessation, while the other four studies found no difference in rates of cessation between menthol and non-menthol smokers. These findings were consistent with the inconsistency found in the overall results.*

Delnevo et al. analyzed data from the 2003 and 2006-2007 TUS-CPS (22, 23, unpublished). Authors adjusted for living in a state that implemented a cigarette excise tax increase sometime during the previous year in only the narrowest of their “sample restrictions” (past-year cigarette smokers who made a quit attempt or quit; N=24,465). That analysis was the only one of their five to find that the difference between menthol and non-menthol smokers, in the overall sample, in the odds of being a former smoker was not significant (OR=0.92, 95% CI: 0.85, 1.00). Across the racial/ethnic subgroups analyzed, the effect of the narrowest sample restriction was inconsistent: among White smokers, statistically significantly lower odds of being a former smoker were reported among menthol smokers, a result consistent with only one of four other sample restrictions; Hispanic menthol smokers were statistically significantly less likely to be former smokers, contrasting with the other four sample restrictions, which yielded non-significant results; among Mexican smokers, the odds of being a former smoker increased with menthol cigarette use, although the results from the other sample restrictions were inconsistent; Black menthol smokers and Puerto Rican menthol smokers consistently remained statistically significantly less likely to be former smokers across all five sample restrictions.

Hyland et al. (17) adjusted for pricing tier of cigarette smoked (premium, discount, generic) and the presence of another smoker in the household in their analysis of interim data from COMMIT. Menthol was not associated with having been abstinent for the past 6 months (RR=1.00, 95% CI: 0.90, 1.11).

Lewis et al. (19) adjusted for quality preferences (average price per pack), purchase recency (time since last purchase), nicotine levels of cigarettes purchased, and tobacco control metrics (taxation, anti-tobacco advertising, smoke-free policies). Menthol smoking was statistically significantly associated with a decrease in the chance of quitting; specifically, the chance of quitting was 21 percent lower for menthol smokers than non-menthol smokers (HR=0.79, 95% CI: 0.64, 0.99).

Gundersen et al. (25) adjusted for region; they found that menthol was associated with greater odds of being a former (rather than current) smoker; odds of quitting was not associated with menthol cigarette use compared to non-menthol use (AOR=1.05, 95% CI: 0.92, 1.21).

Keeler et al. (3) adjusted for census region of residence and found no difference in the odds of successful cessation (i.e. ≥3 months) between menthol and non-menthol smokers (AOR=0.92 95% CI: 0.83–1.03; p=0.1470); subsample analyses reported similar, non-significant results amongst African-Americans (AOR=1.03, 95% CI: 0.73–1.44; p=0.8630), Whites (AOR=0.94, 95% CI: 0.84–1.06; p=0.3190), Asians (AOR=0.98, 95% CI: 0.44–2.19; p=0.9540), or Hispanics (AOR=0.88, 95% CI: 0.60–1.28; p=0.4980).

Change in Smoking Quantity/Frequency

One study that reported change in smoking quantity/frequency included meso- and macro-level variables in its adjusted analyses. Sawdey et al. (46) controlled for household tobacco use and found no significant difference in the odds of moderate smokers (on 6 to 19 days in the past 30 days) being menthol versus non-menthol smokers (AOR=1.17, 95% CI: 0.86-1.59); however, the odds of frequent smokers (on ≥20 days in the past 30 days) being menthol smokers was significantly higher than being non-menthol smokers (AOR=1.57, 95% CI: 1.08-2.29). The overall p=value across both groups was non-significant (p=0.064).

**Sensitivity Analysis 2: Exclusion of studies rated as having “poor” quality according to the Downs and Black checklist**

*Only one of the included studies presented adjusted results were rated “poor” quality and found that menthol was associated with a statistically significant lower rate of cessation. Removing this study from the overall analysis data did not change the overall findings.*

Thihalolipavan et al. (43) presented a small amount of original data from 13,901 participants in the New York City Nicotine Patch and Gum Program in a letter to the Editor of the *American Journal of Public Health*. The study was rated “poor” quality largely due to lack of information about methodology. This was more a reporting issue than a study quality issue, but given the lack of data, assessment of the latter is impossible. Menthol was associated with a statistically significant lower rate of cessation, measured between three and six weeks after enrollment, compared with non-menthol (PR=0.90, 95% CI: 0.83, 0.97). Removing this study from the overall analysis data did not substantially alter the proportions of studies reporting greater or lower odds of quitting among menthol smokers.

**Sensitivity Analysis 3. Exclusion of studies with potential overadjustment and/or inappropriate adjustment**

*One study contained a variable that indicated past quit attempts in the authors’ model of cessation. Excluding that study from the overall analysis of cessation did not change the original findings.*

Fu et al. (33) analyzed data from a cohort that recruited from five Veterans Administration Medical Centers, all of whom had a recent quit attempt per a pharmacy database, and potentially overadjusted for the cessation measure of quit attempts in past months. They found that—among smokers receiving a quit aid—menthol smokers’ odds of 7-day PPA (self-reported only) at 6-month follow up were no different than non-menthol smokers’ odds (AOR=1.31, 95% CI: 0.95, 1.82).

**References**

1. Schisterman EF, Cole SR, Platt RW. Overadjustment bias and unnecessary adjustment in epidemiologic studies. Epidemiology. 2009;20(4):488-95.

2. Levy DT, Blackman K, Tauras J, Chaloupka FJ, Villanti AC, Niaura RS, et al. Quit attempts and quit rates among menthol and nonmenthol smokers in the United States. Am J Public Health. 2011;101(7):1241-7.

3. Alexander LA, Crawford T, Mendiondo MS. Occupational status, work-site cessation programs and policies and menthol smoking on quitting behaviors of US smokers. Addiction. 2010;105 Suppl 1:95-104.

4. Hyland A, Garten S, Giovino GA, Cummings KM. Mentholated cigarettes and smoking cessation: Findings from COMMIT. Tob Control. 2002;11:135-9.

5. Kahende JW, Malarcher AM, Teplinskaya A, Asman KJ. Quit attempt correlates among smokers by race/ethnicity. International journal of environmental research and public health. 2011;8(10):3871-88.

6. Keeler C, Max W, Yerger V, Yao T, Ong MK, Sung H-Y. The Association of Menthol Cigarette Use With Quit Attempts, Successful Cessation, and Intention to Quit Across Racial/Ethnic Groups in the United States. Nicotine & tobacco research : official journal of the Society for Research on Nicotine and Tobacco. 2017;19(12):1450-64.

7. Delnevo CD, Gundersen DA, Hrywna M, Echeverria SE, Steinberg MB. Smoking-cessation prevalence among U.S. smokers of menthol versus non-menthol cigarettes. Am J Prev Med. 2011;41(4):357-65.

8. Delnevo CD, Gundersen DA, Hrywna M. Examining the relationship between menthol smoking and cessation using data from the 2003 and 2006/7 Tobacco Use Supplement. Center for Tobacco Surveillance and Evaluation Research: University of Medicine & Dentistry of New Jersey - School of Public Health; 2010 January 10-11, 2011.

9. Lewis M, Wang Y, Berg CJ. Tobacco control environment in the United States and individual consumer characteristics in relation to continued smoking: differential responses among menthol smokers? Prev Med. 2014;65:47-51.

10. Gundersen DA, Delnevo CD, Wackowski O. Exploring the relationship between race/ethnicity, menthol smoking, and cessation, in a nationally representative sample of adults. Prev Med. 2009;49(6):553-7.

11. Sawdey MD, Chang JT, Cullen KA, Rass O, Jackson KJ, Ali FRM, et al. Trends and Associations of Menthol Cigarette Smoking Among US Middle and High School Students-National Youth Tobacco Survey, 2011-2018. Nicotine Tob Res. 2020;22(10):1726-35.

12. Thihalolipavan S, Jung M, Jasek J, Chamany S. Menthol smokers in large-scale nicotine replacement therapy program. Am J Public Health. 2014;104(11):e3-4.

13. Fu SS, Okuyemi KS, Partin MR, Ahluwalia JS, Nelson DB, Clothier BA, et al. Menthol cigarettes and smoking cessation during an aided quit attempt. Nicotine Tob Res. 2008;10(3):457-62.
