# Supplementary material for: Assessing the evidence on the differential impact of menthol versus non-menthol cigarette use on smoking cessation in the U.S. population: a systematic review and meta-analysis
Source: Subst Abuse Treat Prev Policy. 2021 Aug 11;16:61. doi: 10.1186/s13011-021-00397-4 (PMC8359586; doi:10.1186/s13011-021-00397-4)
Supplement: Supplementary file 6 — Additional file 6. Characteristics, Definitions, and Covariates of Studies Included in the Meta-Analysis. [file 13011_2021_397_MOESM6_ESM.docx]

**SUPPLEMENTAL SECTION 6: Characteristics, Definitions, and Covariates of Studies Included in the Meta-Analysis**

**SUPPLEMENTAL SECTION 6: Characteristics, Definitions, and Covariates of Studies Included in the Meta-Analysis**

**Adjusted Odds of Reporting a Quit Attempt (Past Year or Ever)**

[Table 6-1: Characteristics of Studies Included in the Meta-Analysis of Quit Attempt from Smoking](#Table6_1)

[Table 6-2: Definitions of Measures, Outcomes and Total Analytic Sample for Studies Included in the Meta-Analysis of Quit Attempt from Cigarette Smoking](#Table6_2)

[Table 6-3: Covariates Controlled for Studies Included in the Meta-Analysis of Cigarette Smoking Quit Attempt](#Table6_3)

**Adjusted Odds of Abstinence (No definition and 7-day PPA)**

[Table 6-4: Characteristics of Studies Included in the Meta-Analysis of Abstinence from Cigarette Smoking](#Table6_4)

[Table 6-5: Definitions of Measures, Outcomes and Total Analytic Sample for Studies Included in the Meta-Analysis of Abstinence from Cigarette Smoking](#Table6_5)

[Table 6-6: Covariates Controlled for Studies Included in the Meta-Analysis of Cigarette Smoking Abstinence](#Table6_6)

**Table 6-1: Characteristics of Studies Included in the Meta-Analysis of Quit Attempt from Smoking**

| Study | Study title | Journal | Survey name | Scope; Population | Special population | Study design |
| --- | --- | --- | --- | --- | --- | --- |
| Alexander et al., 2010 (11) | Occupational status, work-site cessation programs and policies and menthol smoking on quitting behaviors of U.S. smokers | Addiction | TUS-CPS | National (U.S.); Smokers 18 years of age or older | None | Cross-sectional |
| Fagan et al., 2007 (10) | Quit attempts and intention to quit cigarette smoking among young adults in the united states | American Journal of Public Health | TUSCS-CPS | National (U.S.); Young adult current smokers aged 18 to 30 years | None | Cross-sectional |
| Hyland and Rivard, 2010 (16) | Analysis of mentholated cigarettes using the COMMIT data | Unpublished | The COMMIT | U.S.; Current smokers between 25 and 64 years of age at baseline | None | Prospective cohort |
| Kahende et al., 2011 (8) | Quit attempt correlates among smokers by race/ethnicity | International Journal of Environmental Research and Public Health | TUS-CPS | National (U.S.); Adults (≥18 years of age) who had smoked within the past year | None | Cross-sectional |
| Keeler et al., 2017 (3) | The association of menthol cigarette use with quit attempts, successful cessation, and intention to quit across racial/ethnic groups in the united states | Nicotine & Tobacco Research | TUS-CPS | National (U.S.); Adults aged ≥18 who were recent active smokers, defined as current smokers or former smokers who quit less than 12 months ago. | None | Cross-sectional |
| Levy et al., 2011 (1) | Quit attempts and quit rates among menthol and nonmenthol smokers in the united states | American Journal of Public Health | TUS-CPS | National (U.S.); Self-respondents aged 18 years and older | None | Cross-sectional |
| Park, 2017 (5) | Tobacco use behaviors among vulnerable populations | School of Public Health, Indiana University | NATS | National (U.S.); Adults ages 18 years or older | None | Cross-sectional |
| Pletcher et al., 2006 (14) | Menthol cigarettes, smoking cessation, atherosclerosis, and pulmonary function | Archives of internal medicine | CARDIA study | Oakland, Chicago, Minneapolis and Birmingham (U.S.); Healthy adults aged 18 to 30 years | None | Prospective cohort |
| Webb Hooper et al., 2011 (9) | Menthol cigarette smoking and health, Florida 2007 BRFSS | American journal of health behavior | BRFSS | National (U.S.); Civilian adults (≥18 years old) | None | Cross-sectional |

**Table 6-2: Definitions of Measures, Outcomes and Total Analytic Sample for Studies Included in the Meta-Analysis of Quit Attempt from Cigarette Smoking**

| Study | Definition of menthol smoker | Definition of non-menthol smokers | Definition of smoking | Definition of non-smoking | Definition of outcome | Analytic sample |
| --- | --- | --- | --- | --- | --- | --- |
| Alexander et al., 2010 (11) | Self-reported menthol cigarette brand | Self-reported non-menthol cigarette brand | Self-reported current smokers (every day or some days) | NR | Self-reported ever quit attempts of 1 day or longer | 30,176 |
| Fagan et al., 2007 (10) | Self-reported menthol cigarette brand | Self-reported non-menthol cigarette brand | Self-reported current smokers (every day or some days) (answering “every day” or “some days” to question “do you now smoke cigarettes every day, some days, or not at all?”) | Self-reported former smokers (answering “not at all” to question “do you now smoke cigarettes every day, some days, or not at all?”) | Self-reported quit attempts of 1 day or longer in the past year | 6,712 |
| Hyland and Rivard, 2010 (16) | Self-report by answering the question: “What type of cigarettes are the brand that you smoke? Menthol or Plain” | Self-report by answering the question: “What type of cigarettes are the brand that you smoke? Menthol or Plain” | NR | NR | Self-reported ever quit attempts | 2,095 |
| Kahende et al., 2011 (8) | Self-reported menthol cigarette brand | Self-reported non-menthol cigarette brand | Smokers were those who reported smoking at least 100 cigarettes during their lifetimes and smoked within the past year includes those who remained smokers [currently smoked every day or some days] at the time of interview | Former smokers at the time of interview [did not currently smoke] but who reported that they last smoked sometime during the past year | Self-reported past year quit attempts  *Usable data only available for White race/ethnicity, as all other races/ethnicities were compared to it | White=12,794 |
| Keeler et al., 2017 (3) | Answering yes to question “Do you usually smoke menthol or non-menthol cigarettes?” | Answering no to question “Do you usually smoke menthol or non-menthol cigarettes?” | Current smokers were defined as individuals who smoked 100 cigarettes in their lifetime and currently smoke every day (daily smokers) or some days (someday smokers) | Former smokers were defined as individuals who smoked 100 cigarettes in their lifetime but currently do not smoke | Self-reported past year quit attempts | 54,448 |
| Levy et al., 2011 (1) | Self-reported usual menthol cigarette brand | Self-reported usual non-menthol cigarette brand | Individuals who smoked at least 100 cigarettes in their lifetime and were now smoking every day or some days were classified as current smokers | Individuals who smoked at least 100 cigarettes in their lifetime but did not currently smoke were classified as former smokers | Self-reported past year quit attempts | NR |
| Park, 2017 (5) | Current menthol cigarette use was measured from the following two questions: “Have you ever smoked menthol cigarettes for 6 months or more?” and “Currently, when you smoke cigarettes, how often do you smoke menthol cigarettes?” Those who currently smoke menthol cigarettes “all of the time” and “most of the time,” were defined as menthol smokers | Current menthol cigarette use was measured from the following two questions: “Have you ever smoked menthol cigarettes for 6 months or more?” and “Currently, when you smoke cigarettes, how often do you smoke menthol cigarettes?” Those who currently smoke menthol cigarettes “some of time” “rarely” or those who have never smoked menthol cigarettes for 6 months or more were defined as non-menthol smokers. | Respondents who had smoked more than 100 cigarettes in their lifetime and currently smoke cigarettes every day or some days | NR | Likelihood of past-year quit attempts | 4,245 |
| Pletcher et al., 2006 (14) | Answering “mentholated” to question “Is [your current brand of cigarettes] mentholated or nonmentholated?” | Answering “nonmentholated” to question “Is [your current brand of cigarettes] mentholated or nonmentholated?” | NR | NR | Self-reported ever quit attempts | 6,636*  *Number of examinations |
| Webb Hooper et al., 2011 (9) | Answering “menthol” to the question “Is your usual cigarette brand menthol or nonmenthol?” | Answering “nonmenthol” to the question “Is your usual cigarette brand menthol or nonmenthol?” | Current smokers indicated that they smoked at least 100 lifetime cigarettes and smoked every day or some days | NR | Self-reported past year quit attempts | 2,977 |

**Table 6-3: Covariates Controlled for Studies Included in the Meta-Analysis of Cigarette Smoking Quit Attempt**

| Study | Characteristics of population | Micro Factors | Meso Factors | Macro Factors |
| --- | --- | --- | --- | --- |
| Alexander et al., 2010 (11) | Smokers 18 years of age or older from U.S. | Age  Race/ethnicity  Gender  Education  Region |  |  |
| Fagan et al., 2007 (10) | Young adult current smokers aged 18 to 30 years from U.S. | Age  Gender  *Following covariates were adjusted for if independent variables in the bivariate models had a p <0.25:  Race/ethnicity  Employment status  Enrolled in school last week  Annual family income  Smoking status |  |  |
| Hyland and Rivard, 2010 (16) | Current smokers between 25 and 64 years of age at baseline from U.S. | Gender  Age  Race/ethnicity  Education  Frequency of alcohol consumption in 1988  Age started smoking  Amount smoked in 1993  Number of past quit attempts  Other smokers in the household  Desire to quit smoking in 1988 |  |  |
| Kahende et al., 2011 (8) | Adults (≥18 years of age) who had smoked within the past year from U.S. | Gender  Age  Education  Region  Number of CPD  How long smoked | Smoking policy at work |  |
| Keeler et al., 2017 (3) | Adults aged ≥18 for national (U.S.) survey | Age  Gender  Education  Smoking less than 30 min after waking  Cigarette type  Survey year  Family income  Marital status  Region  Race/ethnicity |  |  |
| Levy et al., 2011 (1) | Self-respondents aged 18 years and older from U.S. | Age  Gender  Race/ethnicity  Marital status  Education  Family income  Survey year |  | State-level tobacco control policies |
| Park, 2017 (5) | Adults ages 18 years or older from U.S. | Menthol cigarette use  Age  Race/ethnicity  Gender  Marital status  Education  Household income  Quantity of smoking per day  Smoking onset | Home smoking restrictions | Statewide comprehensive smoke-free laws |
| Pletcher et al., 2006 (14) | Healthy adults aged 18 to 30 years from Oakland, Chicago, Minneapolis and Birmingham (U.S.) | Age  Gender  Race/ethnicity  CPD at baseline  Educational level  Marital status  Employment  Health insurance status |  |  |
| Webb Hooper et al., 2011 (9) | Civilian adults (≥18 years old) from U.S. | Race/ethnicity  Age  Gender  Smoked pack years  Nicotine dependence  Ever had medical condition  Any health coverage  Frequency poor mental health  Frequency poor physical health |  |  |

**Table 6-4: Characteristics of Studies Included in the Meta-Analysis of Abstinence from Cigarette Smoking**

| Study | Study title | Journal | Survey name | Scope; Population | Special population | Study design |
| --- | --- | --- | --- | --- | --- | --- |
| Blot et al., 2011 (24) | Lung cancer risk among smokers of menthol cigarettes | Journal of the National Cancer Institute | Southern Community Cohort Study | Alabama, Arkansas, Florida, Georgia, Kentucky, Louisiana, Mississippi, North Carolina, South Carolina, Tennessee, Virginia, and West Virginia (U.S.); Adults aged 40–79 years | None | Prospective cohort |
| Delnevo et al., 2011 (22) | Smoking-Cessation prevalence among U.S. smokers of menthol versus non-menthol cigarettes | American Journal of Preventive Medicine | Analysis of 2003 and 2006/2007 TUS CPS | National (U.S.); Adult smokers ≥18 years old | None | Cross-sectional |
| Faseru et al., 2013 (28) | Predictors of cessation in African American light smokers | Addictive Behaviors | None | Kansas City Metropolitan area (U.S.); African American light smokers ≥18 years old | None | Prospective cohort |
| Foulds et al., 2006 (34) | Factors associated with quitting smoking | American Journal of Health Behavior | None | New Jersey (U.S.); First 1021 patients who attempted to quit | None | Prospective cohort |
| Fu et al., 2008 (33) | Menthol cigarettes and smoking cessation during an aided quit attempt | Nicotine & Tobacco Research | None | U.S. (national); Veterans ≥19 years old who had received a prescription for nicotine replacement therapy (NRT) or bupropion for smoking cessation between February and October 2002 from one of five participating VA medical centers were randomly assigned to either the intervention or usual care. | Veterans | Prospective cohort |
| Gandhi et al., 2009 (32) | Lower quit rates among African American and Latino menthol cigarette smokers at a tobacco treatment clinic | International journal of clinical practice | None | New Jersey (U.S.); Specialist tobacco dependence treatment outpatient clinic patients | None | Retrospective cohort |
| Gundersen et al., 2009 (25) | Exploring the relationship between race/ethnicity, menthol smoking, and cessation, in a nationally representative sample of adults" | Preventative Medicine | Analysis of 2005 U.S. National Health Interview Survey - Cancer Control Supplement (NHIS-CCS) | National (U.S.); Adult smokers ≥18 years old | None | Cross-sectional |
| Okuyemi et al., 2003 (35) | Does menthol attenuate the effect of bupropion among African American smokers? | Addiction | None | Kansas City Metropolitan area (U.S.); African American smokers ≥18 years old | None | Prospective cohort |
| Okuyemi et al., 2007 (36) | Relationship between menthol cigarettes and smoking cessation among African American light smokers | Addiction | None | Kansas City Metropolitan area (U.S.); African American light smokers ≥18 years old | None | Prospective cohort |
| Pletcher et al., 2006 (14) | Menthol cigarettes, smoking cessation, atherosclerosis, and pulmonary function: the Coronary Artery Risk Development in Young Adults (CARDIA) Study | Archives of internal medicine | Coronary Artery Risk Development in Young Adults (CARDIA) Study | Oakland, Chicago, Minneapolis, Birmingham (U.S.); Adults aged 18 to 30 years and healthy at the time of enrollment in 1985 | None | Prospective cohort |
| Rojewski et al., 2014 (27) | Menthol cigarette use predicts treatment outcomes of weight-concerned smokers | Nicotine & Tobacco Research | None | Connecticut (U.S.); weight-concerned female smokers | Smokers concerned about gaining weight after quitting and smoking to manage weight | Prospective cohort |
| Steinberg et al., 2011 (30) | Abstinence and psychological distress in co-morbid smokers using various pharmacotherapies | Drug and Alcohol Dependence | None | New Jersey (U.S.); smokers presenting for treatment | None | Retrospective cohort |

**Table 6-5: Definitions of Measures, Outcomes and Total Analytic Sample for Studies Included in the Meta-Analysis of Abstinence from Cigarette Smoking**

| Study | Definition of menthol smoker | Definition of non-menthol smoker | Definition of smoking | Definition of non-smoking | Definition of outcome | Analytic sample |
| --- | --- | --- | --- | --- | --- | --- |
| Blot et al., 2011 (24) | Answering yes to "Are the cigarettes you usually smoke menthol?” | Answering no to "Are the cigarettes you usually smoke menthol?” | Smoked at least 100 cigarettes in their lifetime | NR | Prospectively by computing quit rates ascertained from the follow-up interviews | NR |
| Delnevo et al., 2011 (22) | Usual brand of cigarettes in the past 12 months was mentholated | Usual brand of cigarettes in the past 12 months was non-mentholated | 100 cigarettes in a lifetime and smoking "everyday" or "some days" at the time of the survey | "A former smoker was defined as having smoked 100 cigarettes in a lifetime and smoking 'not at all' at the time of the survey" | "The outcome variable was smoking cessation operationalized as current versus former smoker (0=current, 1=former)" | 71,193 |
| Faseru et al., 2013 (28) | "Smokes menthol cigarettes" - Yes | "Smokes menthol cigarettes" - No | ≤10 CPD for ≥ 2 years, and have smoked on ≥ 25 days in the month prior to enrollment | No cigarettes (not even a puff) in the previous 7 days at Week 7", and a cotinine cut-point of 15ng/mL | No cigarettes (not even a puff) in the previous 7 days at Week 7", and a cotinine cut-point of 15ng/mL | 540 |
| Foulds et al., 2006 (34) | "Current brand menthol" - yes | "Current brand menthol" - no | NR | Self-report of no smoking in the past week and CO level below 10 parts per million (ppm) | The primary outcome was 7-day point abstinence rates (answering 'no' to the question, 'Have you used any tobacco in the past 7 days? )'" at 4 weeks | 1,021 |
| Fu et al., 2008 (33) | Smoked menthol cigarettes 2 years ago | Did not smoke menthol cigarettes 2 years ago | NR | NR | "The primary outcome for the current study was self-reported 7-day point prevalent smoking abstinence" | 1,343 |
| Gandhi et al., 2009 (32) | Current cigarette type reported to be mentholated | Current cigarette type reported to be non-mentholated | NR | NR | The primary outcome was self-reported 7-day point prevalence abstinence rate (answering ‘no’ to the question, ‘Have you used any tobacco in the past 7 days?’) | 1,688  White: 1,086  Black: 374  Hispanic/Latino: 149  Other: 79 |
| Gundersen et al., 2009 (25) | Self-reported that their usual brand of cigarettes in the past 12 months or in the 12 months prior to quitting was mentholated | Self-reported that their usual brand of cigarettes in the past 12 months or in the 12 months prior to quitting was non-mentholated | 100 cigarettes in a lifetime and smoking "everyday" or "some days" at the time of the survey | "A former smoker is defined as having smoked 100 cigarettes in a lifetime and now smoking 'not at all.” | "The outcome variable is cessation operationalized as current vs. former smoker (0=current, 1=former)" | 7,815 |
| Okuyemi et al., 2003 (35) | Answering "yes" to "Do you usually smoke menthol cigarettes?" | Answering "no" to "Do you usually smoke menthol cigarettes?" | At least 10 CPD | NR | Self-reported 7-day point prevalence smoking cessation confirmed with eCO <10 ppm and only in the case of discrepancies salivary cotinine <20 ug/ml at 6 weeks | 390 |
| Okuyemi et al., 2007 (36) | Self-reported use of menthol cigarettes | Self-reported use of non-menthol cigarettes | Smoke ≤10 CPD for at least 6 months; smoke cigarettes on ≥25 of the last 30 days | NR | 7-day point-prevalence smoking cessation defined as having smoked no cigarettes-not even a puff-for the previous 7 days, confirmed with salivary cotinine ≤20 ng/ml and eCO ≤10 ppm at 26 weeks | < 50 age group: N=NR; ≥ 50 years: N=NR |
| Pletcher et al., 2006 (14) | Answering mentholated to question "Is [your current brand of cigarettes] mentholated or non-mentholated?" | Answering non-mentholated to question "Is [your current brand of cigarettes] mentholated or non-mentholated?" | NR | NR | Sustained smoking cessation (no current smoking the past 2 times they were examined in The CARD1A Study) | 1,260 |
| Rojewski et al., 2014 (27) | "Defined by a response on a smoking history questionnaire of “menthol” (vs. nonmenthol) when asked about the cigarettes participants currently smoke most of the time." | Answered "nonmenthol" when asked about the cigarettes participants currently smoke most of the time | Smoking to manage weight | NR | Self-reported 7-day point abstinence confirmed with eCO ≤10 ppm at 14 weeks | 166 |
| Steinberg et al., 2011 (30) | NR | NR | eCO greater than 10ppm | eCO less than 10ppm | Self-reported 7-day point abstinence confirmed with eCO cut-off of 10ppm at 26 weeks | 579 |

**Table 6-6: Covariates Controlled for Studies Included in the Meta-Analysis of Cigarette Smoking Abstinence**

| Study | Characteristics of population | Micro Factors | Meso Factors | Macro Factors |
| --- | --- | --- | --- | --- |
| Blot et al., 2011 (24) | Adults aged 40–79 years from Alabama, Arkansas, Florida, Georgia, Kentucky, Louisiana, Mississippi, North Carolina, South Carolina, Tennessee, Virginia, and West Virginia (U.S.) | Age  Gender  Race/ethnicity  Income  Education  Body mass index  Pack-years of smoking at entry into the cohort  Type of cigarette (menthol or non-menthol) | Time elapsed between the baseline and follow-up questionnaires  Recruitment source |  |
| Delnevo et al., 2011 (22) | Adults ≥18 years old from U.S. (national) | Age  Gender  Race/ethnicity  Hispanic origin  Education  Smoking status  Year of survey  Month of survey |  | Cigarette excise tax increase |
| Faseru et al., 2013 (28) | African American light smokers of 18 years or older from Kansas City metropolitan area (U.S.) | Treatment  Type of cigarette  Visit attendance  Cotinine level  Years smoked  *Age and gender were found to be not significant in univariate analysis and therefore were not used in adjustments |  |  |
| Foulds et al., 2006 (34) | Patients using a specialist tobacco dependence treatment outpatient clinic to quit tobacco in New Jersey (U.S.) | Age  Gender  Race/ethnicity  Marital status  Education  Employment status  Number of children  Type of insurance  Age first used tobacco  Age started daily smoking  Years of daily tobacco use  CPD  Time to first smoke in the morning  Awakens at night to smoke  Prior attempts to quit smoking  Current brand light or low tar  Current brand menthol  Baseline stage of change (Current quit attempt)  Any disease or condition caused or aggravated by tobacco use  Previous treatment for a mental health or behavioral problem  Previous treatment for alcohol or other drug problem  Number of face-face contacts at the clinic  *backward stepwise procedure entered all variables initially and removed one variable at a time (less significant first) until all had a value of p<0.1. | Referral to the clinic |  |
| Fu et al., 2008 (33) | Veterans ≥19 years old in national sample (U.S.) | Race/ethnicity  Gender  Site  Age  Intervention vs. usual care group  History of smoking-related cancer  History of substance abuse disorder  Additional 24-hour quit attempts in past 12 months |  |  |
| Gandhi et al., 2009 (32) | Specialist tobacco dependence treatment outpatient clinic patients from New Jersey (U.S.) | Age  Education  Gender  Employment status  Type of insurance  CPD  Age smoked for first time  Awaken at night to smoke  Time to use first cigarette of day  Previous attempts to quit smoking  Use of mentholated cigarettes  Presence of disease caused or aggravated by smoking |  |  |
| Gundersen et al., 2009 (25) | Adults ≥18 years old from U.S. (national) | Age  Gender  Education  CPD  Perceived risk of cancer | Census region |  |
| Okuyemi et al., 2003 (35) | African American smokers of 18 years or older from Kansas City metropolitan area (U.S.) | Gender  Monthly income  Education  Employment  CPD  FTND score  Satisfaction of most recent cigarette  Taste of most recent cigarette  Salivary cotinine  eCO  *Stepwise logistic regression and best subsets logistic regression were used to explore the joint relationship of treatment, menthol cigarette smoking and the other baseline variables |  |  |
| Okuyemi et al., 2007 (36) | Kansas City Metropolitan area; African American light smokers ≥18 years old | Gender  Monthly income  Marital status  Monthly family income  Education  Employment  CPD in past 7 days  Age started smoking regularly  Duration of smoking  Number of 24-hour quit attempts in the past year  Longest single quit period  Motivation to quit  Confidence in quitting  NDSS  Minnesota Withdrawal Score  eCO  Serum cotinine  *Stepwise logistic regression and best subsets logistic regression were used to explore the joint relationship of treatment, baseline variables (except age) and menthol cigarette smoking on the abstinence outcome |  |  |
| Pletcher et al., 2006 (14) | Adults aged 18 to 30 years and healthy at the time of enrollment in 1985 from Oakland, Chicago, Minneapolis, Birmingham (U.S.) | Age  Gender  Race/ethnicity  Educational level  Marital status  Employment  Health insurance status  CPD at baseline |  |  |
| Rojewski et al., 2014 (27) | Female smokers from Connecticut (U.S.) smoking to manage weight | Age |  |  |
| Steinberg et al., 2011 (30) | Smokers from New Jersey (U.S.) presenting for treatment | Age  Gender  Race/ethnicity  Education  CPD  Employment  Marital status  Night smoking |  |  |

**References**

1. Levy DT, Blackman K, Tauras J, Chaloupka FJ, Villanti AC, Niaura RS, et al. Quit attempts and quit rates among menthol and nonmenthol smokers in the United States. Am J Public Health. 2011;101(7):1241-7.

3. Keeler C, Max W, Yerger V, Yao T, Ong MK, Sung H-Y. The Association of Menthol Cigarette Use With Quit Attempts, Successful Cessation, and Intention to Quit Across Racial/Ethnic Groups in the United States. Nicotine & tobacco research : official journal of the Society for Research on Nicotine and Tobacco. 2017;19(12):1450-64.

5. Park J-Y. Tobacco use behaviors among vulnerable populations. Dissertation Abstracts International: Section B: The Sciences and Engineering. 2017;77(12-B(E)):No-Specified.

8. Kahende JW, Malarcher AM, Teplinskaya A, Asman KJ. Quit attempt correlates among smokers by race/ethnicity. International journal of environmental research and public health. 2011;8(10):3871-88.

9. Webb Hooper M, Zhao W, Byrne MM, Davila E, Caban-Martinez A, Dietz NA, et al. Menthol cigarette smoking and health, Florida 2007 BRFSS. Am J Health Behav. 2011;31(1):3-14.

10. Fagan P, Augustson E, Backinger CL, O'Connell ME, Vollinger RE, Jr., Kaufman A, et al. Quit attempts and intention to quit cigarette smoking among young adults in the United States. Am J Public Health. 2007;97(8):1412-20.

11. Alexander LA, Crawford T, Mendiondo MS. Occupational status, work-site cessation programs and policies and menthol smoking on quitting behaviors of US smokers. Addiction. 2010;105 Suppl 1:95-104.

14. Pletcher MJ, Hulley BJ, Houston T, Kiefe CI, Benowitz N, Sidney S. Menthol cigarettes, smoking cessation, atherosclerosis, and pulmonary function. Arch Intern Med. 2006;166:1915-22.

16. Hyland A, Rivard C. Analysis of mentholated cigarettes using the COMMIT data -- summary. Department of Health Behavior, Roswell Park Cancer Institute; 2010.

22. Delnevo CD, Gundersen DA, Hrywna M, Echeverria SE, Steinberg MB. Smoking-cessation prevalence among U.S. smokers of menthol versus non-menthol cigarettes. Am J Prev Med. 2011;41(4):357-65.

24. Blot WJ, Cohen SS, Aldrich M, McLaughlin JK, Hargreaves MK, Signorello LB. Lung cancer risk among smokers of menthol cigarettes. J Natl Cancer Inst. 2011;103(10):810-6.

25. Gundersen DA, Delnevo CD, Wackowski O. Exploring the relationship between race/ethnicity, menthol smoking, and cessation, in a nationally representative sample of adults. Prev Med. 2009;49(6):553-7.

27. Rojewski AM, Toll BA, O'Malley SS. Menthol cigarette use predicts treatment outcomes of weight-concerned smokers. Nicotine Tob Res. 2014;16(1):115-9.

28. Faseru B, Nollen NL, Mayo MS, Krebill R, Choi WS, Benowitz NL, et al. Predictors of cessation in African American light smokers enrolled in a bupropion clinical trial. Addict Behav. 2013;38(3):1796-803.

30. Steinberg MB, Bover MT, Richardson DL, Schmelzer AC, Williams JM, Foulds J. Abstinence and psychological distress in co-morbid smokers using various pharmacotherapies. Drug Alcohol Depend. 2011;114(1):77-81.

32. Gandhi KK, Foulds J, Steinberg MB, Lu SE, Williams JM. Lower quit rates among African American and Latino menthol cigarette smokers at a tobacco treatment clinic. Int J Clin Pract. 2009;63(3):360-7.

33. Fu SS, Okuyemi KS, Partin MR, Ahluwalia JS, Nelson DB, Clothier BA, et al. Menthol cigarettes and smoking cessation during an aided quit attempt. Nicotine Tob Res. 2008;10(3):457-62.

34. Foulds J, Gandhi KK, Steinberg MB, Richardson DL, Williams JM, Burke MV, et al. Factors associated with quitting smoking at a tobacco dependence treatment clinic. Am J Health Behav. 2006;30(4):400-12.

35. Okuyemi KS, Ahluwalia JS, Ebersole-Robinson M, Catley D, Mayo MS, Resnicow K. Does menthol attenuate the effect of bupropion among African American smokers? Addiction. 2003;98:1387-93.

36. Okuyemi KS, Faseru B, Sanderson Cox L, Bronars CA, Ahluwalia JS. Relationship between menthol cigarettes and smoking cessation among African American light smokers. Addiction. 2007;102(12):1979-86.
